# Functional Analysis of Anuran Pelvic and Thigh Anatomy Using Musculoskeletal Modelling of *Phlyctimantis maculatus*

**DOI:** 10.3389/fbioe.2022.806174

**Published:** 2022-04-01

**Authors:** A. J. Collings, E. A. Eberhard, C. Basu, C. T. Richards

**Affiliations:** ^1^ School of Health and Life Sciences, Teesside University, Middlesbrough, United Kingdom; ^2^ Structure and Motion Laboratory, Royal Veterinary College, Hatfield, United Kingdom; ^3^ Swiss Federal Institute of Technology Lausanne, Lausanne, Switzerland; ^4^ School of Veterinary Medicine, University of Surrey, Guildford, United Kingdom

**Keywords:** Anuran, Pelvis, Musculoskeletal modelling, Moment arm, Walking locomotion, Pelvic lateral rotation, MuJoCo

## Abstract

Using their abundant musculature, frogs are able to exhibit outstanding behavioural versatility. However, understanding the dynamic motion of their 30 + hindlimb muscles, with multi-joint action, and curved pathways, is challenging. This is particularly true in walking, a relatively understudied, but complex frog gait. Building on prior musculoskeletal modelling work we construct and analyse a 3D musculoskeletal model of the spine, pelvis, and hindlimb of *Phlyctimantis maculatus* (previously known as *Kassina maculata*) to simulate the natural motion of muscle pathways as joints rotate during locomotion. Combining experimental kinematics and DICE-CT scan data we use several simulations conducted in MuJoCo to decouple femur and pelvic motions, generating new insights into the functional mechanics of walking in frogs. Outputs demonstrate pelvic lateral rotation about the iliosacral joint influences moment arm magnitude in the majority of hindlimb muscles. The extent of pelvic influence depends on femoral angle which changes muscle function in some instances. The workflow presented here can be used to help experimentalists predict which muscles to probe with *in vivo* techniques towards a better understanding of how anuran musculoskeletal mechanics enable multiple behaviours.

## Introduction

Frogs use their abundant musculature to exhibit astonishing behavioural versatility (Kargo and Giszter, 2000; Kargo et al., 2002). To coordinate these numerous muscles, frogs (and other vertebrates) activate groups of muscles (“synergies”) to elegantly control motion (d'Avella and Lacquaniti, 2013). Yet, over a century ago, Lombard and Abbott proposed that motor signals do not fully explain limb motion, stating " [limb movements] *have the appearance of being the result of finely adjusted nervous coordinations, are really due to the mechanical conditions under which the muscles act on the bones.*” (Lombard and Abbott, 1907). Hence, elucidating not only the neurological, but also the biomechanical properties is crucial for understanding limb function.

Unfortunately, understanding frog hindlimbs is challenging; they have 30 + muscles, several crossing multiple joints, and following curved pathways around bones or other muscles (Dunlap, 1960; Duellman and Trueb, 1986; Kargo et al., 2002; Přikryl et al., 2009; Collings and Richards, 2019). Additionally, unlike limbs that move in simple planar motion (e.g. mice; Mendes et al., 2015) frog hindlimbs move simultaneously in three planes (Astley & Roberts, 2014; Richards et al., 2017; Collings et al., 2019), possibly causing time-varying shifts in muscle action (Lombard & Abbott, 1907). For example, the mouse semimembranosus is a simple hip extensor/knee flexor (Charles et al., 2016), whereas the frog semimembranosus shifts from knee flexor to extensor, likely due to the out-of-plane movements of the shank (Lombard & Abbott, 1907). Hence, we are unlikely to determine limb mechanical function simply by studying muscle attachments.

*In situ* experimentation directly measures muscle actions about joints *via* electrical stimulation (e.g. Přikryl et al., 2009) or by moment arm measurements (e.g. Lieber and Boakes, 1988). However, such approaches are limited because they rarely consider simultaneous action from multiple muscles (although see Lombard & Abbott, 1907). Furthermore, moment arm distances can vary with joint angle meaning that postural changes can alter a muscle’s contribution to joint torque (e.g., Lieber and Shoemaker, 1992). In these cases where parameters are too numerous to cover in an experimental context, modelling approaches are useful.

In the spirit of prior musculoskeletal modelling work (e.g. OpenSim; Delp et al., 2007; Kargo et al., 2002; Kargo and Rome, 2002) we construct and analyse a 3D musculoskeletal model of the spine, pelvis, and hindlimb of *Phlyctimantis maculatus* (previously known as *Kassina maculata;* common name the red legged running frog) to simulate the natural motion of muscle pathways as joints rotate during frog walking. *P. maculatus* ideal because it is a habitual walker (Ahn et al., 2004; Reynaga et al., 2018; Collings et al., 2019). In this species, stride length is mainly driven by horizontal motion of the femur during walking (Collings et al., 2019) which is powered by intricate musculature linking the pelvis to the leg (Lombard & Abbott, 1907; Kargo and Rome, 2002; Přikryl et al., 2009; Collings and Richards, 2019). Horizontal motion of the femur is coupled with lateral rotation of the pelvis (Emerson and De Jongh, 1980; Collings et al., 2019). Originally thought to increase stride length, pelvic lateral rotation has been shown recently to have a relatively unsubstantial effect on stride length during walking (Collings et al., 2019). However, before this lateral rotation was discovered, Lombard and Abbott observed that the actions of femoral muscles change drastically depending on the angle between the pelvis and femur (Lombard and Abbott, 1907). Given the importance of femoral motion in walking, we therefore hypothesise that pelvic lateral rotation increases muscle moment arms of femoral muscles during the walking stride cycle.

Using experimental kinematics (Collings et al., 2019) and a DICE CT model (Collings and Richards, 2019) we produced 1) a range of hypothetical simulations decoupling femur and pelvic rotations and 2) kinematics-driven simulations based on a typical walking trial. We found that muscles crossing the hips are most effective in flexion/extension or long-axis rotation (as opposed to abduction/adduction). Furthermore, their moment arm magnitudes were impacted by both femoral angle and pelvic lateral rotation during walking. Additionally, the axial muscle mechanics were influenced by lateral and dorsoventral rotation at the iliosacral (IS) joints. Our study thus demonstrates how computational kinematic reconstruction can non-invasively estimate time-varying muscle moment arms in vertebrate limbs. We propose that our workflow can be used to generate detailed predictions to help experimentalists determine which muscles to probe with *in vivo* techniques. To our knowledge, this is the first study to quantify moment arms in a walking frog and, hence, opens an important opportunity to better understand how their musculoskeletal mechanics enable multiple behaviours.

## Materials and Methods

**FIGURE 1:**
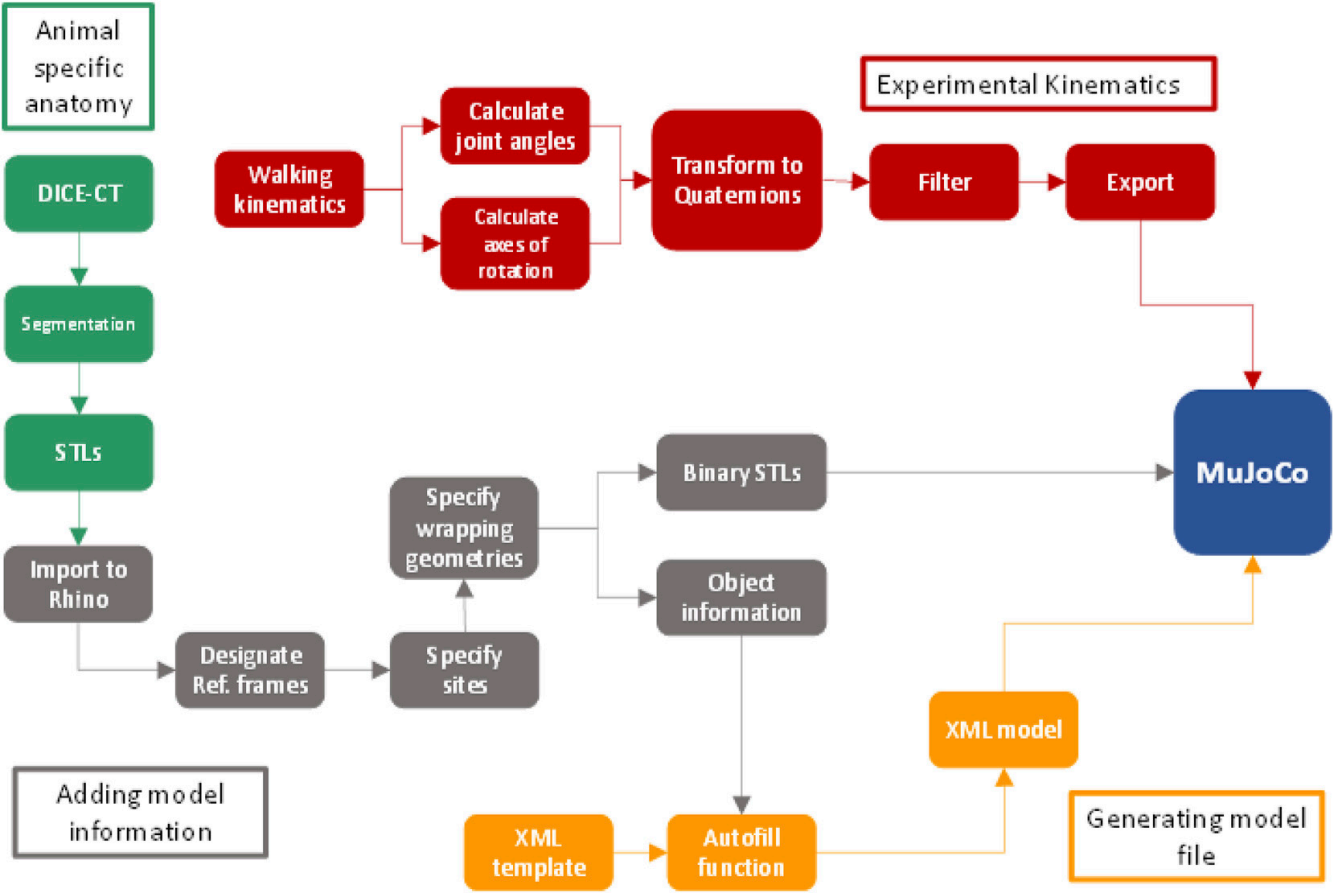
Workflow diagram depicting the required steps for data collection from animal to model.

We built a musculoskeletal model of the pelvis and hindlimb based on a *Phlyctimantis maculata* (formerly *Kassina maculata*) specimen (Collings and Richards, 2019). The model consists of 6 separate skeletal components, 16 muscles, and 5 joints (Table 1). To animate the model, we used a forward kinematics approach where joint angle data drive the motions of the joints and musculature. The methods applied to building the model required several steps, presented as a workflow diagram (Figure 1). To generate our simulation three main data inputs are required: 1) skeletal morphology, 2) joint kinematics data (experimental or hypothetical) and 3) a document specifying both the model topology (i.e., specific connections of muscles and joints to bones) as well as the dimensions and proportions of all elements. The morphology information includes 3D surface geometry of the specimen skeleton. Joint kinematics data is in the form of joint angles (specifically Euler angles or quaternions). Finally, the anatomical information required to assemble all musculoskeletal elements is specified in an XML template file. Further anatomical information such as the joint centres of rotation and specific muscle attachment sites are input into this template to generate an XML model. Together, the 3D surface geometry and the XML model generate a model ‘puppet’ containing only information defining the geometry of the musculoskeletal system, but no information regarding posture of joint orientations. Kinematics data, either experimental or hypothetical, is then input along with the 3D skeletal geometry and the XML model to generate a simulation using the physics engine MuJoCo (Todorov et al., 2012). Although MuJoCo is conventionally used to solve forward/inverse dynamics problems, it can also be used for kinematics computations. In the present study, we apply MuJoCo’s forward kinematics function (part of its larger dynamics pipeline) to compute the positions of the bone segments and corresponding muscle moment arms. We note that the XML template presented in this study can be used as a generalised template for any frog specimen from spine to tarsometatarsus (TMT) and any kinematics data set can be input into this workflow providing it appears in the same format, therefore allowing investigation of any frog species (extinct or extant), not only *P. maculatus*. Specific anatomy obtained through traditional dissection and DICE µCT techniques (Collings and Richards, 2019) were used to generate the model puppet which was then animated using representative experimental kinematics from a prior study (Collings et al., 2019). Here we used an example trial closest representing the average joint kinematics of the walking trials in Collings et al. (2019).

**TABLE 1:** A summary table listing the bones, muscles, and joints modelled. Note the predicted functions of the muscles are based on published data from Přikryl et al. (2009).

Model Components	MTU	Abbreviation	Further Information
Bones	Spine and Sacrum	N/A	Mesh consisted of the five vertebral elements modelled as one unit
	Urostyle		Single bone continuous with sacrum forming sacrourostylic joint
	Pelvis		Single bone forming paired iliosacral joints
	Femur		Single bone forming hip joint. Left femur aminated with kinematics. Right femur mirrored
	Tibiofibula		Fused paired bones forming knee joint. Left tibiofibular animated with kinematics. Right is mirrored
	Tarsals		Paired bones forming ankle joint. Left tarsals animated with kinematics. Right are mirrored
Muscles	Coccygeoiliacus (right)	CI (right)	MTUs: 3; proximal middle, distal
	Coccygeoiliacus (left)	CI (left)	MTU colour: Dark green
			Attachment sites: Iliac shaft and urostyle shaft
			*Via* sites: None
			Multiarticular: No
			Predicted function: Pelvic lateral and dorsoventral rotation
	Iliolumbaris (right)	IL (right)	MTUs: 4
	Iliolumbaris (left)	IL (left)	MTU colour: Golden yellow
			Attachment sites: Pre-sacral vertebrae and proximal ilia tips
			*Via* sites: None
			Multiarticular: No
			Predicted function: Pelvic lateral rotation, anterior-posterior sliding of ilia, spinal bending
	Iliacus externus	IE	MTUs: 1
			MTU colour: Red
			Attachment sites: Proxima iliac shaft and proximal femur
			*Via* sites: One at distal ilium
			Multiarticular: No
			Predicted function: Hip flexion
	Semimembranosus	SM	MTUs: 1
			MTU colour: Yellow
			Attachment sites: Ischial/iliac rim and lateral tibiofibula
			*Via* sites: None
			Multiarticular: Yes
			Predicted function: Hip extension
	Iliofibularis	IFB	MTUs: 1
			MTU colour: Light blue
			Attachment sites: Distal ilium and lateral tibiofibula
			*Via* sites: None
			Multiarticular: Yes
			Predicted function: Hip extension
	Obturator externus	OE	MTUs: 1
			MTU colour: Dark blue
			Attachment sites: Ischium (ventral border) and femur (mid-shaft)
			*Via* sites: None
			Multiarticular: No
			Predicted function: Hip extension
	Sartorius	SA	MTUs: 1
			MTU colour: Deep red-purple
			Attachment sites: Ischium (ventral border) and medial tibiofibula
			*Via* sites: None
			Multiarticular: Yes
			Predicted function: Hip flexion and adduction
	Adductor longus	AL	MTUs: 1
			MTU colour: Teal
			Attachment sites: Ischium (ventral border) and medial tibiofibula
			*Via* sites: None
			Multiarticular: Yes
			Predicted function: Hip flexion and adduction
	Adductor magnus	AM	MTUs: 2?
			MTU colour: Light mint green
			Attachment sites: Ischium (ventral border) and distal femur
			*Via* sites: None
			Multiarticular: No
			Predicted function: Hip flexion and adduction
	Gracilis major	GR	MTUs: 1
			MTU colour: Grey
			Attachment sites: Ischium and medial tibiofibula
			*Via* sites: None
			Multiarticular: Yes
			Predicted function: Hip extension and adduction
	Iliofemoralis	IFM	MTUs: 1
			MTU colour: Dark blue
			Attachment sites: Ilium (ventral border) and femur (mid-shaft)
			*Via* sites: None
			Multiarticular: No
			Predicted function: Hip extension and adduction
	Iliacus internus	II	MTUs: 2
			MTU colour: Light orange
			Attachment sites: Distal ilium (dorsal surface) and proximal femur
			*Via* sites: Distal ilium (ventral surface)
			Multiarticular: No
			Predicted function: Hip flexion and abduction
	Pyriformis	PY	MTUs: 1
			MTU colour: Light red
			Attachment sites: Distal urostyle and proximal femur
			*Via* sites: No
			Multiarticular: No
			Predicted function: Hip abduction
	Cruralis and Gluteus maximus	CR/GL	MTUs: 1
			MTU colour: Pink and Purple
			Attachment sites: Ilium and anterior tibiofibula
			*Via* sites: No
			Multiarticular: Yes
			Predicted function: Knee extension and hip flexion
Joints	Sacrourostylic	SU	Modelled joint type: Hinge
			Degrees of freedom: 2
			Motion permitted: Lateral and dorsoventral rotation
	Sacroiliac (right)	IS (right)	Modelled joint type: Double hinge
	Sacroiliac (left)	IS (left)	Degrees of freedom: 2
			Motion permitted: Lateral and dorsoventral rotation
	Hip	N/A	Modelled joint type: Ball
			Degrees of freedom: 3
			Motion permitted: Flexion/extension, adduction/abduction, long axis rotation
	Knee		Modelled joint type: Rolling
			Degrees of freedom: 2 motion permitted: Flexion/extension, fore-aft translation
	Ankle		Modelled joint type: Ball
			Degrees of freedom: 3
			Motion permitted: Flexion/extension, adduction/abduction, long axis rotation

### Compiling Model Object Information

3D meshes of all bones (excluding all bones distal to the TMT joint), and muscles of the spine, pelvis, and left femur [excluding tensor fascia latae (TFL), pectineus (PEC), quadratus femoris (QF), gemellus (GE), and obturator internus (OI) muscles] were exported as STL files and subsequently loaded into Rhinoceros 3D (Version 5 SR14, Robert McNeel and Associates, Barcelona, Spain). Bones of the digits were excluded due to their complexity and the fact that foot-ground interactions were not the focus of the present study. Only muscles acting about the hip joint were included. The small hip muscles and those encasing the femoral head (TFL, GE, OI, QF) were excluded due to their very small size. Each STL mesh was scaled around the origin according to the scan resolution (17.64 µm). The following information was then compiled; approximate joint centre of rotation, segment local reference frames, muscle attachment and *via* sites, and wrapping geometries and side sites, each of these steps and terminologies are explained below.

### Joint Centres of Rotation

Approximate joint centres of rotation for the sarco-urostylic (SU), sacroiliac (SI), hip, knee, and ankle joints were assigned using the point tool in Rhinoceros 3D as follows. The SU joint centre was placed at the midline position where the urostyle articulated with the sacrum. Since the IS X and Y joints are bilaterally symmetrical paired joints, a single centre of rotation was defined for both joints as the point equidistant between them. The hip joint centre of rotation was calculated by manually fitting a sphere to the head of the femur (using the STL mesh as an anatomical guide), representing the femoral head, and assigning joint centre of rotation as the centre of the sphere. After Kargo and Rome (Kargo and Rome, 2002), the knee joint centre was calculated as a “rolling joint” allowing the tibia-fibula to slide along the rounded articular surface of the femur. The parameters specifying the rolling action were taken from previously published anatomical data (Kargo and Rome, 2002). The joint centre of rotation for the ankle joint was calculated by drawing a line through the long axis of each limb segment and assigning joint centre of rotation as the intersection of the two long axis lines.

### Segment Local Reference Frames

**FIGURE 2:**
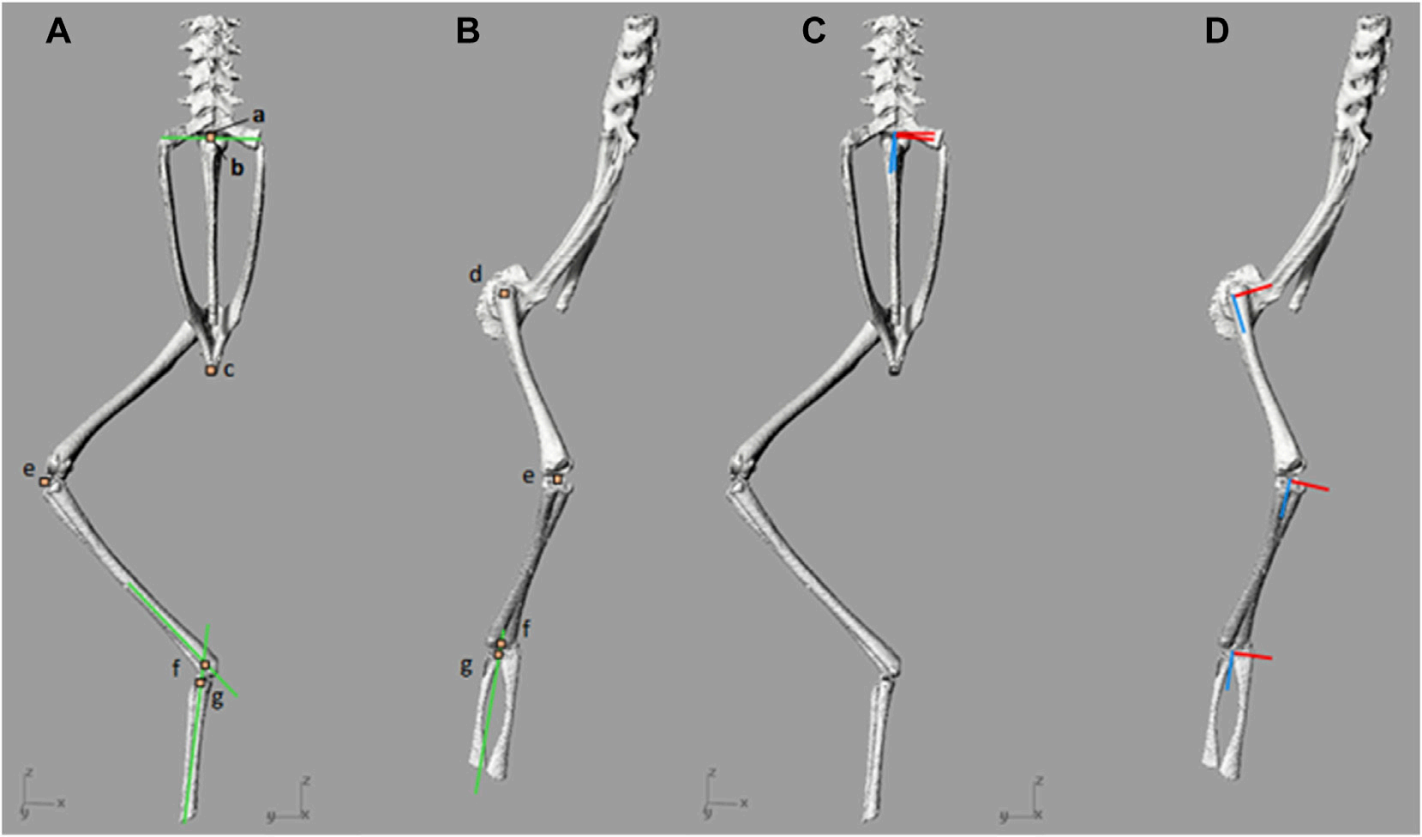
**(A,B)**—Local origins and approximate joint centres of rotation, and **(C,D)**—joint local axes. Joint origins and approximate centres of rotation are denoted by orange squares which have been enlarged for visualisation. Marker a) includes the local origin for the spine and the urostyle, as well as the sacro-urostylic joint centre of rotation. Marker b) represents the sacroiliac joint centre of rotation. Marker c) represents the pelvic local origin. Marker d) includes the femur local origin and the hip joint centre of rotation. Marker e) includes the tibiofibula local origin and the knee joint centre of rotation. Marker f) represents the ankle joint centre of rotation and marker g) represents the tarsal segment local origin. The green lines present in **(A)** and **(B)** are the reference lines used to find SI and ankle joint centres of rotation. In **(C)** and **(D)**, the *X* axes are red and *Z* axes are blue.

To inform model output data (kinematics and moment arms) definitions, it was necessary to first define local reference frames. In order to define a local reference frame, three points were required; a local origin, an axis vector, and a corresponding point; together these three points were used to define a plane. A local origin for each skeletal segment was created corresponding to repeatable landmarks. For example, the hip, knee, and urostyle local origins were assigned to coincide with the joint centres of rotation for the hip joint, knee joint and SU joint, respectively. The local origin of the pelvis was assigned as the distal most point along the midline, and for the tarsals the local origin was assigned as the centre-most point at the proximal end of the bones, where they fuse. A second point, the axis vector point, was then placed in line with the local origin to generate an axis vector. To ensure the axis vector lined up with meaningful anatomical rotations (i.e., flexion/extension, abduction/adduction, and cranial/caudal long axis rotation), the axis vector point was placed such that the resulting vector fell in line with the long axis of the bone, or created an orthogonal line with the long axis of the bone. A third arbitrary corresponding point was then placed to generate a plane between the local origin point, and the axis vector point. Using those three points, a custom MATLAB function (R2016b, The MathWorks, Inc., Natick, Massachusetts, United States) automatically generated the set of orthogonal frame axes. Through the positioning of the points, local reference frames for all segments were assigned such that positive Z was aligned along the long axis of the segment. For the urostyle, spine, and pelvis positive *Y* axis was aligned straight up with respect to the local origin, and positive *X* axis to the right of the segment origin. For the hindlimb segments, *X* and *Y* axes were based on surface features on the articular surfaces of the bone; X across the adduction/abduction plane of the segment, and Y across the flexion/extension plane. All local origins, local axes, and approximate joint centres of rotation are shown in Figure 2.

### Muscle Attachment and *Via* Sites

Muscle attachment sites were assigned with the use of the 3D meshes of the individual muscles by placing landmark points, using the point tool in Rhinoceros 3D, at their origination and insertion sites. For some muscles, it was sufficient to use a single point each to represent the muscle origin and insertion, whereas those muscles with large attachment areas required multiple points to define origination and insertion. In some instances, muscles insert into other muscle bellies [gluteus (GL) into cruralis (CR)] or into shared aponeuroses (CR). In those cases, both muscles shared the insertion point of the larger muscle or the aponeurosis (Hutchinson, personal communications).

For those muscles that exhibited particularly complex geometries [e.g., iliacus externus (IE)], additional *via* sites were used. A *via* site is an additional point placed along the muscle pathway that the MuJoCo muscle tendon unit (MTU) must run through. Note that the term muscle tendon unit in this circumstance is uniform in properties, therefore the entire path is assumed to be muscle as opposed to both muscle and tendon. Since the MTU in MuJoCo represents the shortest distance between the two attachment sites, *via* sites can be used to constrain the muscle pathway. The IE muscle, for example, runs the length of the lateral surface of the ilium and attaches to the proximal femur. For this muscle, attachment sites were placed at the origin (anterior ilium) and at the insertion point on the proximal femur, a *via* site was then also placed on the posterior ilium such that the MTU would run from the origin, along the lateral surface of the ilium, through the *via* site, and to the origin. Without the use of the *via* sites, the MTU would not be constrained to the lateral surface of the ilium.

Finally, since the contrast enhancing agent used was unable to resolve tendinous tissue, the muscle meshes of those muscles with tendinous insertions did not make contact with the bone meshes. Consequently, placing insertion points for these muscles required estimation based on the muscle belly pathway and anatomical knowledge gained from the traditional dissection of other animals of the same species (Collings and Richards, 2019). In particularly challenging cases, where the tendon was relatively long, the line tool in Rhinoceros 3D was used to draw a line through the muscle belly midline to best represent muscle line of action, where this line intersected the bone mesh the muscle was estimated to insert. All muscle attachment site points were placed on to the skeletal meshes in the global reference frame. The point coordinates would later be automatically transformed from the global reference frame to the local reference frame of the segment they were situated on when loaded into the MATLAB autofill function (described below).

### Wrapping Geometries and Side Sites

In MuJoCo MTUs are modelled as the straight line between attachment sites. However, even with the use of *via* sites, modelling a MTU as a straight line often resulted in cases where the MTU would ‘clip’ through the bone meshes or other MTUs. Therefore, to avoid such collisions and more accurately represent the natural smooth curved muscle pathways, wrapping geometries were implemented. MuJoCo permits each MTU to wrap around a single wrapping geometry between any two sites, ignoring all other geometries. When wrapping over geometries between sites the MTU length is then the shortest arc length over the wrapping geometry. The muscle 3D surface STL meshes were used to inform size, position, and orientation of the wrapping geometries, which were either spheres or cylinders of infinite length, created using the Sphere and Cylinder tools in Rhinoceros 3D, respectively. Specific side sites for each wrapping geometry were also placed. These side sides were required to specify the hemisphere or semicircle of the wrapping geometry that the MTU was permitted to wrap over. In other words, the side sites acted to constrain the MTU pathway to one cross sectional plane of the wrapping geometries throughout a simulation. Without side sites, the MTUs are free to wrap over whichever surface of the wrapping geometry allows the shortest path length.

### MuJoCo Model File Generation

All model information was exported from Rhinoceros 3D. The object information (point coordinates and wrapping geometry data) was exported as a text file, whereas the skeletal segment meshes were exported as binary STL files. To minimise computation time, any mesh that contained more than 200,000 polygons first required a reduction in mesh size which was conducted using the ReduceMesh tool.

Since MuJoCo requires model information in XML format, an XML template was created and subsequently populated with all model object information from the Rhinoceros 3D object information text file (e.g., coordinates of origin/insertion/*via* sites as well as of joint centres). This was achieved using a custom autofill function written in MATLAB. Additional information was input directly into the XML file, including joint type. The hip and ankle joints were defined as a ball joint, the left and right IS joints, and the SU joint were defined as hinge joints, and the knee joint was defined as a rolling joint (a hinge joint with some fore-aft translation, see above). The ball joint degrees of freedom allowed adduction/abduction about the *X* axis, flexion/extension about the *Y* axis, and long axis rotation about the *Z* axis. The IS hinge joint was modelled with two degrees of freedom by placing two hinge joints at the same centre of rotation. One hinge joint permitted dorsal/ventral rotation about X, and the second overlying hinge joint allowed lateral rotation about Y. The same approach was taken for modelling the SU hinge joint, a bicondylar joint modelled with a single centre of rotation between the two condyles. This approach permitted the joints to be modelled as hinges operating in both the sagittal plane and the frontal plane.

**FIGURE 3:**
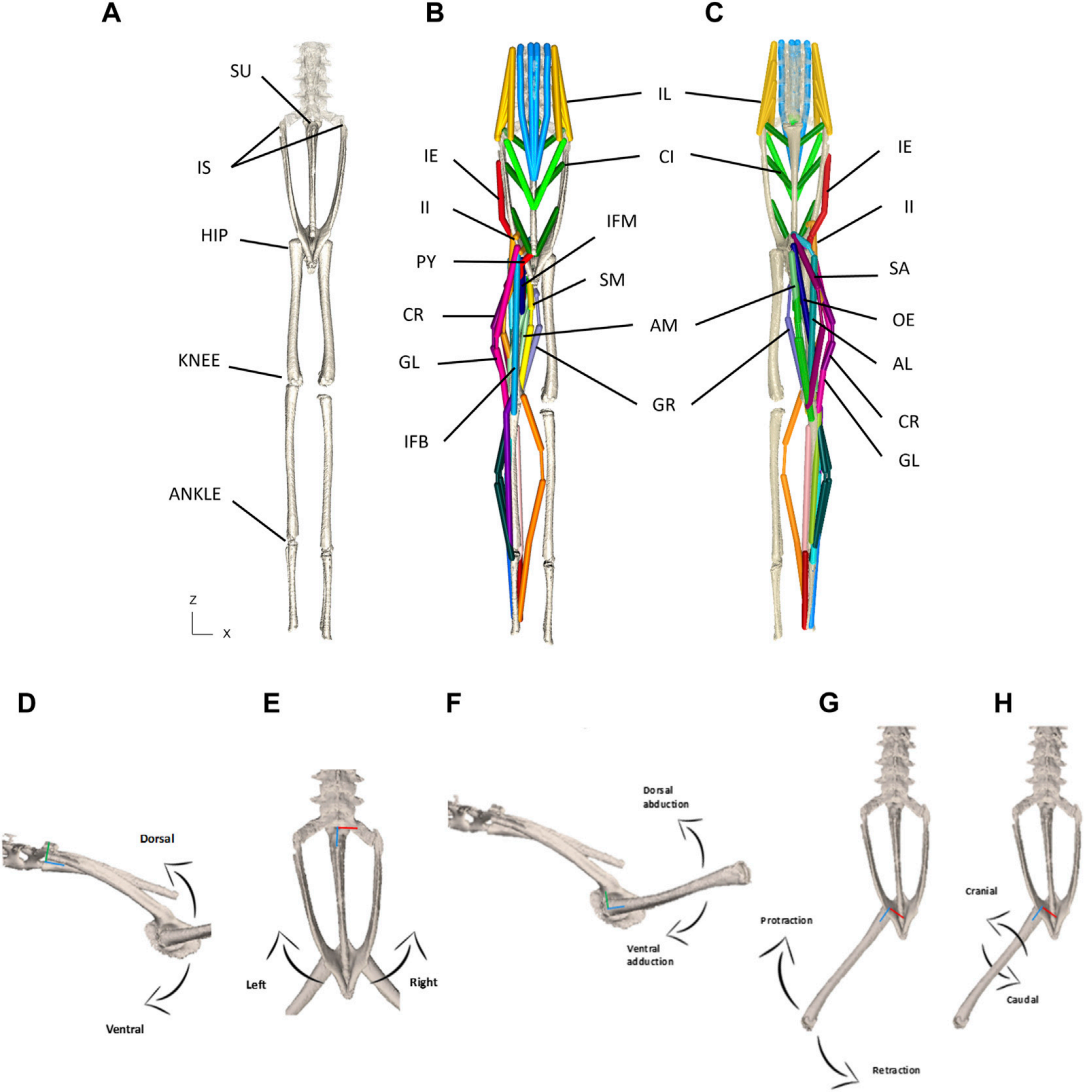
**(A–C)** Model puppet shown in ‘zero’ pose from dorsal **(A,B)** and ventral **(C)** view. Skeletal elements only shown in **(A)** and all MTUs shown in **(B,C)**. Note that shank muscles are included but not reported on in this paper. Please See Table 1 for muscle abbreviations. **(D–H)** Schematic for the visualisation of permitted rotational degrees of freedom for the pelvis at the SI joint **(D,E)** and the femur at the hip joint **(F–H)**. **(D)**—lateral view depicting dorso-ventral rotation about the *X* axis. **(E)**—Dorsal view depicting lateral rotation about the *Y* axis. **(F)**—lateral view depicting dorso-vetral abduction/adduction about the *X* axis. **(G)**—Dorsal view depicting protraction/retraction about the *Y* axis. **(H)**—Dorsal view depicting cranial/caudal long axis rotation about the *Z* axis. The *X* axes are red, *Y* axes are green, and *Z* axes are blue.

Once populated the XML file acted essentially as the model ‘puppet’ (Figures 3A–C). In order to animate the model, an input file containing joint kinematic information was required. The XML files and other supporting files as well as code will be shared in Github repository upon acceptance.

### Export of Limb Segment Angles From Experimental Kinematics

Joint angle data was calculated from previously recorded skin marker-based experimental kinematics coordinate data for walking (Collings et al., 2019) using a custom MATLAB script. Limb segment orientations relative to the body were calculated and expressed as quaternions.

As described above, we used ball joints to characterise various joints. Although a ball joint is characterised by three rotational degrees of freedom, our calculations directly account for only two rotations (flexion/extension and abduction/adduction); the third rotation, cranial/caudal long axis rotation is only inferred by a set of post-hoc calculations, as explained below.

A body plane was defined using the paired shoulder girdle and ilia markers, all hindlimb and pelvic marker motion were then calculated with respect to that plane. Joint segments were expressed as vectors and joint angles were calculated as the Arc Cosine of the dot product of the normalised vectors defining the joint (Eq. 1). The joint axis of rotation was then calculated as the cross product of the normalised joint segment vectors (Eq. 2).

JointAngle = Arccosine (Normalised vector1⋅Normalised vector2) (1)

JointAxis = Normalised vector1∗Normalised vector2 (2)

Where **vector1** is the proximal segment defining a joint and **vector2** is the distal segment. The joint angles were transformed into quaternions using the following formula (Eq. 3):

Quaternion= Cos(θ/2), XSin(θ/2), YSin(θ/2), ZSin(θ/2) (3)

Where θ is Joint Angle and *X, Y, Z* are the X, Y, Z coordinates of the normalised axis of rotation.

Quaternions offer an alternative method of describing the orientation of an object in space. They are formatted as four numbers, one scalar unit along with a three-dimensional vector, as shown in Eq. 3. Quaternions were used in this instance to express the orientation of the joint segments throughout the stride cycle, a computationally quicker and easier method than combining rotation matrices for X, Y, Z coordinates. The joint quaternions were filtered using a Reverse Butterworth filter with a cut off frequency of 30 Hz. To animate the joint motion of the right hindlimb during walking, the left hindlimb kinematics were played 180° out of phase.

Note that segment movements can occur in any plane. For example, if two limb segments are in the horizontal plane, the resulting rotation axis points vertically. Conversely, if the two segments are in the vertical plane, the resulting rotation axis is horizontal. In reality, during walking, frogs move their limb segments simultaneously in the horizontal and vertical planes (Collings et al., 2019), thus the rotation axes point diagonally to reflect a mixture of flexion/extension and abduction/adduction (Richards, 2019). Hence, Eqs 1–3 allow us to capture two degrees of rotational freedom (flexion/extension; abduction/adduction). However, due to our non-invasive approach, kinematic data were collected using external marker points placed on approximate joint centres of rotation; each limb segment position was defined only by proximal and distal joints. Therefore, long axis rotation of limb segments could not be resolved from our experimental setup. To estimate long axis rotation, we applied some assumptions and performed a series of post-hoc calculations as follows.

Since kinematic data were collected using external marker points placed on approximate joint centres of rotation, each limb segment position was defined by two marker points (the proximal and distal joints). Long axis rotation of limb segments therefore could not be resolved from our experimental setup. However, using some underlying assumptions, it was possible to estimate long axis rotation. We assumed that the femur, tibiofibula, and tarsal segments would be aligned such that deviation from the flexion/extension axis is minimised. Given that each local *Y* axis corresponds to the flexion/extension plane of the limb segment, it was possible to implement a series of rotations to align limb segment *Y* axes. Firstly, the femur was rotated about its local *Z* axis, such that the *Y* axis of the femur aligned with the cross product between the *Z* axis of the femur and the *Z* axis of the tibiofibula. Secondly, the tarsal segment was rotated about its *Z* axes such that the *Y* axis of the tarsals aligned with the cross product between the *Z* axis of the tarsals and the *Z* axis of the tibiofibula. Finally, the tibiofibula was rotated about its *Z* axis so that the *Y* axis of the tibiofibula was aligned halfway between the *Y* axes of both the femur and tarsals. In other words, the angle between the *Y* axes of the tibiofibula and the femur was equal to the angle between the *Y* axes of the tibiofibula and the tarsals.

### Model Simulations in MuJoCo

Simulations were run in MuJoCo using the XML model and joint angle inputs. Two sets of simulations were run to elucidate 1) the impact of femoral and pelvic dorsoventral angle, and 2) the impact of pelvic lateral rotation on hindlimb moment arms during walking; these are referred to as the hypothetical and the walking sequence simulations, respectively.

The hypothetical simulations are a highly simplified set of “numerical experiments” intended to systematically explore how pelvis and femur ranges of motion influence muscle moment arms. They were run using a set of hypothetical joint input angles. The pelvis was set to rotate laterally +/−8° (Collings et al., 2019) across the midline about the *Y* axis, starting from the right rotating past midline to the left and back to the right in a sine wave pattern. This was classed as one lateral pelvic rotation sequence. The femur was then positioned at 4 different static protraction angles in the horizontal plane (10, 45, 90, and 135°) to mimic key positions in a walking stride from full retraction to full protraction. One full pelvic rotation sequence was recorded per femur angle. For example, for the 10-degree condition, the femur is fixed at 10° (nearly fully protracted) whilst the pelvis rotates laterally from right to left then back. During these simulations the pelvis was held in dorsoventral flexion of 45° and the femur was held static at each respective angle such that the only time-varying rotation was lateral rotation of the pelvis about the IS joints. This set of simulations was then repeated while the pelvis was held in dorsoventral flexion of 0, 22 and 45° (Table 2; see SI for movies).

**TABLE 2:** List of simulation parameters, input data, and output data for all hypothetical and walking sequence simulations run in this study. MTU (muscle tendon unit), IS (Iliosacral joint).

Simulation Name	Simulation Type (SI Movie Number)	Simulation Parameters pelvis	Simulation Parameters femur	Inputs	Outputs
HYP_01	Hypothetical (1 and 2)	Pelvis laterally rotating	Left femur held at 10°	Pelvic lateral rotation angle as Sin wave fluctuating+/-8° about midline	Moment arm for all hindlimb and axial MTUs
		IS joint fully extended			
HYP_02	Hypothetical (3 and 4)	Pelvis laterally rotating	Left femur held at 45°		
		IS joint fully extended			
HYP_03	Hypothetical (5 and 6)	Pelvis laterally rotating	Left femur held at 90°		
		IS joint fully extended			
HYP_04	Hypothetical (7 and 8)	Pelvis laterally rotating	Left femur held at 135°		
		IS joint fully extended			
HYP_05	Hypothetical (9 and 10)	Pelvis laterally rotating	Left femur held at 10°		
		IS joint flexed ventrally to 22°			
HYP_06	Hypothetical (11 and 12)	Pelvis laterally rotating	Left femur held at 10°		
		IS joint flexed ventrally to 45°			
RUN_ROT	Walking sequence (13 and 14)	No modification	No modification	Experimental kinematics from exemplar trial for full stride cycle	
RUN_FIX	Walking sequence (15 and 16)	Pelvic lateral rotation fixed	No modification		

The walking sequence simulations were run using joint angle inputs from a representative walking trial collected experimentally (Collings et al., 2019). The walking simulations ran from stance phase-stance phase through one full stride cycle. This simulation was repeated twice, firstly with no modification to the experimental kinematics and secondly where pelvic lateral rotation at the IS joints was fixed (as per Collings et al., 2019; see SI for movies).

Each simulation output the moment arm of each relevant MTU in each frame of locomotion.

Table 2 provides a list of the simulation inputs, parameters, and outputs. Animations of all conditions can be found in the Supplementary Information with movie names corresponding to the simulation names.

### Muscle Moment Arm Output Data

MuJoCo resolved the moment arms of each individual MTU into the different components depending on the joint degrees of freedom. In this instance, moment arms represent the ratio of input force to output torque about each axis. The torque output of a given muscle is therefore proportional to the input force and the moment arm. A large moment arm permits the muscle to generate a proportionally larger torque per given input force, whereas a small moment arm would require the given muscle to input a proportionally larger force to maintain a given output torque.

Moment arms can also be thought of as distances, whereby the output torque is equal to the input force multiplied by the perpendicular distance from the pivot. Moment arm distance (*r*) in 3D can be calculated from the moment arm outputs of MuJoCo using the following equation (Eq. 4).

$$r=\;\sqrt{X^{2}+Y^{2}+Z^{2}}$$ (4)

Where *r* is the perpendicular distance between the joint centre of rotation and muscle line of action, X, Y, and Z are the muscle force to joint torque ratios about the X, Y, and *Z* axes, respectively.

Since the IS joint was modelled using two hinge joints sharing the same centre of rotation, the pelvis was free to move dorsoventrally (about X) and laterally (about Y) (Figures 3D,E), and thus each MTU acting upon this joint had a moment arm about X and a moment arm about Y. For the remaining hindlimb joints, each MTU acting on those joints had three moment arm values calculated: X, Y, and Z corresponding to adduction/abduction (Add/Abd), protraction/retraction (or flexion/extension, Flex/Ex), and long axis rotation (LAR), respectively, (Figures 3F–H). Moment arm analysis was not conducted about the SU joint in this study since there is little evidence to suggest motion at this joint is significant (Emerson, 1979; Emerson and De Jongh 1980).

Since the hip joint was modelled as a ball joint, the hindlimb MTUs spanning this joint have moment arm values about three axes corresponding to protraction/retraction (rotation about Y), abduction/adduction (rotation about X), and cranial/caudal long axis rotation (rotation about Z). In the protraction/retraction plane, MTUs with positive moment arm values generate hip flexion (i.e., femur protraction) whereas those with negative moment arm values are associated with hip extension (i.e., femur retraction). In the abduction/adduction plane, MTUs with positive moment arm values generate hip abduction (i.e., raise the femur dorsally) whereas those with negative moment arm values are associated with hip adduction (i.e., lower the femur ventrally). Finally, for long axis rotation, MTUs with positive moment arm values generate caudal rotation (i.e., roll the femur clockwise caudally) whereas those with negative moment arm values are associated with cranial rotation (i.e., roll the femur anti-clockwise cranially).

A list of muscles including *via* sites are shown in Table 1 and the specific *via* site information is documented in the model XML file. For all muscles, including those with one or more *via* sites, MuJoCo’s kinematics pipeline computes the minimum length path between origin and insertion (while also passing through *via* sites). Moment arms are subsequently calculated by computing the following (Eq. 5):

Moment arm vector = gradient[L(q)] (5)

Where L (q) is the vector of muscle lengths as a function of joint angle. Hence, the correspondence between muscle length changes and joint angle changes is used to solve for muscle moment arms (see MuJoCo documentation; MuJoCo.org).

### Data Analysis

All data from simulations were exported into Mathematica (Wolfram, Hanborough, United Kingdom) for further analysis. Although the current *P. maculatus* model contains 48 MTU’s, we analysed the functions of sixteen muscles that act primarily on the spine, pelvis and upper limb.

## Results

### Hypothetical Simulations: Axial Muscles

**FIGURE 4:**
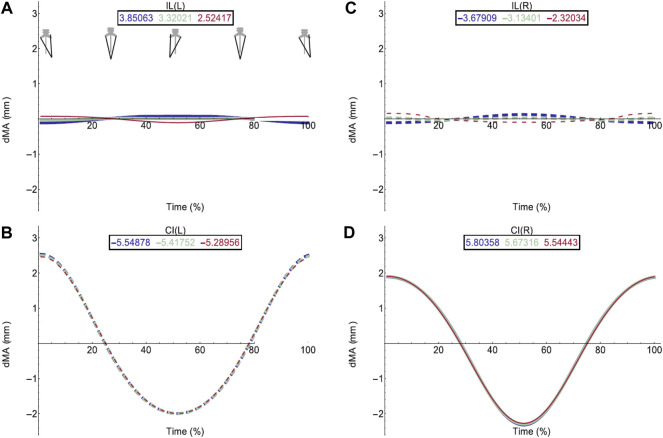
Effect of pelvic rotation on axial muscle moment arms. Changes in moment arm (dMA) versus time are shown for **(A)** the left Iliolumbaris, IL (L), **(B)** left coccygeoiliacus, CI (L), **(C)** right Iliolumbaris, IL (R), **(D)** right coccygeoiliacus, CI (R). Schematic icons indicate the position of the pelvis moving sinusoidally from right (time 0%) to centre (25%) to left (50%) to centre (75%) to right (100%). For each muscle, three hypothetical conditions were run: dorso-ventral iliosacral joint in the extended position (blue), half-flexed (light green) and fully flexed (red). For all conditions, the femur is held at 10° whilst all other joints are held at zero degrees. Traces are shown as changes relative to the mean moment arm (see Methods) such that positive versus negative values indicate deflection above versus below the mean. Boxed values show the mean moment arm value for each condition with colours corresponding to the respective ΔMA plots. Solid versus dashed lines represent positive moment arms (flexion—lateral rotation to the left) versus negative (extension—lateral rotation to the right) such that a change from solid to dashed indicates a change in muscle function. Moment arms for abduction/adduction and long-axis rotation are in SI.

**FIGURE 5:**
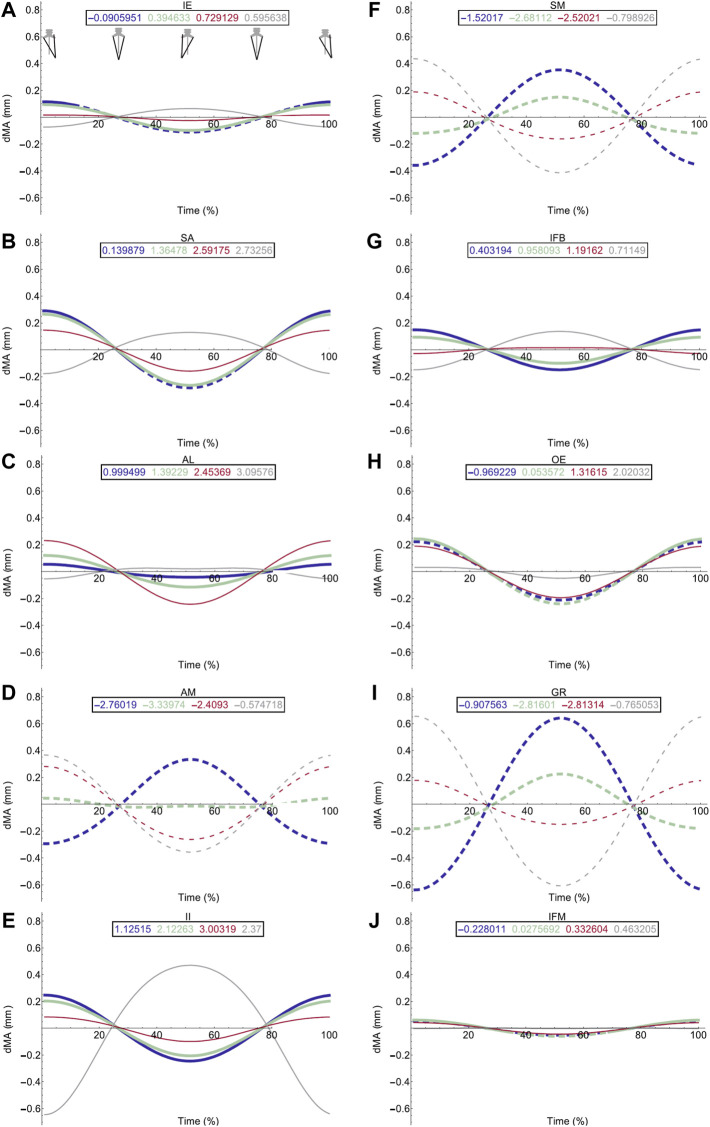
Effect of pelvic rotation on femoral protractor and retractor muscle moment arms. Changes in flexion/extension moment arm (dMA) versus time are shown for protractor muscles **(A–E)**; Iliacus externus, IE, **(A)**, sartorius, SA **(B)**, adductor longus, AL, **(C)**, adductor magnus, AM, **(D)**, Iliacus internus, II **(E)** and retractors **(F–J)**; semimembranosus, SM **(F)**, iliofibularis, IFB **(G)**, obturator externus, OE **(H)**, gracilis minor and major, GR **(I)**, iliofemoralis, IFM **(J)**. For each muscle, four hypothetical conditions were run: femur held at 10° (blue), 45° (light green), 90° (red) and 135° (grey). Note that for these hypothetical conditions, 0° is defined as fully retracted as seen in the null pose (Figure 2 **(E,F)** such that 10° is near full femur retraction and 135 is near fully protracted. Solid versus dashed lines represent positive moment arms (flexion—femur protraction) versus negative (extension—femur retraction) such that a change from solid to dashed indicates a change in muscle function.

**FIGURE 6:**
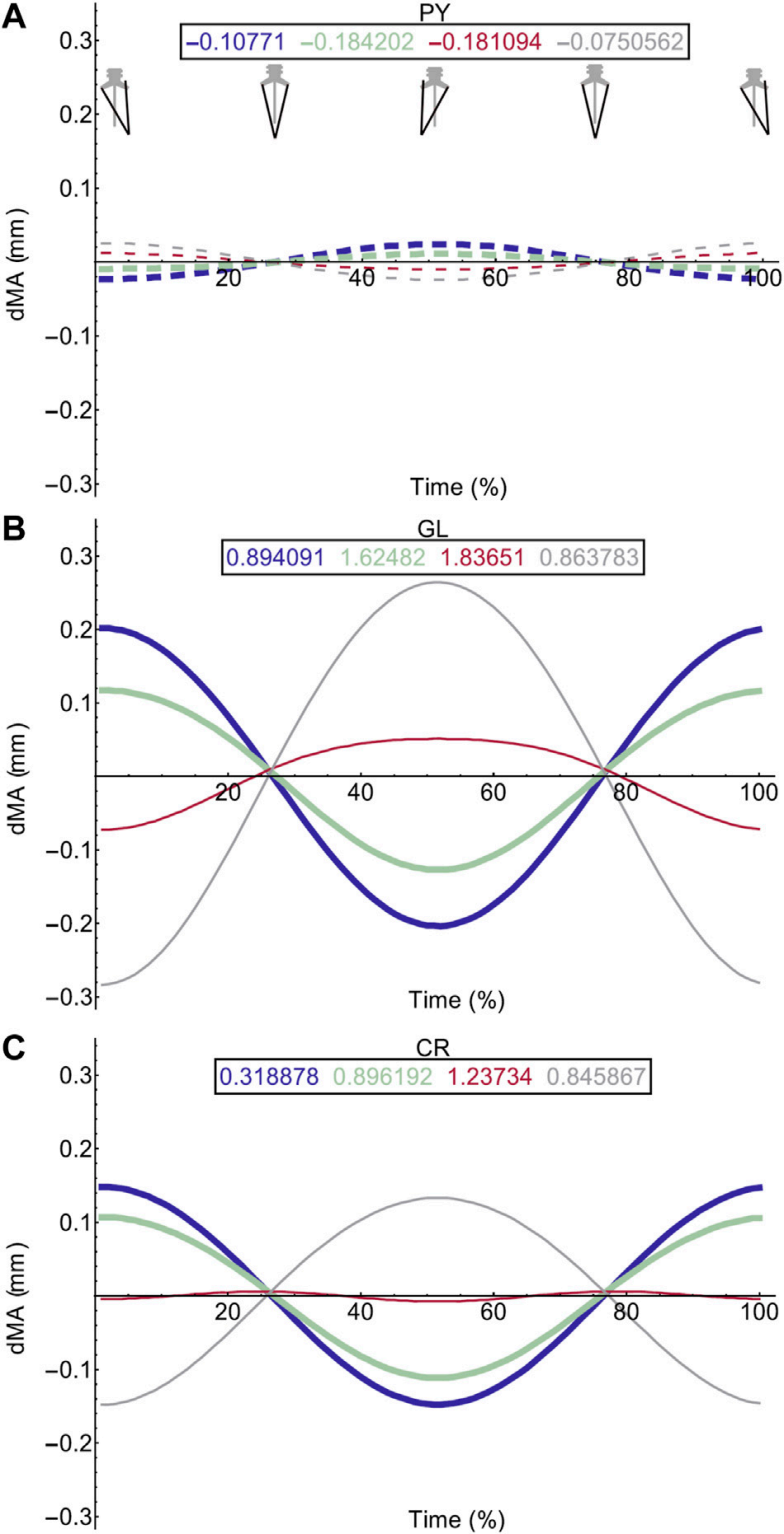
Effect of pelvic rotation on miscellaneous muscle moment arms. Changes in flexion/extension moment arm (dMA) versus time are shown for pyriformis, PY **(A)**, gluteus maximus, GL **(B)**, cruralis, CR **(C)**. See Figure 7 caption for further details. Solid versus dashed lines represent positive moment arms (flexion—femur protraction) versus negative (extension—femur retraction) such that a change from solid to dashed indicates a change in muscle function.

Moment arm plots for all muscles are shown in Figures 4–6. Tables 3, 4 describe the qualitative influence of all tested factors for each MTU moment arm for the axial and hindlimb muscles, respectively.

**TABLE 3:** Results of hypothetical simulations HYP_01, HYP_05, and HYP_06 describing the influence of pelvic lateral rotation and pelvic dorsoventral rotation on the lateral rotation moment arms of the axial muscles.

Predicted Functional Group	MTU	Influence of pelvic Lateral Rotation	Influence of pelvic dorsoventral Rotation
Axial muscles	CI	Change in moment magnitude, left and right inverted	Change in moment magnitude
	IL	Change in moment magnitude but pattern dependent on dorsoventral angle of pelvis	Change in moment magnitude and varying influence on pelvic lateral rotation

**TABLE 4:** Results of hypothetical simulations HYP_01-HYP_06 describing the influence of pelvic lateral rotation and femur angle in the flexion/extension plane on the flexion/extension (FE), long axis rotation (LAR), and abduction/adduction (AA) moment arms of the hindlimb muscles.

Predicted Functional Group	MTU	Impact of pelvic Lateral Rotation	Impact of femur Angle (Flexion/extension)
Protractors	IE	Change in moment magnitude (increase or decrease dependent femur angle)	Change in moment magnitude and function in FE and LAR
Retractors	SM	Shallow change in FE magnitude only	Change in FE and LAR magnitudes. Influence of pelvic lateral rotation dampened in mid femoral angles
	IFB	Slight impact in FE magnitude only (increase or decrease dependent femur angle)	Change in FE magnitude
	OE	Shallow change in FE magnitude only	Decrease in FE moment magnitude and switch of function from protractor to retractor in full extension
Protraction and adduction	SA	Shallow impact on FE moment magnitudes and AA moment (only when femur is fully retracted)	Influences the magnitude of all moment arms. Changes in sign/function seen in the AA moment when femur at full retraction angle
	AL	Only shallow magnitude changes in flexion/extension moment arms when femur is at 90°	Change in FE and LAR magnitudes with more retracted femoral angle
	AM	Change in FE moment magnitude	Small change in FE and LAR moment magnitude and change in FE function
Retraction and adduction	GR	Change in FE and LAR moment magnitudes	Small change in LAR moment magnitudes when femur in retracted position
			Variable FE moments dependant on femur angle. Very little impact of AA moments
	IFM	Very shallow change in FE moment magnitude only	Small change in FE magnitude and change in FE sign/function from positive to negative at full femoral retraction angle
Protraction and abduction	II	Change in moment magnitudes but dependant on femur angle	Change in all magnitudes and change in FE sign/function. Some impact on long axis rotation and abduction/adduction moment arm magnitudes. Larger influence on flexion/extension moment arm magnitudes
Abduction	PY	None	Very small changes in FE and LAR magnitude
Knee extensor	CR/GL	Impact on FE moment magnitudes (except when femur at 90°)	Small influence on magnitude in LAR and AA. Larger influence on FE, strongest at 90° and weakest at full retraction

The hypothetical simulations demonstrated that the left IL and right CI had positive moment arms (left lateral rotation) and the right IL and left CI had negative moment arms (right lateral rotation) throughout pelvic rotation. Both axial muscle moment arms were impacted by pelvic lateral and dorsoventral rotation to differing extents. While the function of the CI muscles remained the same throughout the pelvic rotation cycle, the moment arm outputs fluctuated in a sinusoidal wave approximately+/−2 mm about the mean indicating pelvic lateral rotation impacted moment arm magnitudes (Figures 4B,D). This impact on the CI moment arm remained the same despite dorsoventral rotation. The mean moment arms however were approximately 0.3 mm higher when the pelvis was extended versus flexed.

Similar to the CI, function of the IL muscles remained the same throughout the pelvic lateral rotation cycle. While there was a very slight fluctuation about the mean, this change in magnitude with lateral rotation was barely measurable, indicating that pelvic lateral rotation had a far smaller impact on IL muscles compared with CI muscles. However, the IL muscles showed more variation in average moment arm in response to dorsoventral rotation ranging from 3.85 mm (extended)—2.5 mm (full flexed) and the shallow wave form was inverted in the fully flexed simulation (Figures 4A,C).

### Hypothetical Simulations: Femoral Muscles

Pelvic lateral rotation influenced moment arm magnitudes for muscles crossing the hip (Table 4). Generally, the impact of pelvic lateral rotation (i.e., peak-to-peak amplitude of the waveform) was more pronounced in the protractor muscles (IE, SA, AL, AM, II; Figure 5). The retractor group muscles presented shallower wave forms and magnitude changes were most dramatic in flexion/extension (Flex/Ex) components. In all muscles, the adduction/abduction (Add/Abd) moments were less dependent on lateral rotation than Flex/Ex and long axis rotation (LAR). Hence, only the Flex/Ex moments will be discussed here, and LAR and Add/Abd outputs are shown in SI. The IE and SA were the only MTUs where a change in sign (or ‘function’) of the moment was observed in response to pelvic lateral rotation; this was only seen when the femur was fully extended (10°). Sometimes, the strength of the impact of lateral rotation on the moment arm magnitudes depended also on the femur angle, for example in OE (Figure 4H), where pelvic lateral rotation only influenced moment arm magnitudes when the femur was positioned at 0, 45, and 90° (Figure 4H). In other instances, the position of the femur determined whether the waveform flipped (i.e., whether it increased as the pelvis rotated to the left or decreased as it rotated). A clear example of this “flipping” can be seen in the II MTU (Figure 4E) where lateral rotation creates a sine wave output at 0, 45, and 90°, but a cosine wave at 135°. In functional terms, for the II when the femur is fully flexed (135°), pelvic lateral rotation increases II flexion moment arm as the pelvis rotates to the left, whereas when passed 90° pelvic lateral rotation decreases II flexion moment arm as the pelvis rotates to the left.

Femur angle had a pronounced impact on MTU moment arms, influencing both magnitude (all MTUs except IFM) and moment arm sign (‘function’) (IFM and OE). For some MTUs (mostly protractors) moment arm magnitudes became progressively stronger as the femur flexed, for example AL (Figure 4C) where the mean flexion moment arm increased from approximately 1 mm at 0°, to 3.1 mm at 135°. In others (mostly retractors), the moment arms became weaker with femur flexion, for example AM (Figure 4D) where the mean extension moment arm decreased from −3.4 mm at 45° to −0.6 mm at 135°. In some cases, the moment arms were strongest at the mid angles (45 and 90°), but weakest at the extremes, as is true for GR (Figure 4I) and GL (Figure 6B).

### Walking Sequence Simulations: Axial Muscles

**FIGURE 7:**
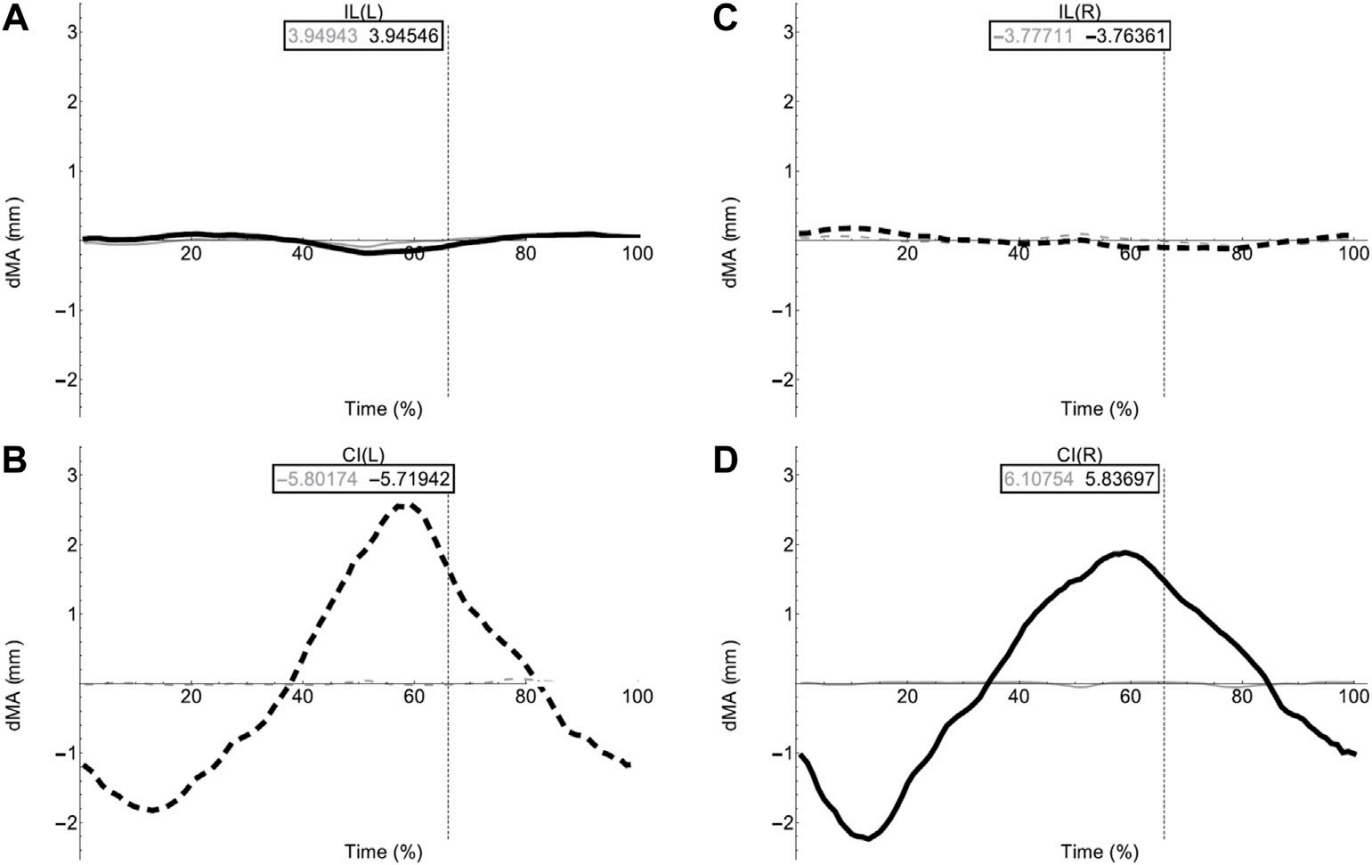
Axial muscle moment arms during walking. Changes in moment arm (dMA) versus time are shown for **(A)** the left Iliolumbaris, IL (L), **(B)** left coccygeoiliacus, CI (L), **(C)** right Iliolumbaris, IL (R), **(D)** right coccygeoiliacus, CI (R) for simulations with a mobile pelvis (natural condition; black) versus a fixed pelvis (simulated condition; grey). See Figure 3 caption for further details. Solid versus dashed lines represent positive moment arms (flexion—femur protraction) versus negative (extension—femur retraction) such that a change from solid to dashed indicates a change in muscle function.

Table 5 provides a summary of function and describes the impact of fixing pelvic lateral rotation during a walking sequence on the axial muscles. Simulation outputs for the axial muscles can be seen in Figure 7.

**TABLE 5:** Results of walking sequence simulations RUN_ROT and RUN_FIX describing the impact of fixing pelvic lateral rotation on the lateral rotation moment arms of the axial muscles during walking.

Predicted Functional Group	MTU	Moment arm	Impact of Fixed pelvic Rotation	Summary of Function
Axial muscles	CI (right and left)	Time varying moments creating a triangular shape waveform through the stride cycle	Elimination of moment arm magnitude fluctuation	Left and right antagonistic pairs generating left and right lateral rotation of pelvis about SI joint
		Both left and right MTU moments arm magnitudes peak at the onset of swing phase but have opposite moments	Comparably weaker moments when each respective muscle is likely to be active	Right CI produces left rotation and Left CI produces right rotation of the pelvis
	IL (right and left)	Time varying moments creating the inverted waveform with respect to the CI	Reduction in moment arm magnitude fluctuation	Left and right antagonistic pairs generating left and right lateral rotation of pelvis about SI joint
		Both left and right MTU moments magnitudes peak during stance phase but have opposite moments	Comparably weaker moments when each respective muscle is likely to be active	Left IL produces left rotation and Right IL produces right rotation of the pelvis

Fixing pelvic lateral rotation had little impact on the mean moment arm values for either the left or right CI and IL muscles. However, the fluctuation in magnitude of the CI moment arm with pelvic lateral rotation was impacted. In Figures 7B,D, the moment arm output for the fixed simulation is a flat line that does not fluctuate about zero whereas the unaltered walking simulation output is a triangular wave form fluctuating approximately+/−2 mm about zero.

### Walking Sequence Simulations: Femoral Muscles

Table 6 provides functional interpretations and descriptions of pelvic lateral rotation impact in the Flex/Ex plane of motion and summarises function for each MTU during walking. Data for the LAR and Add/Abd planes are excluded from this comparative table since moment arm magnitudes were comparatively low and/or were minimally impacted by pelvic lateral rotation (see SI).

**TABLE 6:** Results of walking sequence simulations RUN_ROT and RUN_FIX describing the impact of fixing pelvic lateral rotation on the flexion/extension (FE) moment arms of the hindlimb muscles.

Predicted Functional Group	MTU	FE Plane	Fixed Pelvis	Summary of Function
Protraction	IE	Dist—weak flexor moment becomes extensor moment during limb retraction	Dist - Slight strengthening of extensor moment	Hip flexor
		Prox—flexor moment that weakens throughout stance phase as limb retracts	Prox—Flexor moment weakened during stance phase	
Protraction and adduction	SA	Flexor moment but gets weaker during limb retraction	Weakened flexor moment during stance phase	Hip flexor
	AL	Flexor moment weakens as limb retracts and strengthens through protraction peaking as limb is brought into protracted position ready for stance onset	No significant change	Hip flexor
	AM (crv)	Extensor moment strengthens as limb retracts	Similar pattern and magnitude however moment is slightly weaker during limb retraction and slightly stringer during swing phase	Hip extensor and cranial rotator
		Str—starts weak protractor, towards zero with retraction	AM str—weakens flexor moment and strengthens the extensor moment	
Protraction and abduction	II	Lat and Med—Flexor moment which weakens during stance phase	Lat—weakened flexor moment causing a flip to very weak extensor moment as the limb approaches full retraction	Hip flexor and caudal rotator
			Med—weakened flexor moment	
Retraction	SM	Extensor moment that is weakens as limb protracts in swing phase	Extensor moment is weakened during stance phase	Hip extensor
	IFB	Flexor moment which flips to weak extensor moment as hindlimb approach maximum retraction during stance	Slight strengthening of extensor moment during stance phase	Caudal rotator, weak Hip extensor during stance and Hip flexor during swing
	OE	Flexor moment which weakens throughout stance phase	Weakened flexor moment causing a flip to very weak extensor moment as the limb approaches full retraction	Cranial rotator and Hip flexor
Retraction and adduction	GR	Extensor moment gets weaker as hindlimb retracts in stance phase	Extensor moment weakened significantly during stance phase	Hip extensor
	IFM	Flexor moment which flips to extensor moment as hindlimb approach maximum retraction during stance	Very slight strengthening of extensor moment during stance phase	Caudal rotator, Hip extensor during stance and Hip flexor during swing
Abduction	PY	Extensor moment gets stronger with retraction	No significant change	Hip extensor and caudal rotator
Knee extensor	CR/GL	Flexor moment gets weaker with limb retraction	Weakened flexor moment throughout limb retraction	Hip flexor

### Flexion/Extension Moment Arms During Walking Locomotion

**FIGURE 8:**
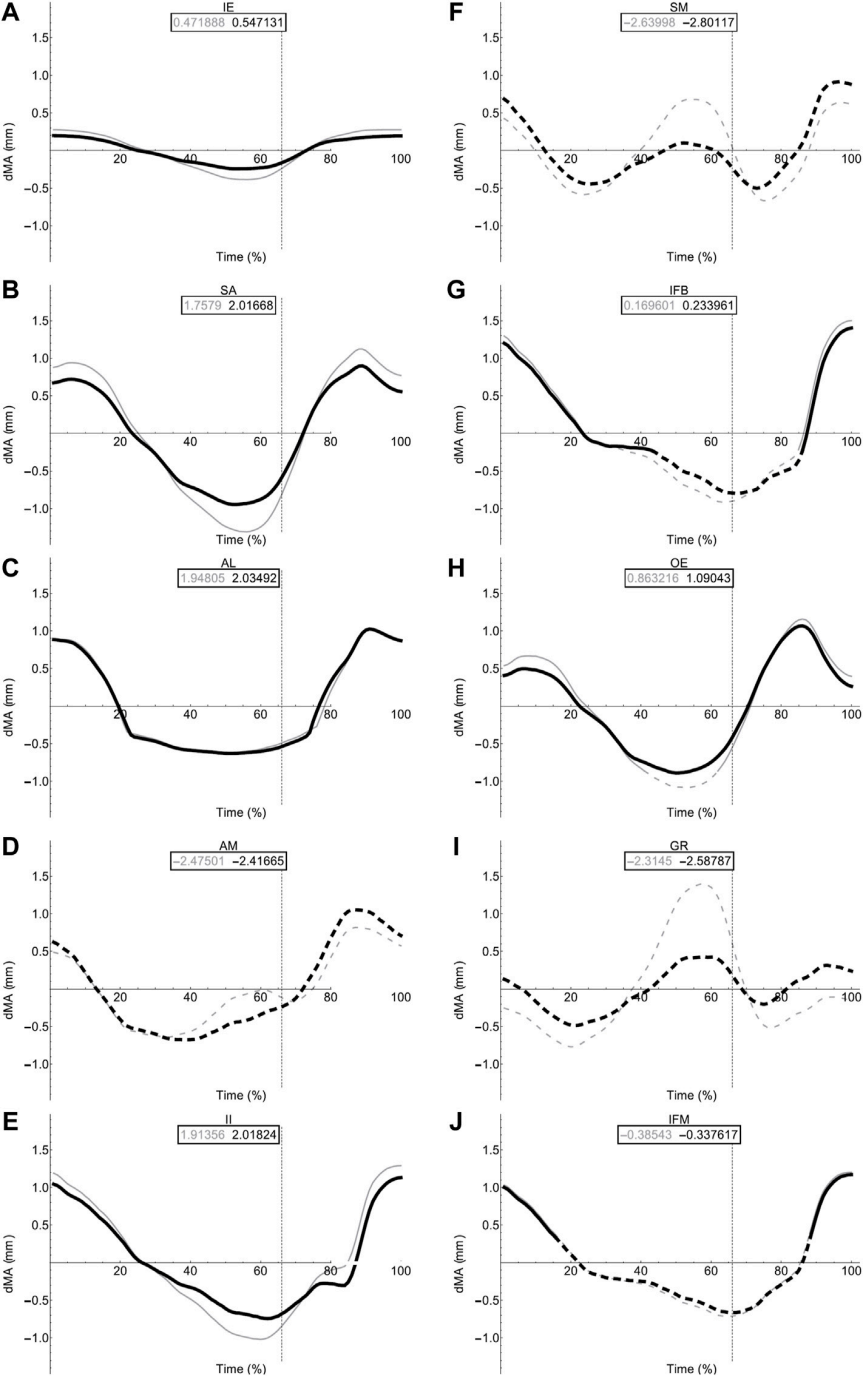
Femoral muscle moment arms during walking. Changes in moment arm (dMA) versus time are shown for protractor muscles **(A–E)**; Iliacus externus, IE, **(A)**, sartorius, SA **(B)**, adductor longus, AL, **(C)**, adductor magnus, AM, **(D)**, Iliacus internus, II **(E)** and retractors **(F–J)**; semimembranosus, SM **(F)**, iliofibularis, IFB **(G)**, obturator externus, OE **(H)**, gracilis minor and major, GR **(I)**, iliofemoralis, IFM **(J)** for simulations with a mobile pelvis (natural condition; black) versus a fixed pelvis (simulated condition; grey). See Figure 7 caption for further details. Solid versus dashed lines represent positive moment arms (flexion—femur protraction) versus negative (extension—femur retraction) such that a change from solid to dashed indicates a change in muscle function.

**FIGURE 9:**
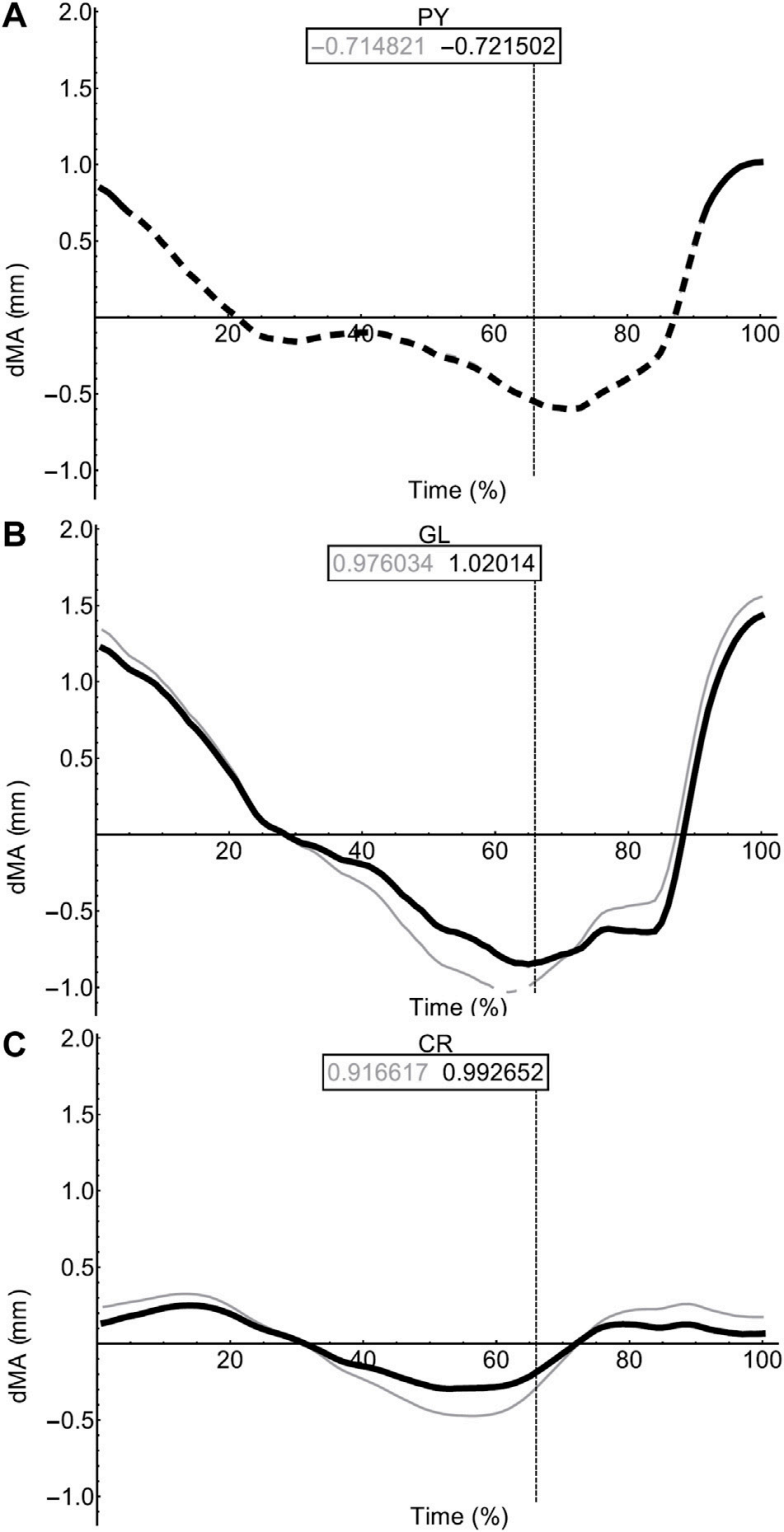
Miscellaneous muscle moment arms during walking. Changes in moment arm (dMA) versus time are shown for pyriformis, PY **(A)**, gluteus maximus, GL **(B)**, cruralis, CR **(C)**. for simulations with a mobile pelvis (natural condition; black) versus a fixed pelvis (simulated condition; grey). See Figure 7 caption for further details. Solid versus dashed lines represent positive moment arms (flexion -—femur protraction) versus negative (extension—femur retraction) such that a change from solid to dashed indicates a change in muscle function.

Flex/Ex moment arm plots for all muscles are shown in Figures 8, 9. For those muscles represented by multiple MTUs, the MTU with the strongest moment arm value is presented.

In contrast to the pelvic MTU moment arms in walking (Figure 7), the hindlimb moment arms did not exhibit the same triangular wave patterns, but instead showed smoother sinusoidal fluctuations in values (Figures 8, 9). The SM, AM, and GR MTUs maintained negative Flex/Ex moment arm values throughout the walking stride suggesting that, at all limb positions, these muscles function as hip extensors (i.e., would retract the femur). Whereas the IE, SA, AL, II, OE, CR/GL maintained positive Flex/Ex moments throughout the stride cycle suggesting these muscles function as hip flexors (i.e., would protract the femur). As the limb unfolded during the stance phase the protractor moment arm of these muscles became progressively weaker until the hip was flexed in the swing phase. As the femur was protracted progressively further the moment arms became progressively stronger, suggesting these muscles are most effective at producing hip flexion once the femur has already begun to protract. The IFB, IFM, and PY muscles fluctuated from positive moments to negative moments throughout the stride cycle, starting as flexors, switching to extensors during stance phase and negative during swing, flipping back to positive as the limb realigns itself for the onset of the next stance phase.

Fixing pelvic lateral rotation impacted the protractor and retractor muscles differently. In terms of mean moment arm value, those MTUs with flexor moment arms (i.e., protractors; including IFB) as well as GR, SM, and PY (which have extensor moments) were ∼0–10% weaker when the pelvis was fixed. However, for the remaining MTUs, AM and IFM, the mean moment arms were ∼3–20% stronger when the pelvis was fixed. There were also differences in the level of fluctuation about the mean value in many of the muscles, where the fixed pelvic outputs showed more exaggerated peaks and troughs compared with the unaltered pelvic simulation outputs. For example, the mean moment arm in the SA MTU in the fixed simulation is lower than is was in the rotating simulation (1.76 versus 2.02 mm; Figure 8B), yet the fixed simulation showed a wider fluctuation about the mean with higher peaks at time 0 and 100% (∼0.8 mm compared with 0.6 mm) as well as lower troughs at time 55% (approaching −1.5 mm compared with just under −1 mm).

### Long Axis Rotation and Abduction/Adduction of the Femur

Most MTUs had lower moment values in LAR compared with values in Flex/Ex (see SI). The II, IFM, IFB, and PY had the strongest caudal rotator moments, whereas the AM, SA, and OE had the strongest cranial rotator moments. Generally, regardless of moment sign, LAR rotators were strongest mid-stride cycle as the limb makes the transition from stance to swing. Fixing pelvic lateral rotation had little effect on moment arm magnitudes in any of the hindlimb muscles (see SI for remaining moment arm components).

## Discussion

The present study combined anatomical data with experimental kinematics to build and animate a 3D musculoskeletal model of the frog *P. maculatus*. Two sets of simulations were used to elucidate the mechanical impacts of pelvic lateral rotation during walking locomotion. The first allowed exploration of the entire integrated system during walking whereas the hypothetical simulation with the fixed pelvis allowed us to isolate the impact of lateral rotation by the pelvis without confounding effects from the other joint motions.

### Axial Muscles Have Greater Leverage in the Walking Configuration

The mechanical function of the pelvis and the axial muscles is not obvious. The 3D geometry and model motion combined with previously published EMG data (Emerson and De Jongh, 1980) indicates that the CI and IL form contralateral pairs where the left CI is active simultaneously with the right IL, and vice versa. Consequently, the *left* CI and *right* IL work together rotating the pelvis to the *right* (the opposite then being true for left lateral rotation). EMG data, however, is unable to resolve how effective those muscles are during their activation cycles and how those actions change with pelvis and limb motion.

Through our simulations, we measured the action of the pelvic muscles during various pelvic rotations (dorsoventral and lateral) and built our understanding of how pelvic motion influences the leverage of the pelvic muscles. In both the hypothetical simulation and the walking sequence simulations the moment arm magnitudes of the axial muscles were time variable in accordance with the hypothetical limb positions and lateral rotation of the pelvis. At full protraction of the left limb (maximum rotation of the pelvis to the left), the right IL and the left CI reached their maximum moment magnitudes. This means that when the right CI and left IL are contracted, they have their weakest moment arm magnitude; and the opposite muscle bellies for both muscles have their largest moment arms while relaxed. Consequently, as the relaxed muscle bellies are activated, they are able to generate a proportionally large amount of torque to swing the pelvis rapidly back in the other direction at the same time as the antagonistic muscle bellies relax and passively lengthen. This allows the pelvis to contribute to limb protraction and retraction during walking while minimising any potential counter-torque effects if contralateral axial muscle activations overlap.

Dorsoventral rotation of the pelvis additionally impacted moment arm magnitudes in the axial muscles, in agreement with Lombard and Abbott’s (1907) excitation study. The impact of increased dorsoventral rotation at the IS joint, however, acted to weaken the lateral rotation moment arm magnitudes for all axial muscles on both sides, except the proximal belly of the CI muscles. When interpreted along with the EMG data (Emerson and De Jongh, 1980) for walking and jumping this weakening is functionally logical. While the pelvis is in a more extended dorsoventral angle (spine and pelvis are more in-line) as is the case in walking, the axial muscles have greater mechanical advantage for generating the left and right lateral rotation seen during walking locomotion. Whereas when adopting a crouched position, with a dorsoventrally flexed pelvis, as observed during jump preparation, the mean moment arm magnitudes for lateral rotation are ∼5% (CI) and ∼35% (IL) lower. Additionally, during walking the muscles are activated reciprocally in contralateral pairs, whereas in jumping both the left and right axial muscles are activated simultaneously. Along with previously recorded activation patterns, our simulations support the idea that when in a walking configuration the pelvis can contribute to hindlimb range of motion effectively *via* lateral rotation, whereas when in a jumping configuration the pelvis is able to take on a stabilisation role. Again, this relationship suggests the effects of counter-torque in the pelvis can be minimised in walking but also in jumping if muscle activations aren’t exactly simultaneous on both sides.

### Hindlimb Muscle Function Cannot Be Fully Inferred From Static Anatomy

For the most part, moment arm outputs for the hindlimb muscles during locomotion are in agreement with the predicted muscle functions published in the literature (Lombard and Abbott, 1907; Kargo and Rome, 2002; Přikryl et al., 2009). There are however a few exceptions. The CR/GL muscles were categorised in the knee extensor functional group based on excitation data from Přikryl et al. (2009), and since they cross both the hip and knee joint, we would expect them to exhibit joint moments about the hip and the knee. Given their biarticular nature, it is unsurprising that these muscles exhibited flexor moments about the hip in the current study. The AM was predicted to be a protractor and adductor of the femur, yet moment arm outputs during walking simulations suggest this muscle is better suited to femur cranial rotation and retraction during walking. The OE was predicted to be a femur retractor however moment arm outputs suggest this muscle is more likely to function as a protractor and cranial long axis rotator. Additionally, IFB and IFM both exhibited variable moments flipping between protractors and retractor moments through the course of the stride cycle despite being predicted retractors.

We propose two alternative reasons for the differences in function between our model and prior literature: 1) geometric variation between species, and 2) moment arm variation due to posture. It is reasonable to expect then, that a more apparent variation in muscle insertion position across different species may have the power to impact muscle moment arm sufficiently to result in a switching of predicted major function. There are also fundamental differences in methodological approach to interpreting muscle function between the data from Přikryl et al. (2009) and the functional data collected from our musculoskeletal model. Since the model presents moment arm data throughout a stride cycle, the major function of the muscle in question is interpreted based on the limb configuration in which the muscle is most likely to be active (i.e., when the MTU was shortening). Kinematics data (Collings et al., 2019) demonstrates the wide range of motion in the hindlimb during walking. Further, the present study highlights the impact that femoral angle can have on moment arm magnitude of thigh muscles. Kargo and Rome (2002) note also that muscle function changed due to hindlimb configuration, Engelkes et al. (2020) also show that humerus position impacts muscle moment arms in the pectoral girdle. It is unlikely that the full range of limb configurations was explored during the excitation study. Thus, differences in reported major functions may be due to differences in limb position throughout the stride cycle.

The proportion of muscles with flexor moments (femur protractors) versus those with extensor moments (femur retractors) was greater than expected. Of the muscles included in our model, eight had flexor moments where only four had extensor moments. This was unexpected given the assumption that limb retraction propels forward motion not only in walking but also jumping. However, while there are twice as many muscle bellies, the split of muscle mass between protractors and retractors is more equal. The AM, SM, and GR are large muscles forming nearly the entire extensor compartment of the hindlimb, whereas the protractors tended to be thin strap muscles or smaller cylindrical muscles (Collings and Richards, 2019). The total mass of the retractors is approximately 0.67 g while the mass of the protractors is only slightly higher at 0.89 g (Collings, unpublished data). These observations suggest that protraction and limb position require more precision to place the hindlimb in the correct configuration to be ready for a more powerful retraction to drive forward motion in the desired direction.

### Both Pelvic Lateral Rotation and Hindlimb Angle Impact Muscle Moments

Given that limb configuration has previously been shown to impact moment arms and subsequent muscle functions (Kargo and Rome, 2002; Engelkes et al., 2020), and dorsoventral rotation of the pelvis impacted muscle functions during Lombard and Abbott’s (1907) excitation study, we predicted that pelvic lateral rotation would alter the moment arm relationships of the muscles spanning the hip joint. We investigated this by generating and comparing the outputs of a range of hypothetical trials and an experimental walking simulation. The range of hypothetical trials demonstrated that pelvic lateral rotation and hindlimb position influences the magnitude of many of the MTU moment arms (especially Flex/Ex). The position of the femur also in some instances changed the sinusoidal moment arm pattern, such as in the AM where the moment arm magnitudes got stronger as the pelvis rotated to the left when the femur was held at 90° or 135° but got weaker while rotating to the left when held at 10°. Since walking entails a synchronised combination of both pelvic lateral rotation and femur protraction/retraction, the timing of these two motions not only impacts the ability of the pelvis to contribute to limb retraction in terms of stride length (Collings et al., 2019), but also in terms of muscle mechanics.

Pelvic lateral rotation had a differential impact on the flexor and extensor hindlimb muscle moment arms, depending on their function. Those muscles with protractor moments and some of the muscles with retractor moments (SM and GR) benefitted from pelvic lateral rotation with increased moments. The fluctuation in moment arm magnitude was also increased in the fixed pelvic simulations suggesting that pelvic lateral rotation dampens the effect of femur angle throughout the stride cycle, therefore allowing the moments to stay as strong as they could be at each point during the stride cycle (or given femur position).

Of course, the current interpretations come with the caveat of being true given that all else remains equal. In reality, the animals would likely compensate for the reduced moment if their pelvis were to be fixed anatomically. The present walking simulations combined with previous kinematic studies investigating the impact on stride length (Collings et al., 2019) suggest that pelvic lateral rotation is not required for walking but that it does contribute by making it slightly mechanically easier. With a fixed pelvis, the muscles would need to work slightly harder to generate equal torque.

### The Results of Our Computational Approach Become Hypotheses for Future Experiments

In addition to the caveat above, the present study has several limitations due to its computational approach which can be placed into four categories: MTU morphology, Muscle force, Muscle activation, Bone kinematics.

### MTU Morphology: Wrapping Surfaces Don’t Capture the Morphology Exactly

The inclusion of wrapping surfaces into the construction of the model puppet did allow for muscle pathways to be mimicked however it is not possible to capture all details of the muscle and tendon architecture in our model. For example, the MTUs in MuJoCo assume the muscle and tendon components function in unison to generate net length change. Resolving the independent change in muscle fascicle length versus tendon stretch. Thus, MTU function in this paper is an assumption based on muscle leverage. To resolve, the model output could be compared with tendon travel experiments and sonomicrometry as in Konow et al. (2012).

### Muscle Force: We Do Not Know Muscle Forces

While we can calculate muscle moment arm, we do not have the data output from this model to resolve muscle force output. With further work using an inverse dynamics approach to calculate predicted muscle force from the joint kinematics and MTU geometry, force outputs can be predicted.

### Muscle Activation: We Cannot Verify Muscle Activation

This means that although we assume that when an MTU is shortening the muscle would be actively contracting we cannot verify muscle activation patterns for eccentric muscle activations. Where possible we have verified MTU length changes with previously published EMG data however, unlike the pelvic muscles, there are currently no published muscle activation data for any of the hindlimb muscles during walking. However, the present model allows muscles of interest to be identified for the informed planning of future *in vivo* studies. With EMG and sonomicrometry data, for example, activation timings and length changes for the muscles can resolve whether muscles are concentrically or eccentrically contracting. It is suggested that EMG data is collected for the major muscles of the hindlimb and compared with the moment arm data collected here to allow further resolution of hindlimb muscle function during walking.

### Bone Kinematics: Joint Kinematics Were Estimated From Surface Markers Only

This paper took a non-invasive approach to collecting kinematic data, but this does mean that one notable assumption that we cannot verify is the long axis rotations of the femur, tibiofibula and tarsal segments. Since our experimental set-up did not resolve these empirically, we worked with the assumption that these segments would be rotated about their long axis in a manner which consistently aligned their flexion/extension axes. Experimental observation of the skeletal kinematics [using xray reconstruction of moving morphology (XROMM)] during walking is required to confirm or challenge this assumption, and to assess to what degree MTU and moment arm changes are sensitive to long axis rotations. Our anatomical model coupled with the kinematic motion can highlight bones of interest and assist in identifying potential implant sites and surgery strategies for the bone markers required for XROMM.

Despite the limitations, our approach is extremely valuable because it allows precise hypotheses to be generated that can then be directly addressed with further experimental work and computational work (e.g., inverse dynamics etc.).

## Conclusion

The following conclusions were drawn from the data presented in this paper:

1. Pelvic dorsoventral rotation and pelvic lateral rotation angle have the power to impact moment arms of the axial and hindlimb muscles crossing the hip joint.
2. In walking postures, axial muscle moment arms are at their strongest in the lateral rotation plane and hindlimb muscles are strongest in Flex/Ex plane.
3. Pelvic lateral rotation contributes to limb motion by strengthening flexor (and some extensor) moment arms in the hindlimb muscles.

*P. maculatus* thus appear to have a musculoskeletal anatomy that enables them to modulate pelvic and hindlimb motion with alternative activation patterns and postural changes, respectively, ultimately permitting multifunctionality.

## Data Availability

All XML files, supporting files, and custom code are available in the follwing GitHub repository https://github.com/frogtronics/mujoco150_KM.
