# Supplementary figures and images for: Functional Analysis of Anuran Pelvic and Thigh Anatomy Using Musculoskeletal Modelling of Phlyctimantis maculatus
Source: Front Bioeng Biotechnol. 2022 Apr 1;10:806174. doi: 10.3389/fbioe.2022.806174 (PMC9011185; doi:10.3389/fbioe.2022.806174)

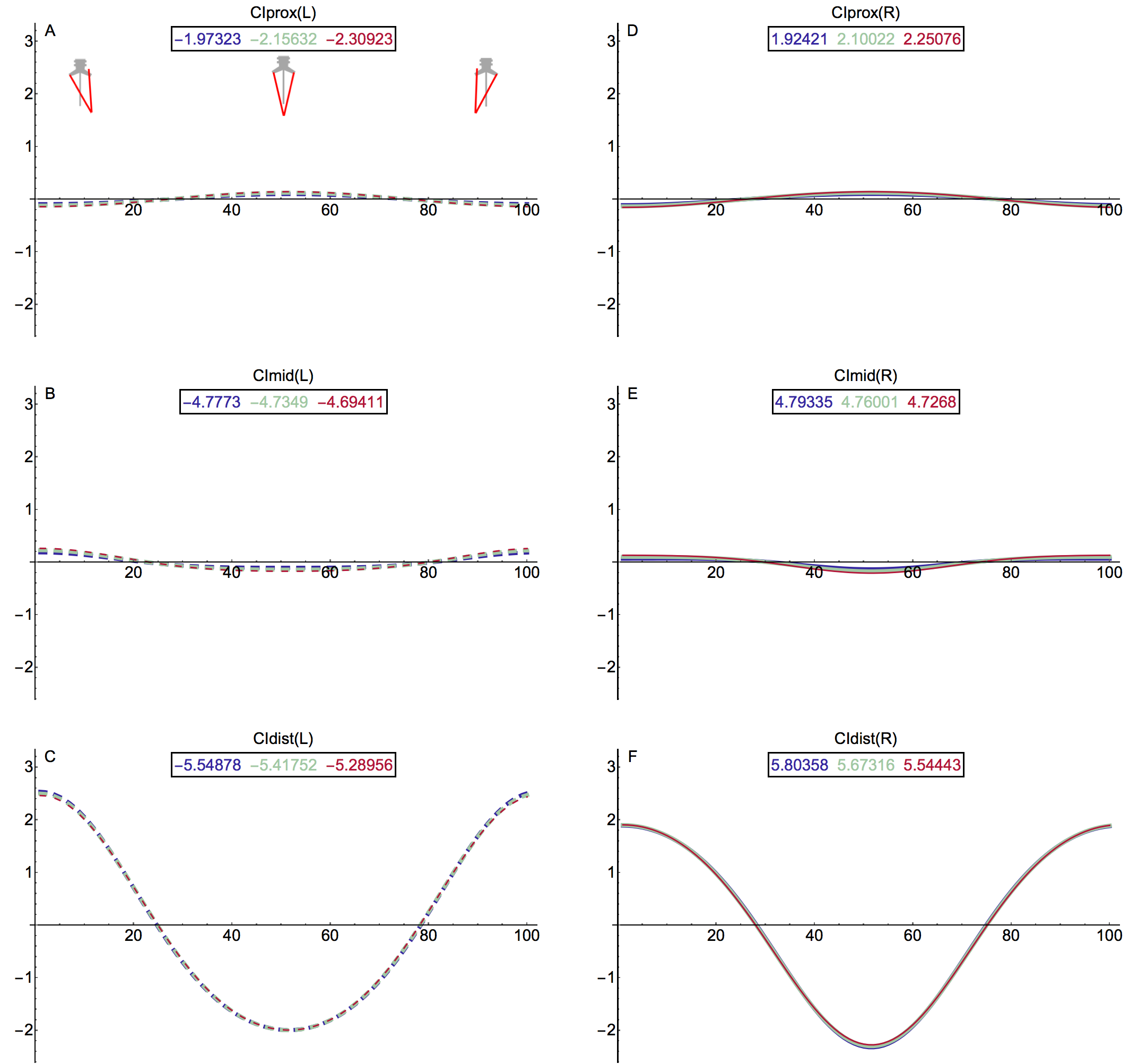

Supplement: Supplementary file 3 [file DataSheet2.ZIP › FigureSI_HYP_CI_LatRotation_relativeScaling.png]

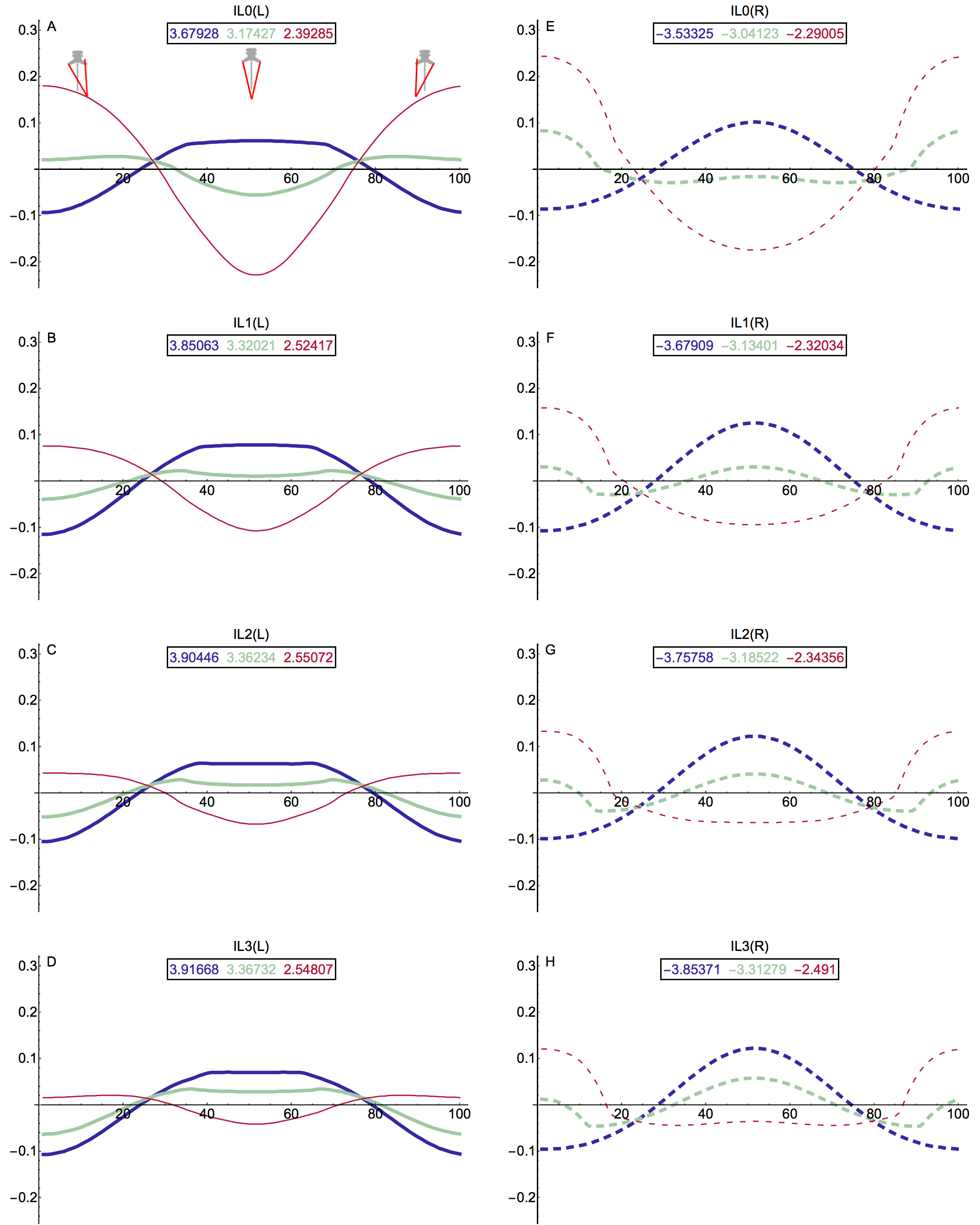

Supplement: Supplementary file 3 [file DataSheet2.ZIP › FigureSI_HYP_IL_LatRotation_relativeScaling.png]

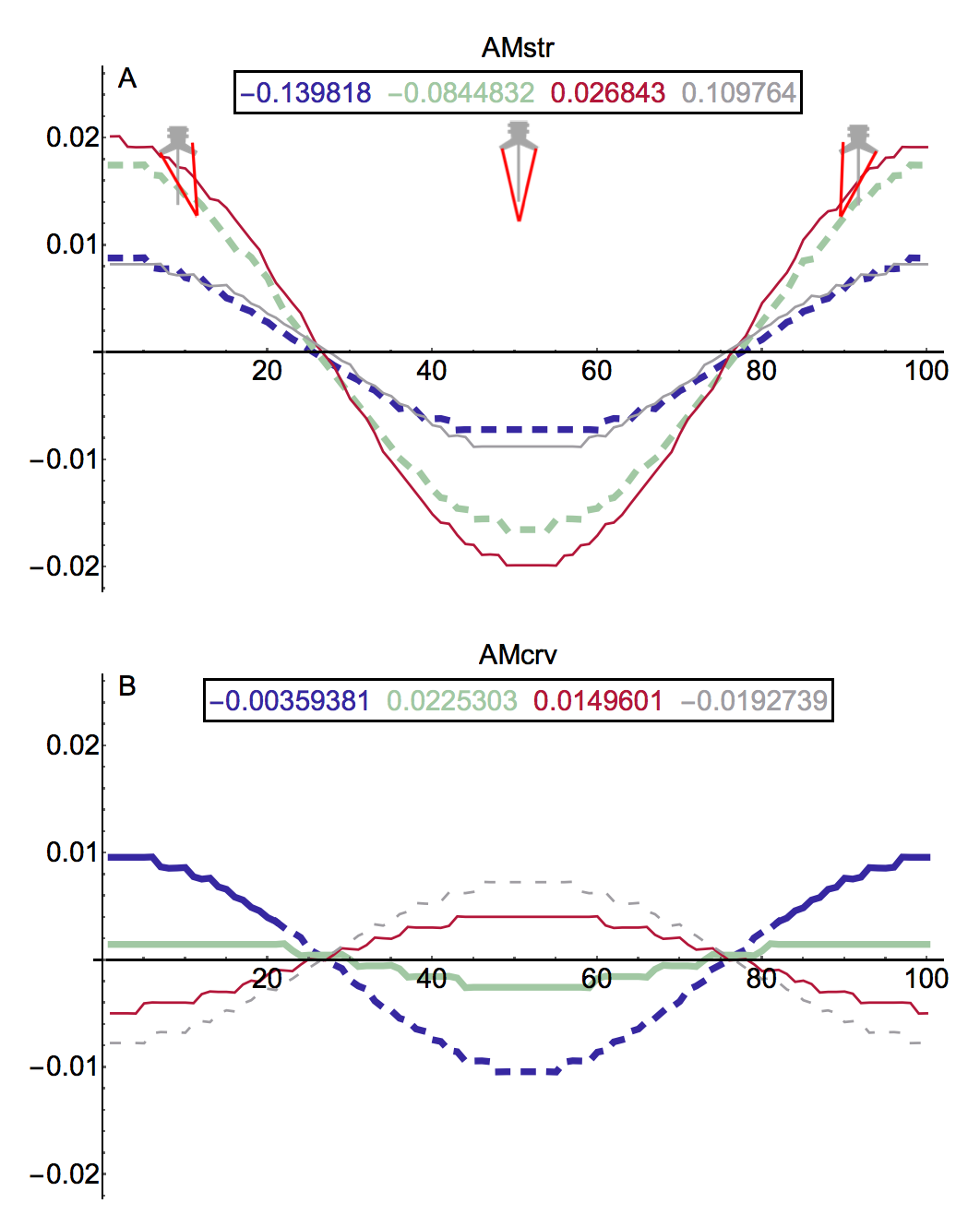

Supplement: Supplementary file 3 [file DataSheet2.ZIP › FigureSI_HYP_LAM_AA_relativeScaling.png]

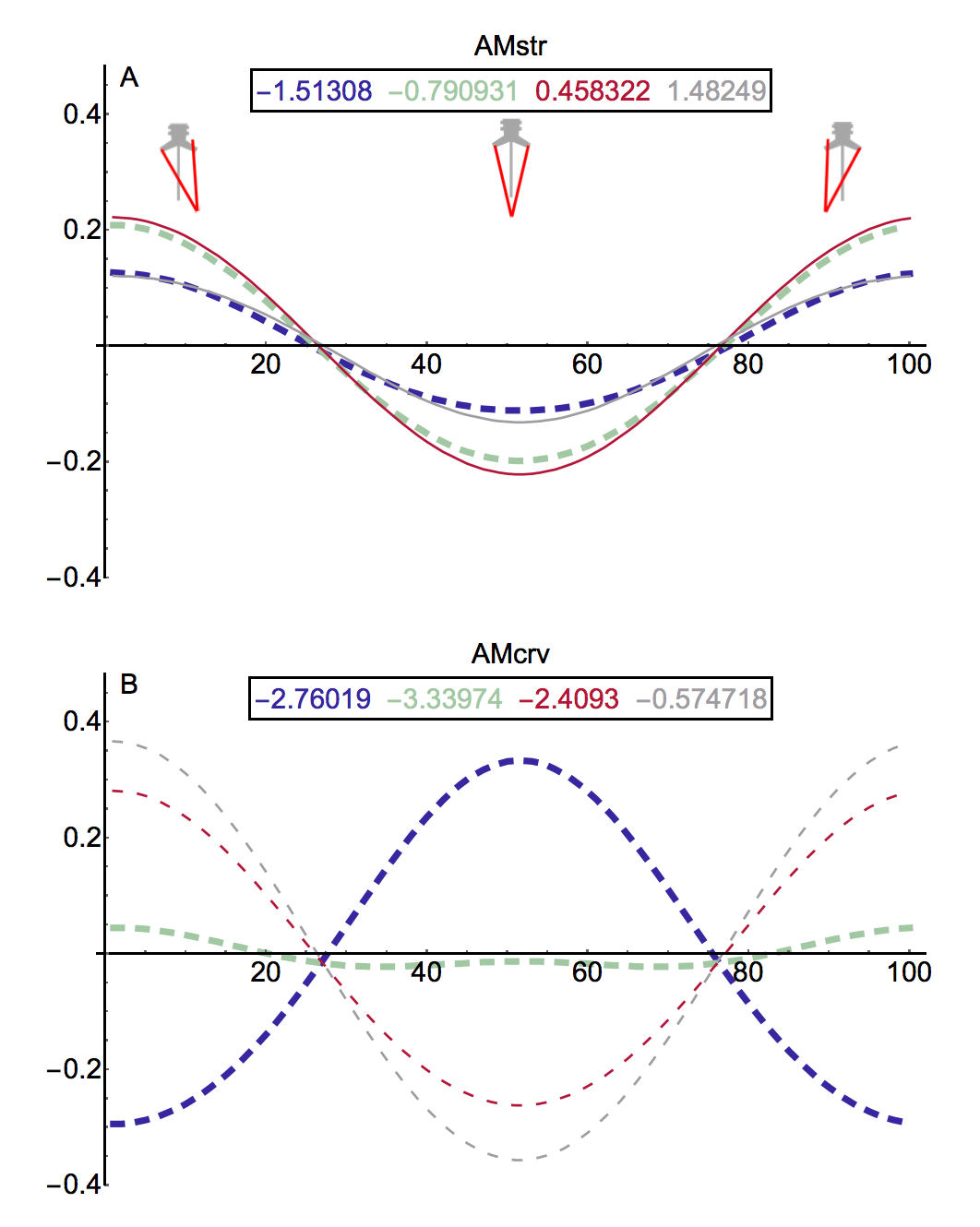

Supplement: Supplementary file 3 [file DataSheet2.ZIP › FigureSI_HYP_LAM_FE_relativeScaling.png]

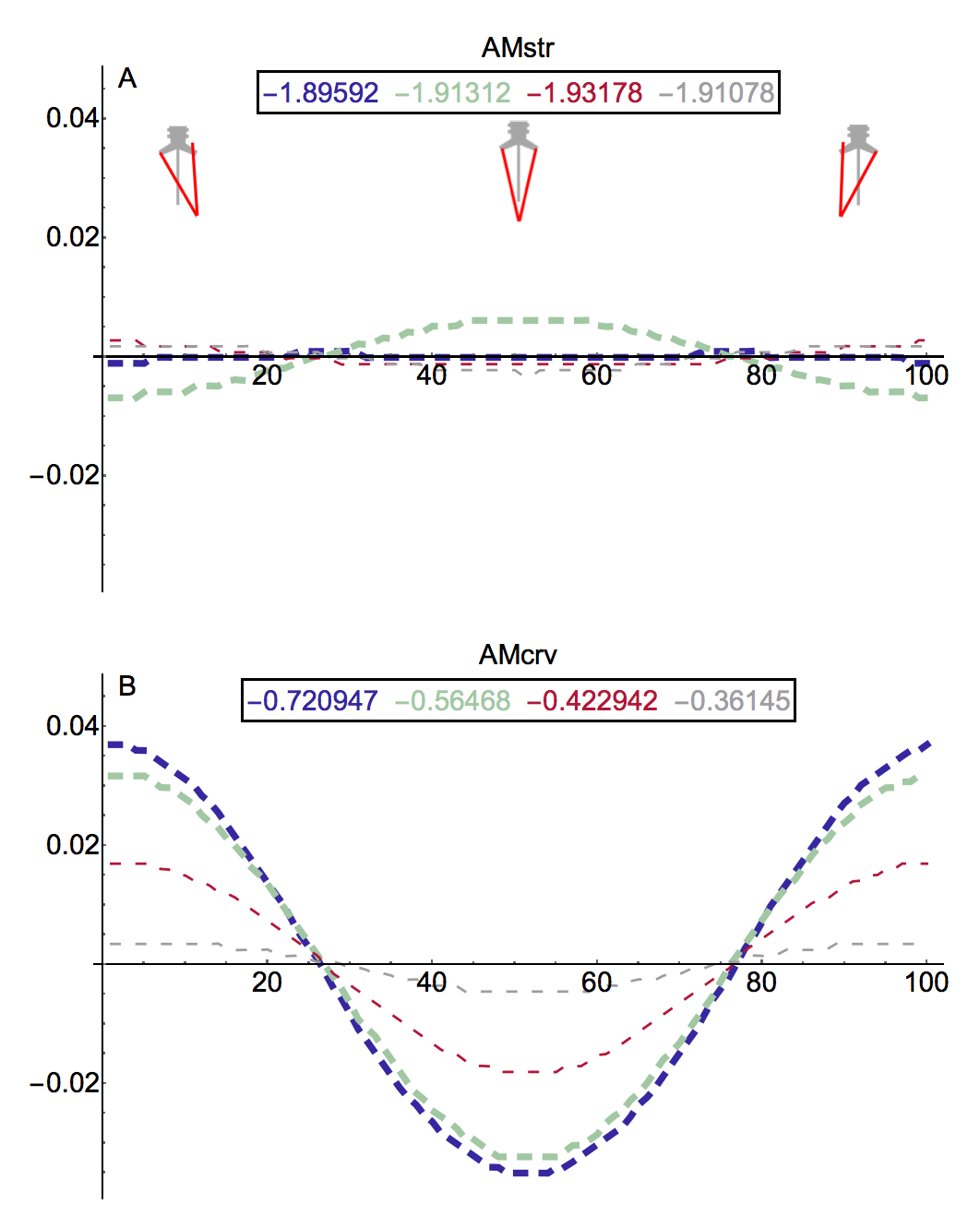

Supplement: Supplementary file 3 [file DataSheet2.ZIP › FigureSI_HYP_LAM_LAR_relativeScaling.png]

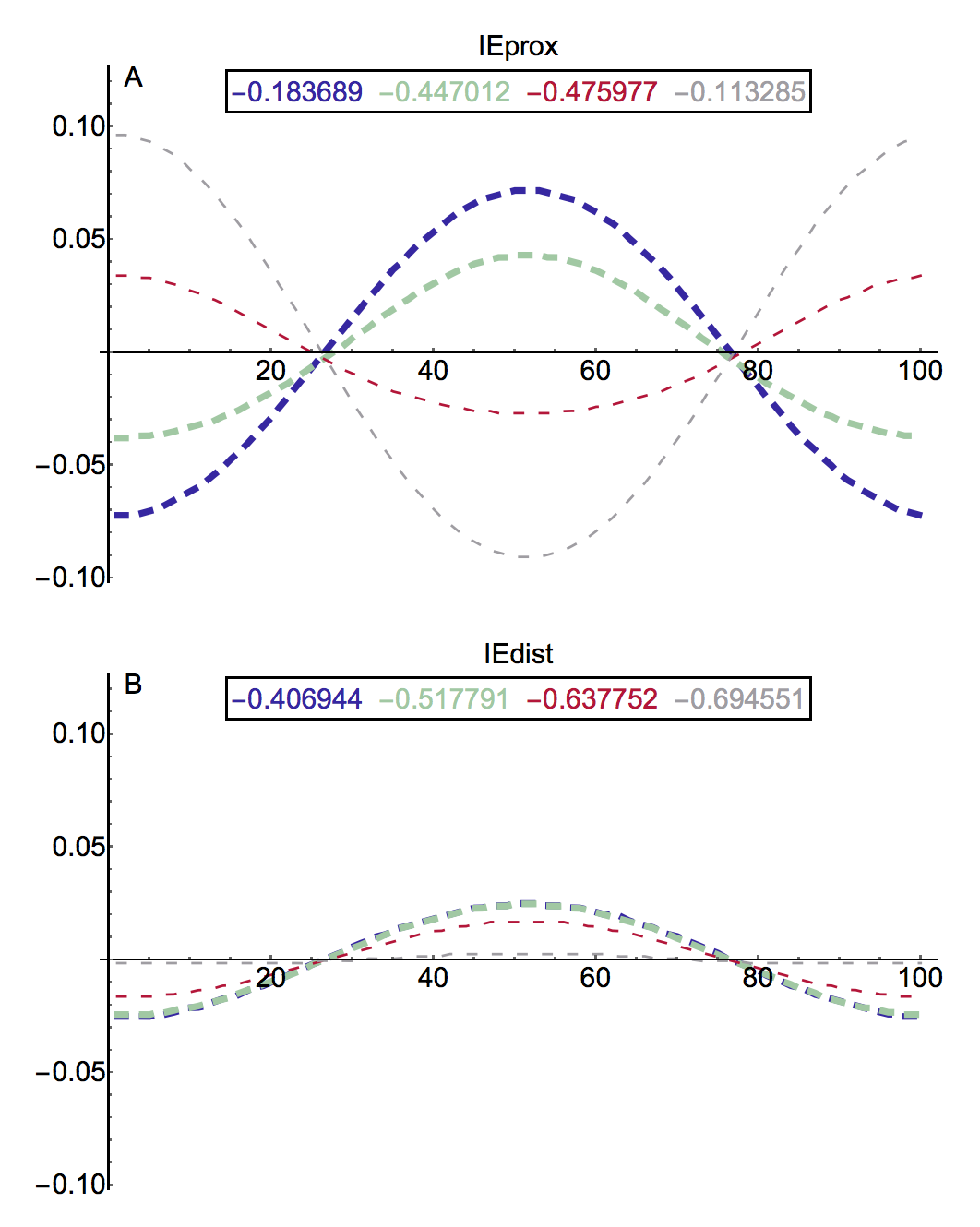

Supplement: Supplementary file 3 [file DataSheet2.ZIP › FigureSI_HYP_LIE_AA_relativeScaling.png]

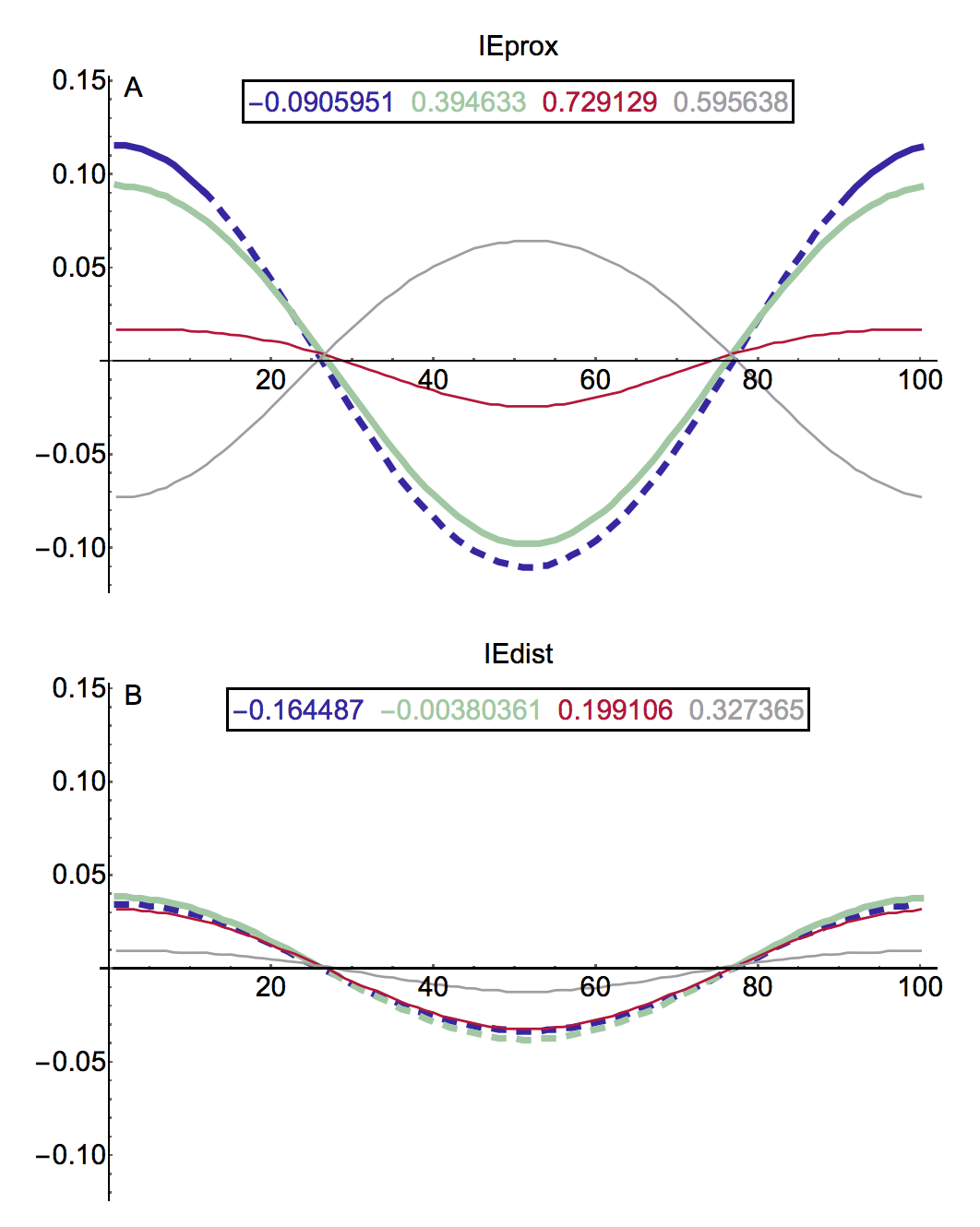

Supplement: Supplementary file 3 [file DataSheet2.ZIP › FigureSI_HYP_LIE_FE_relativeScaling.png]

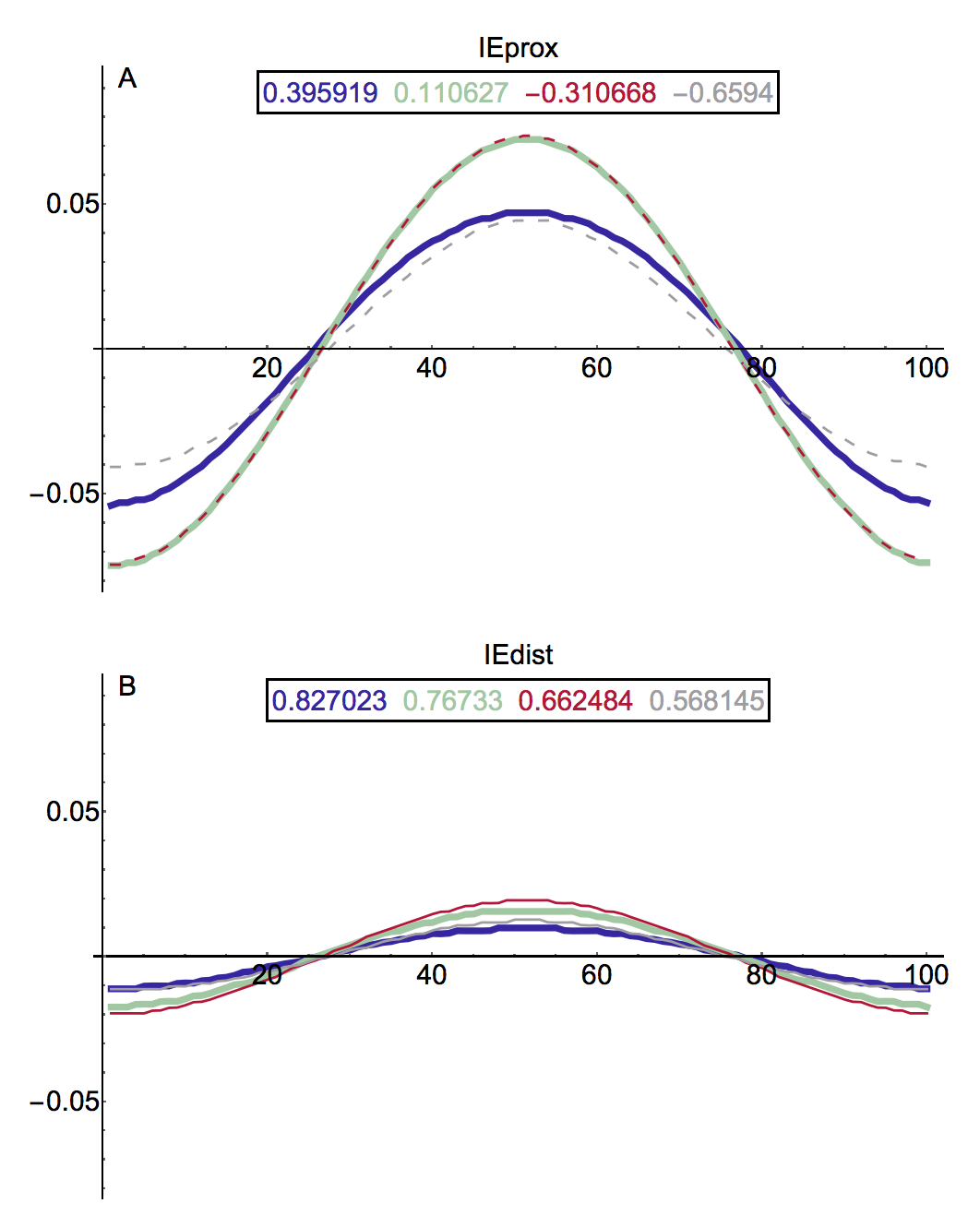

Supplement: Supplementary file 3 [file DataSheet2.ZIP › FigureSI_HYP_LIE_LAR_relativeScaling.png]

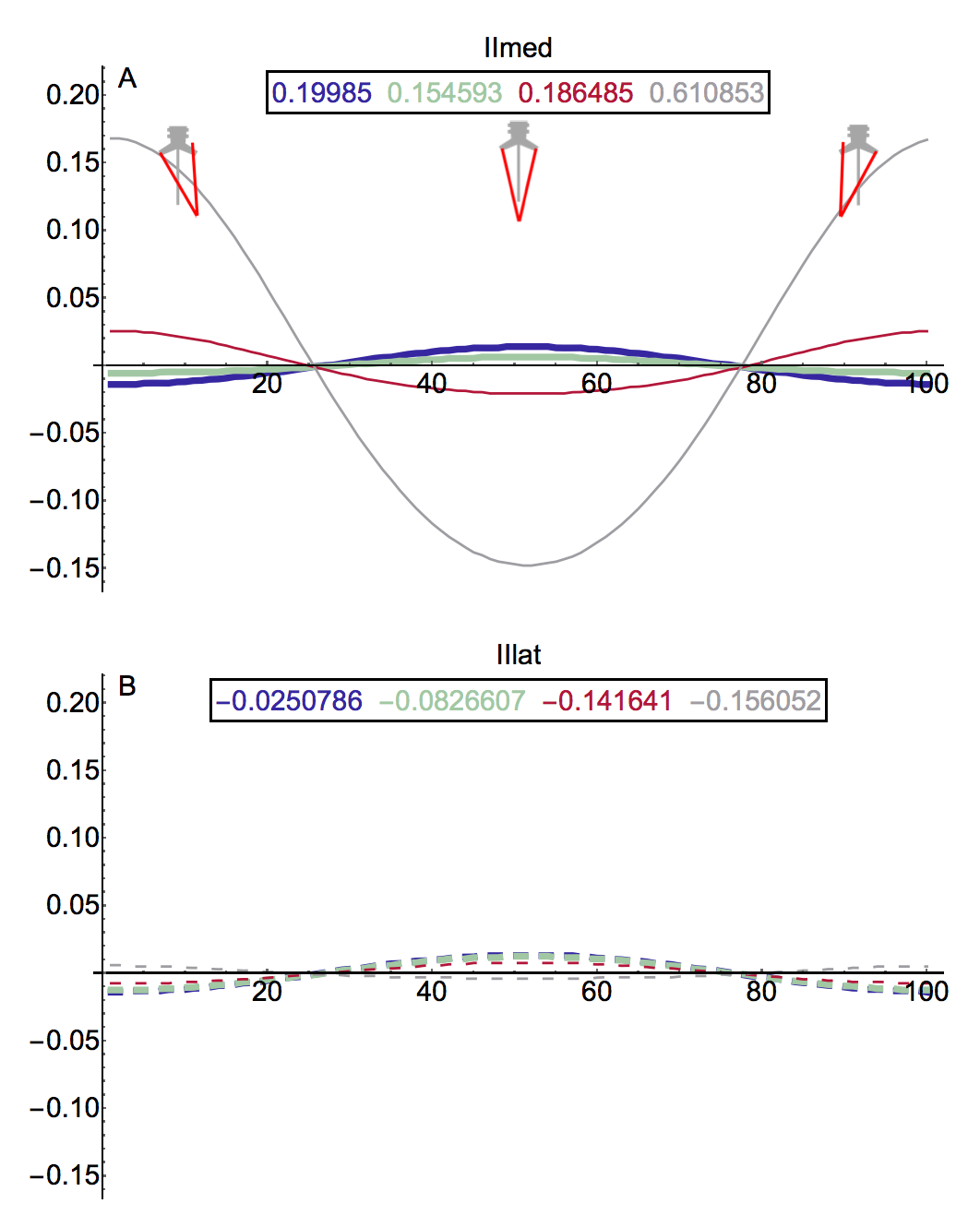

Supplement: Supplementary file 3 [file DataSheet2.ZIP › FigureSI_HYP_LII_AA_relativeScaling.png]

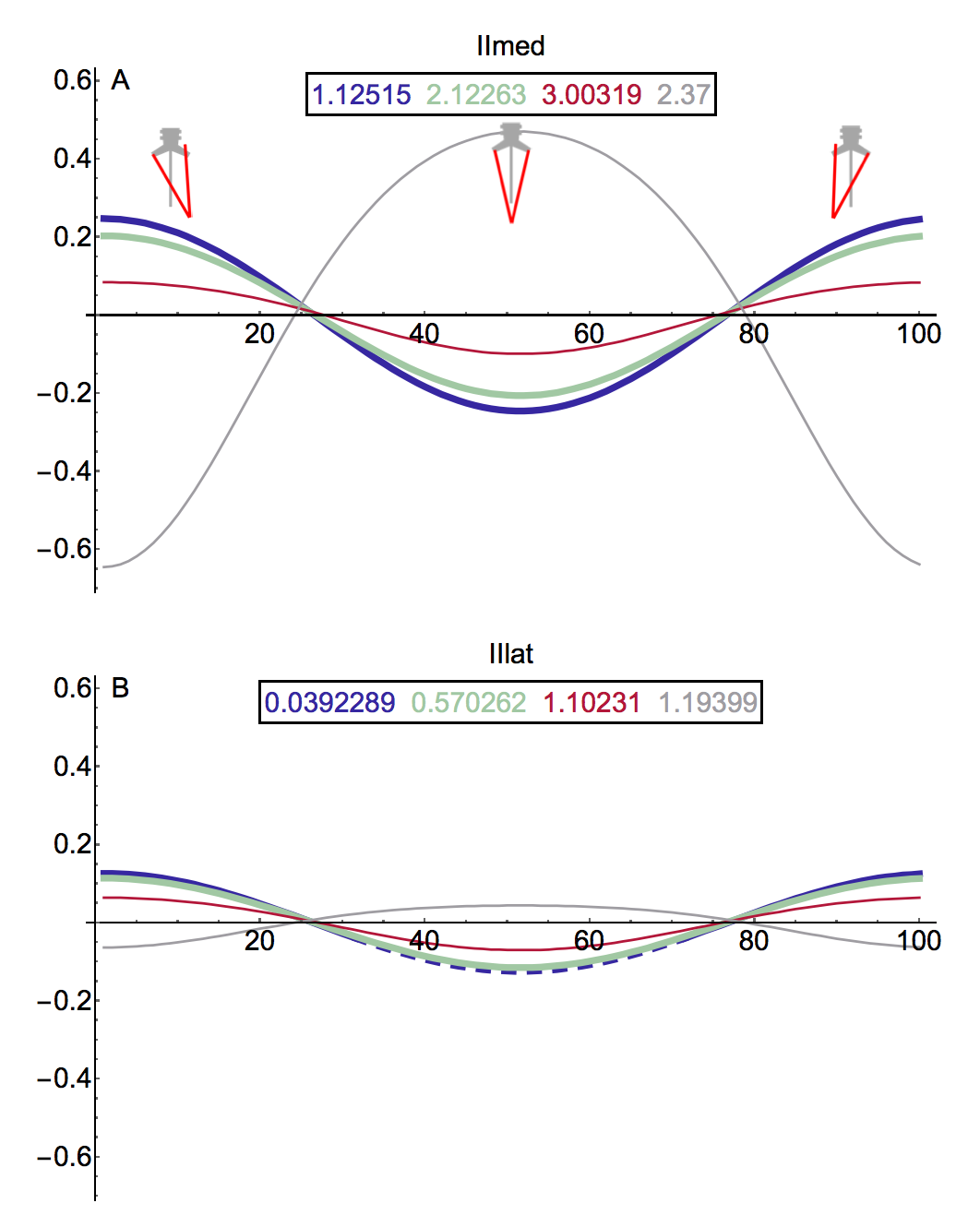

Supplement: Supplementary file 3 [file DataSheet2.ZIP › FigureSI_HYP_LII_FE_relativeScaling.png]

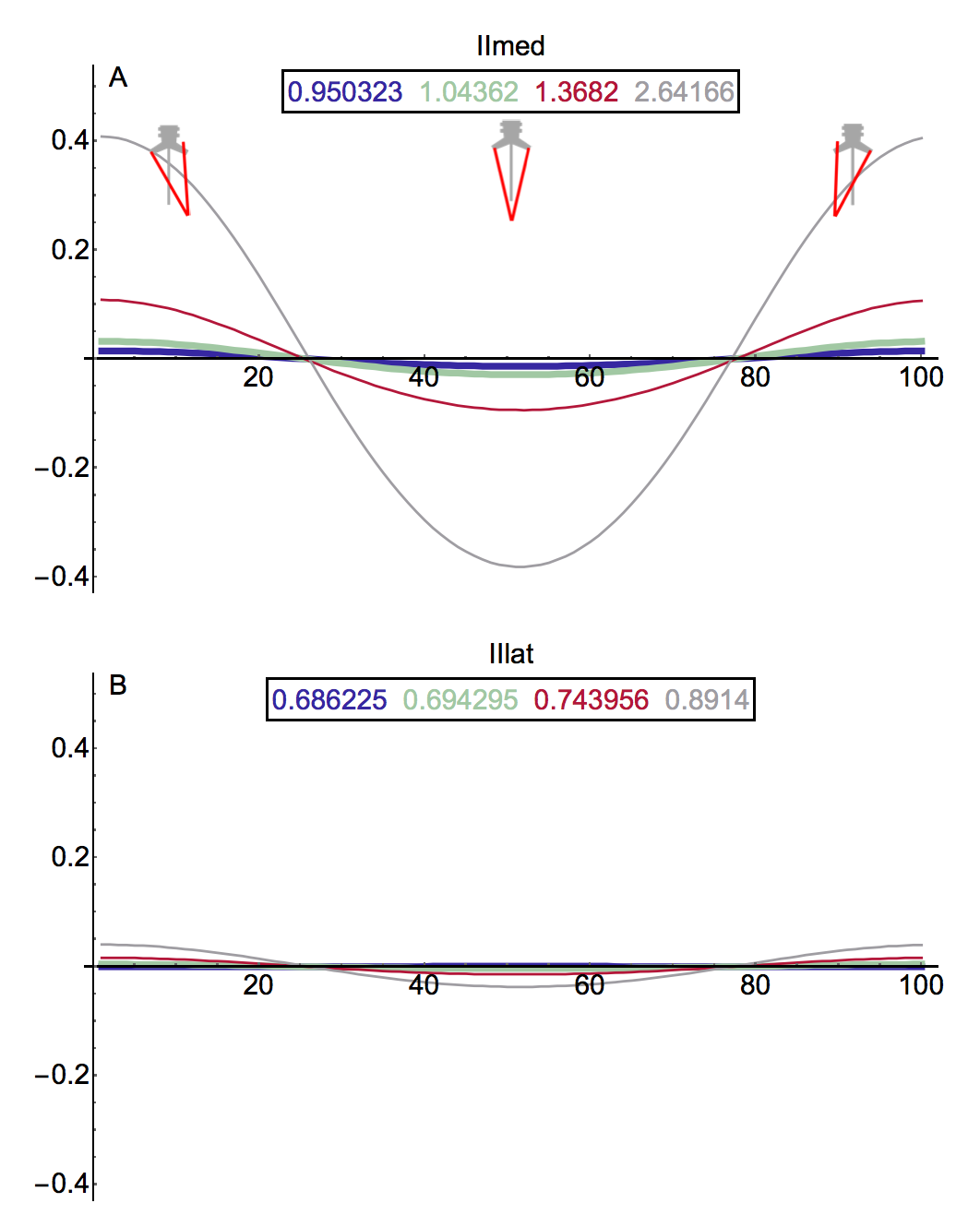

Supplement: Supplementary file 3 [file DataSheet2.ZIP › FigureSI_HYP_LII_LAR_relativeScaling.png]

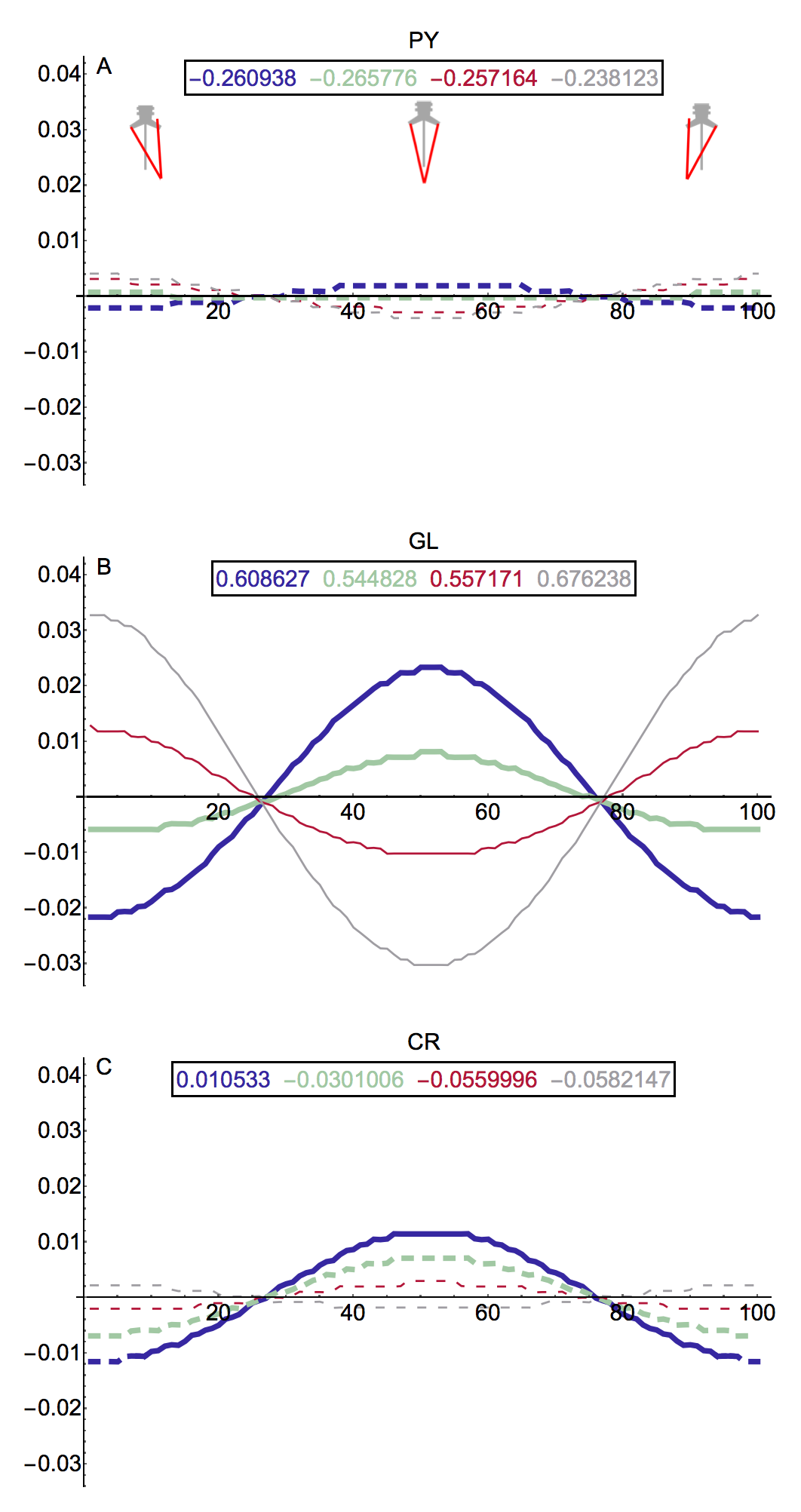

Supplement: Supplementary file 3 [file DataSheet2.ZIP › FigureSI_HYP_misc_AA_relativeScaling.png]

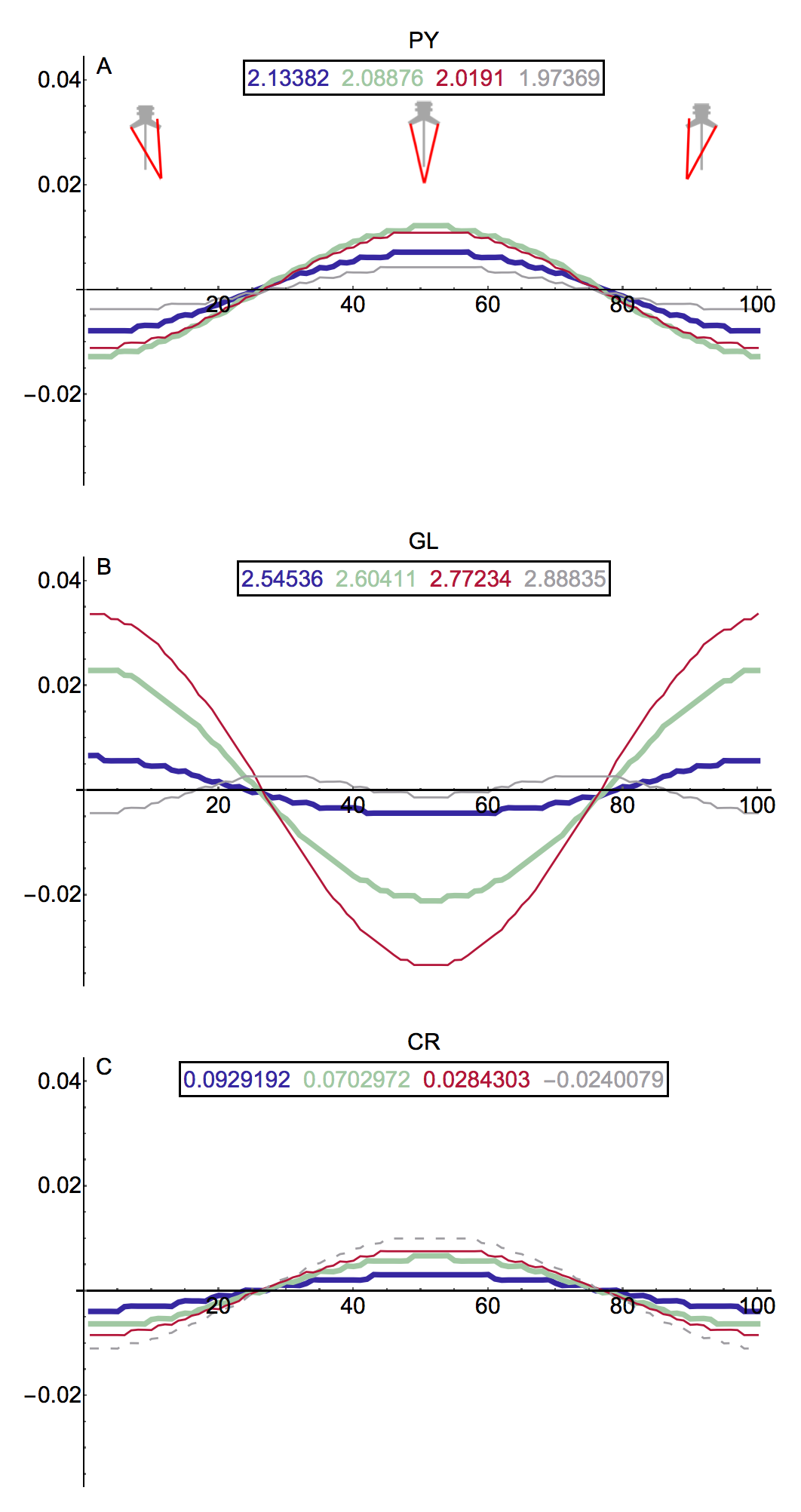

Supplement: Supplementary file 3 [file DataSheet2.ZIP › FigureSI_HYP_misc_LAR_relativeScaling.png]

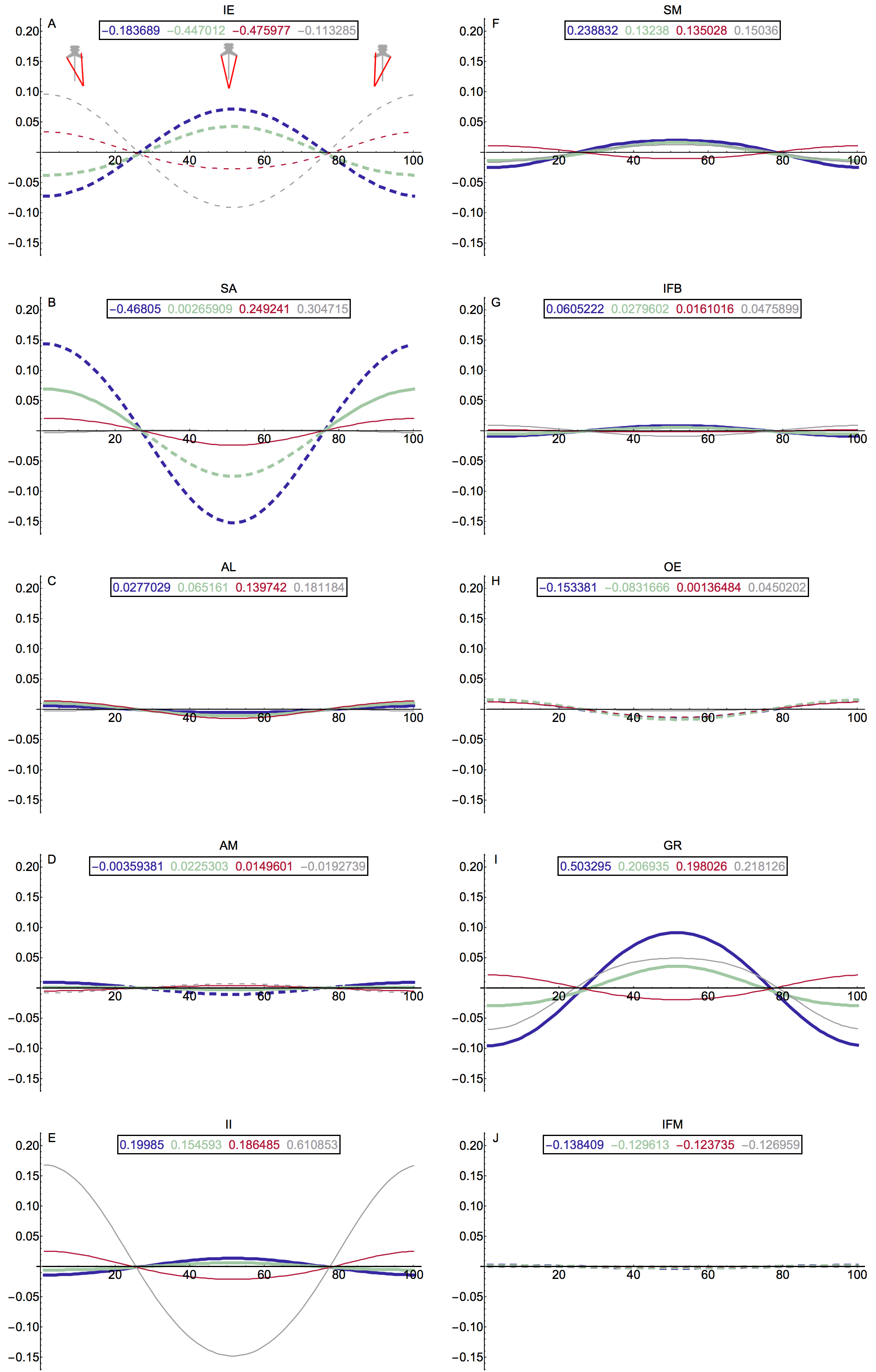

Supplement: Supplementary file 3 [file DataSheet2.ZIP › FigureSI_HYP_pro&ret_AA_relativeScaling.png]

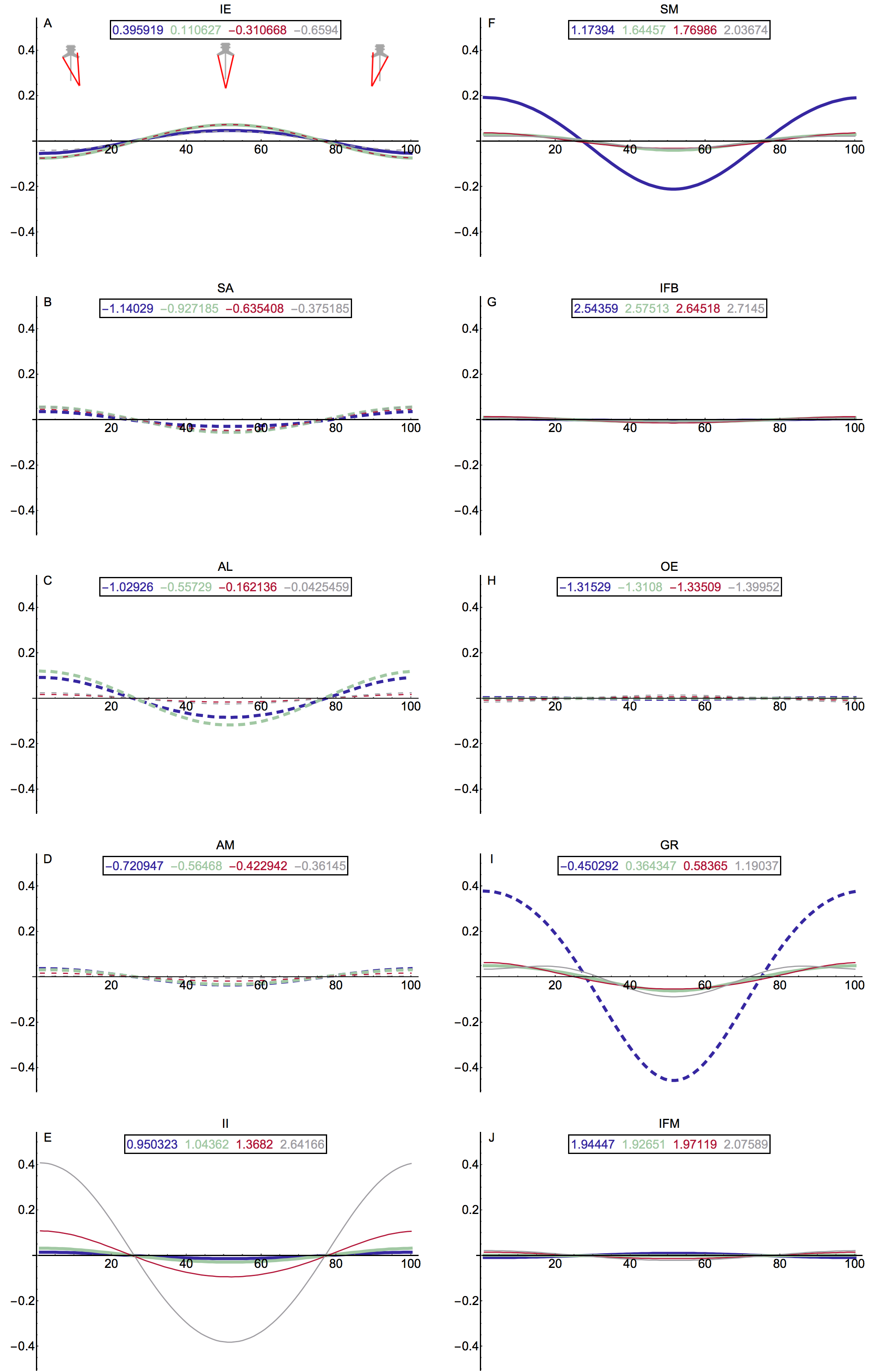

Supplement: Supplementary file 3 [file DataSheet2.ZIP › FigureSI_HYP_pro&ret_LAR_relativeScaling.png]

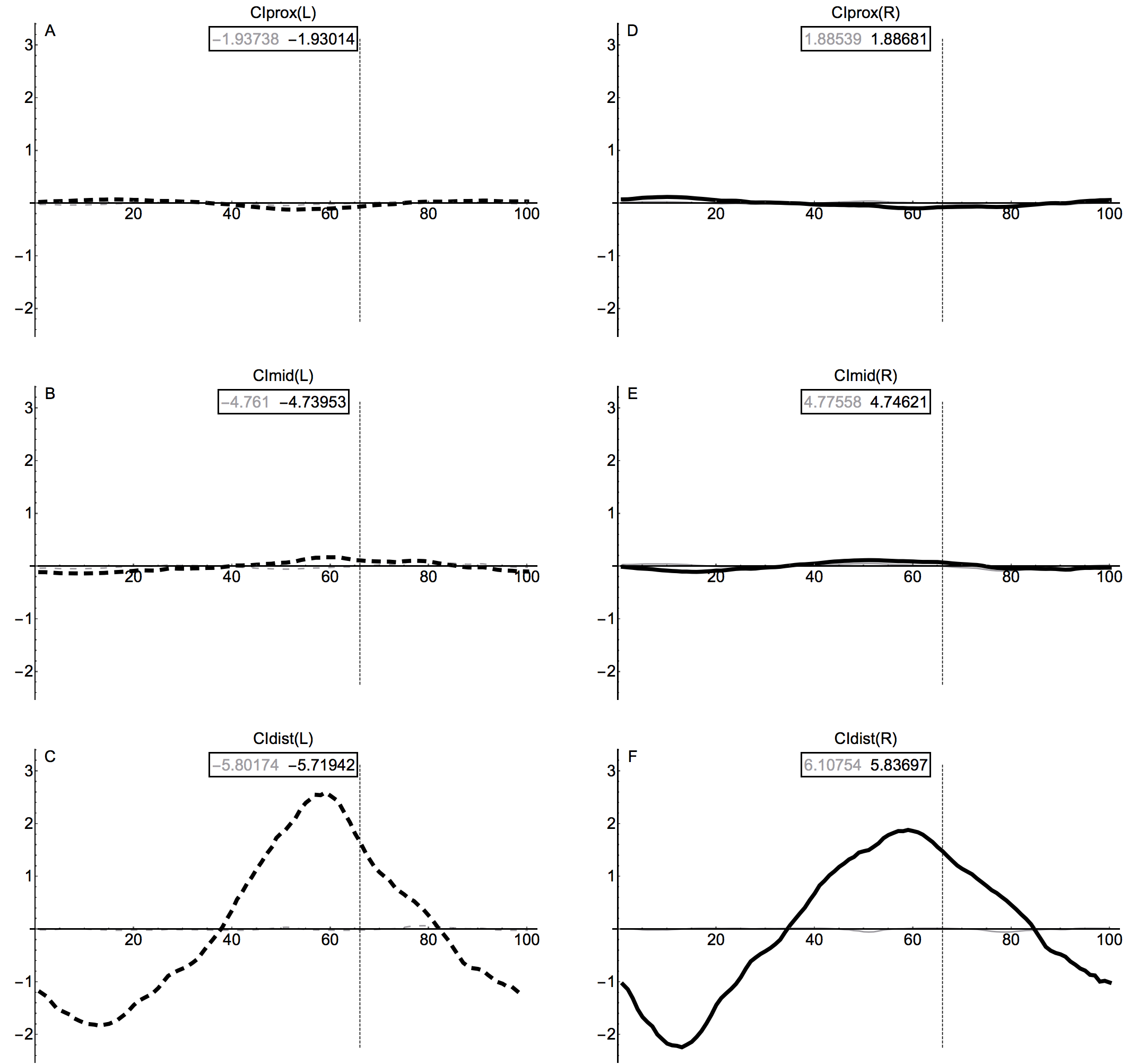

Supplement: Supplementary file 3 [file DataSheet2.ZIP › FigureSI_RUN_CI_LatRotation_relativeScaling.png]

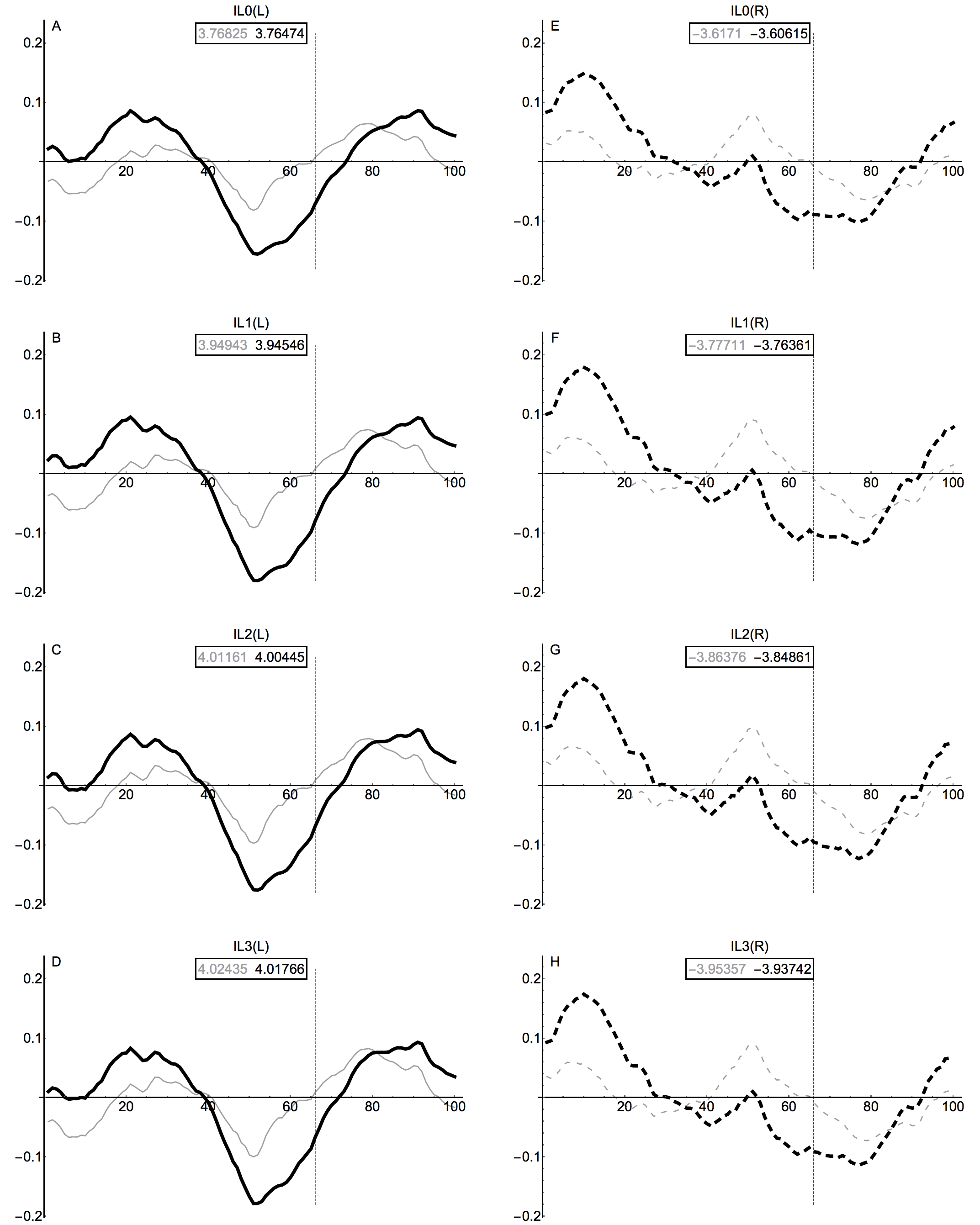

Supplement: Supplementary file 3 [file DataSheet2.ZIP › FigureSI_RUN_IL_LatRotation_relativeScaling.png]

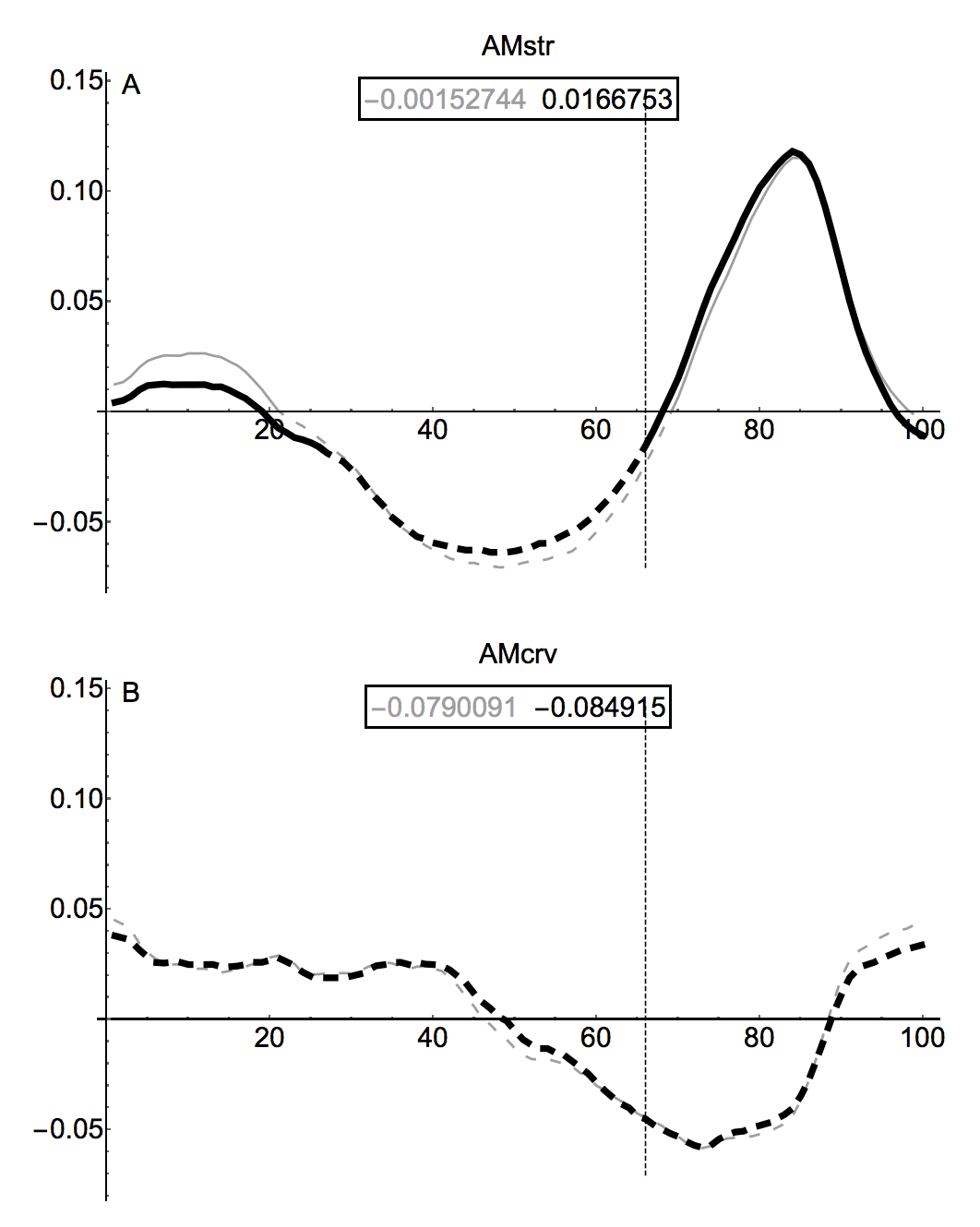

Supplement: Supplementary file 3 [file DataSheet2.ZIP › FigureSI_RUN_LAM_AA_relativeScaling.png]

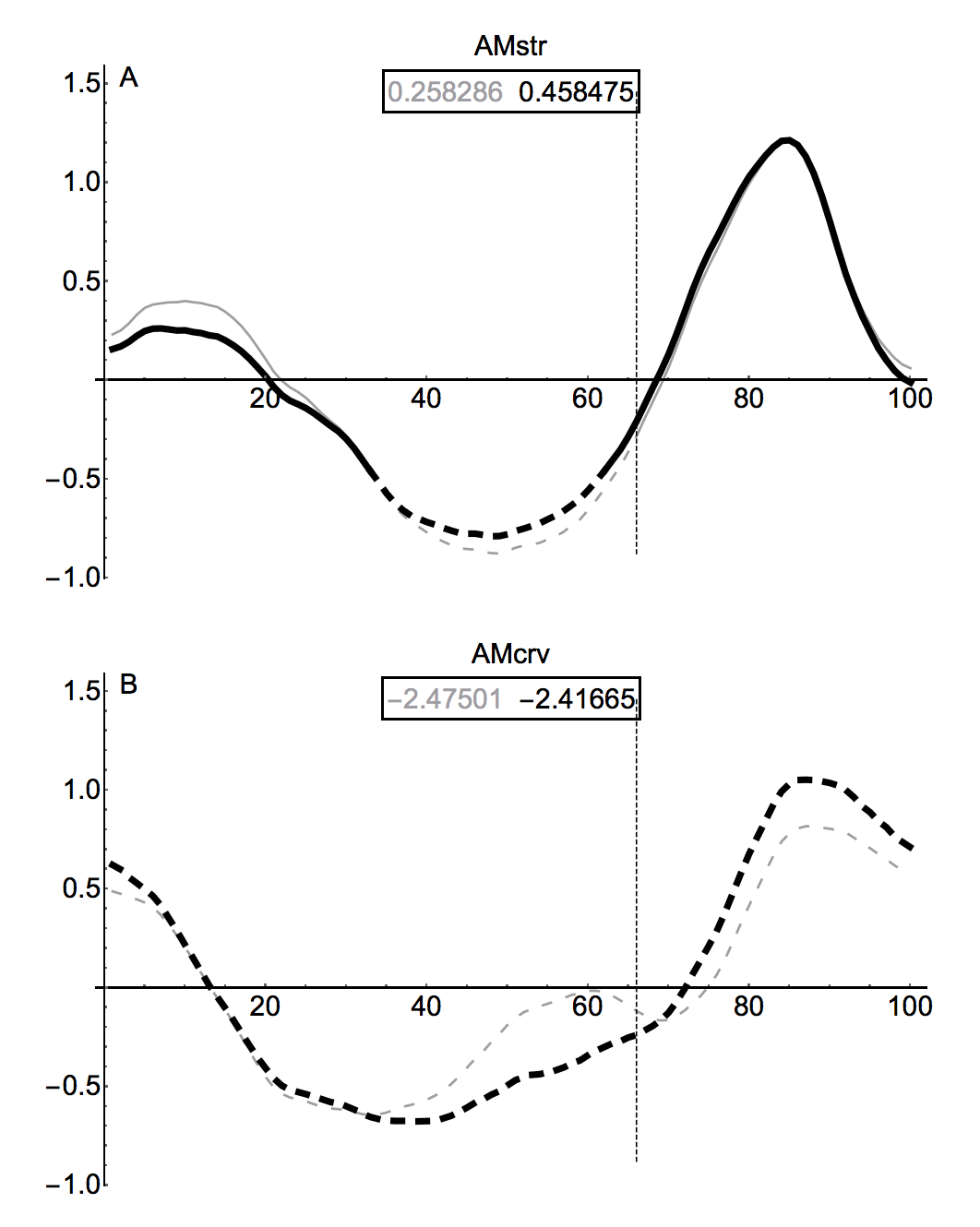

Supplement: Supplementary file 3 [file DataSheet2.ZIP › FigureSI_RUN_LAM_FE_relativeScaling.png]

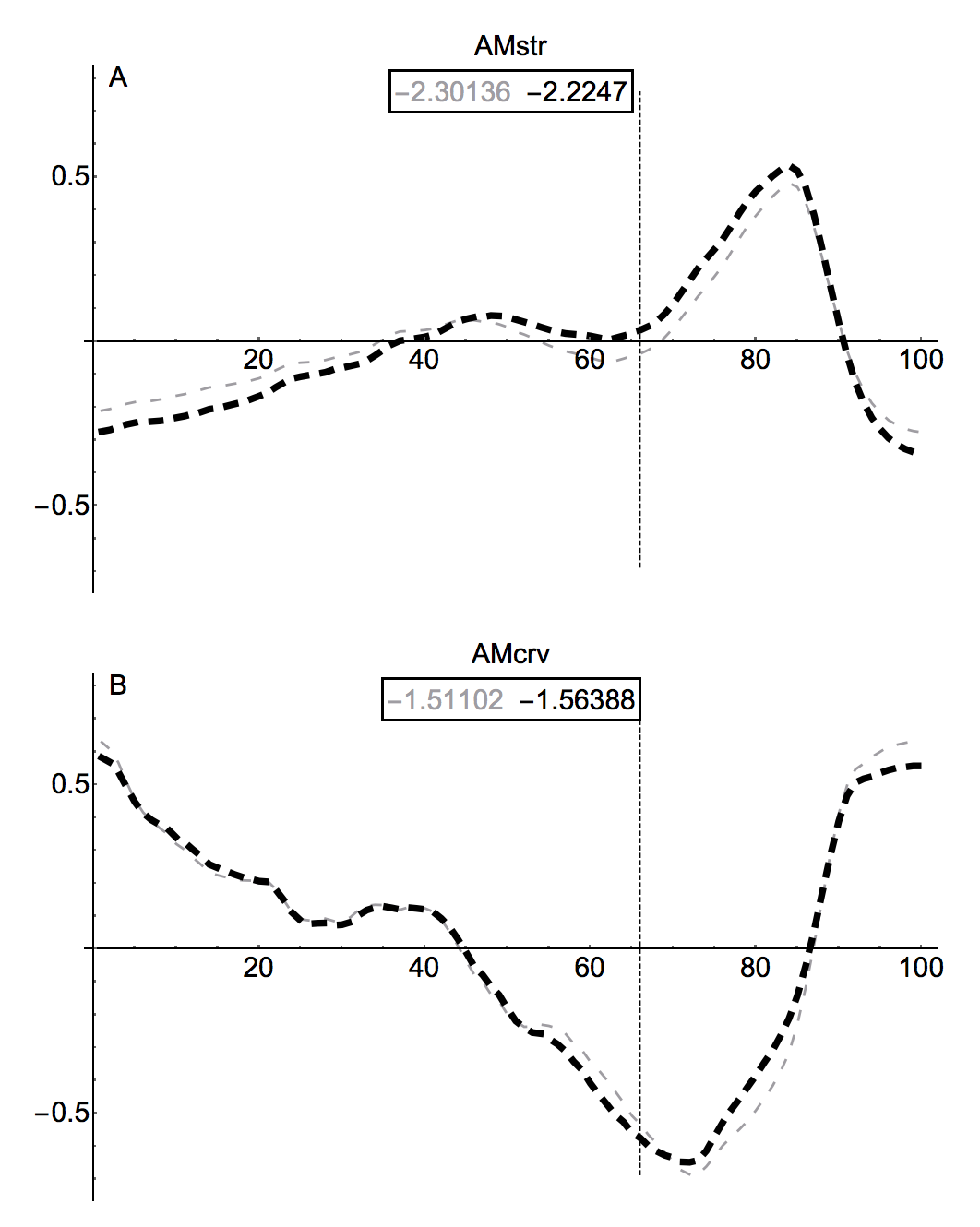

Supplement: Supplementary file 3 [file DataSheet2.ZIP › FigureSI_RUN_LAM_LAR_relativeScaling.png]

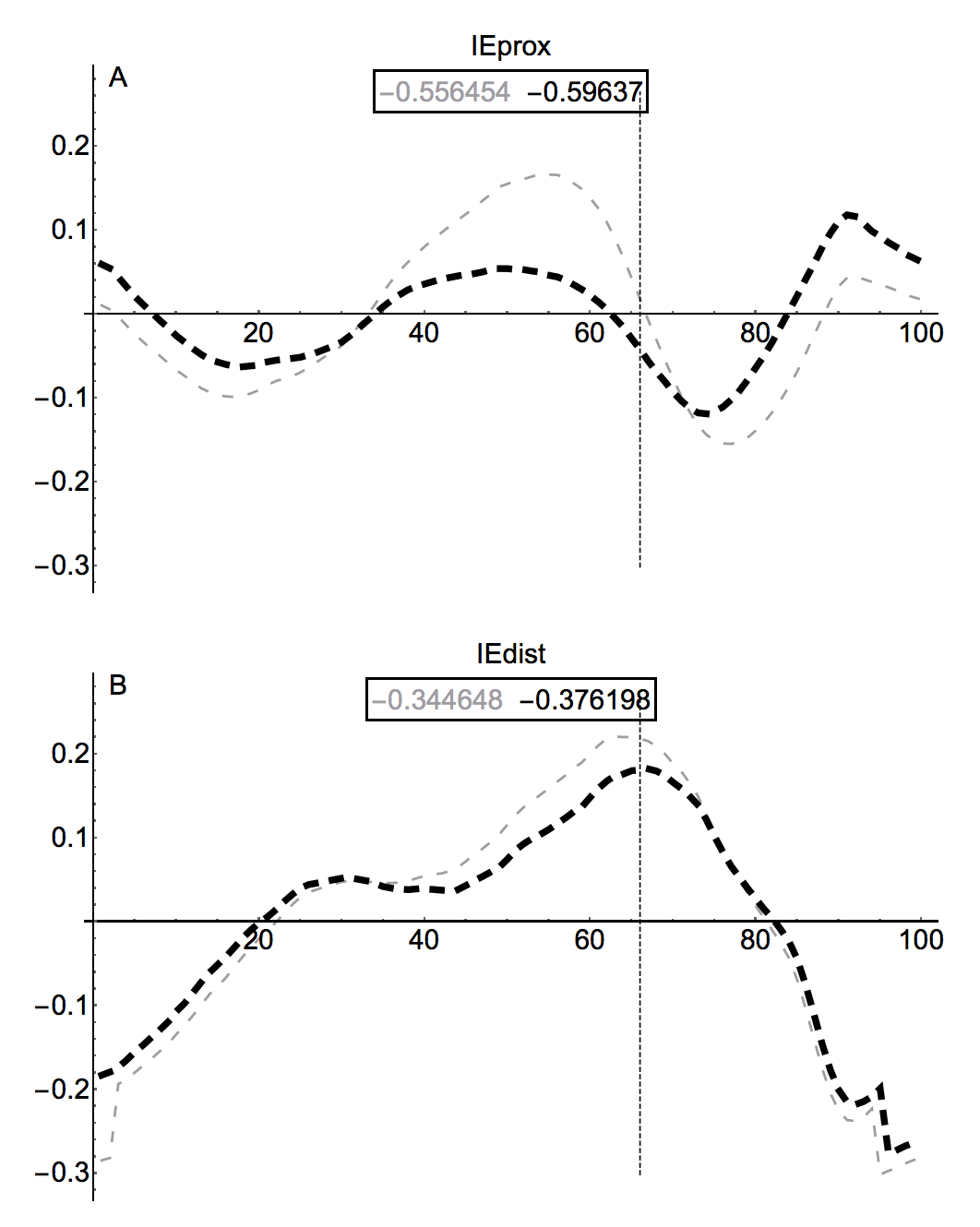

Supplement: Supplementary file 3 [file DataSheet2.ZIP › FigureSI_RUN_LIE_AA_relativeScaling.png]

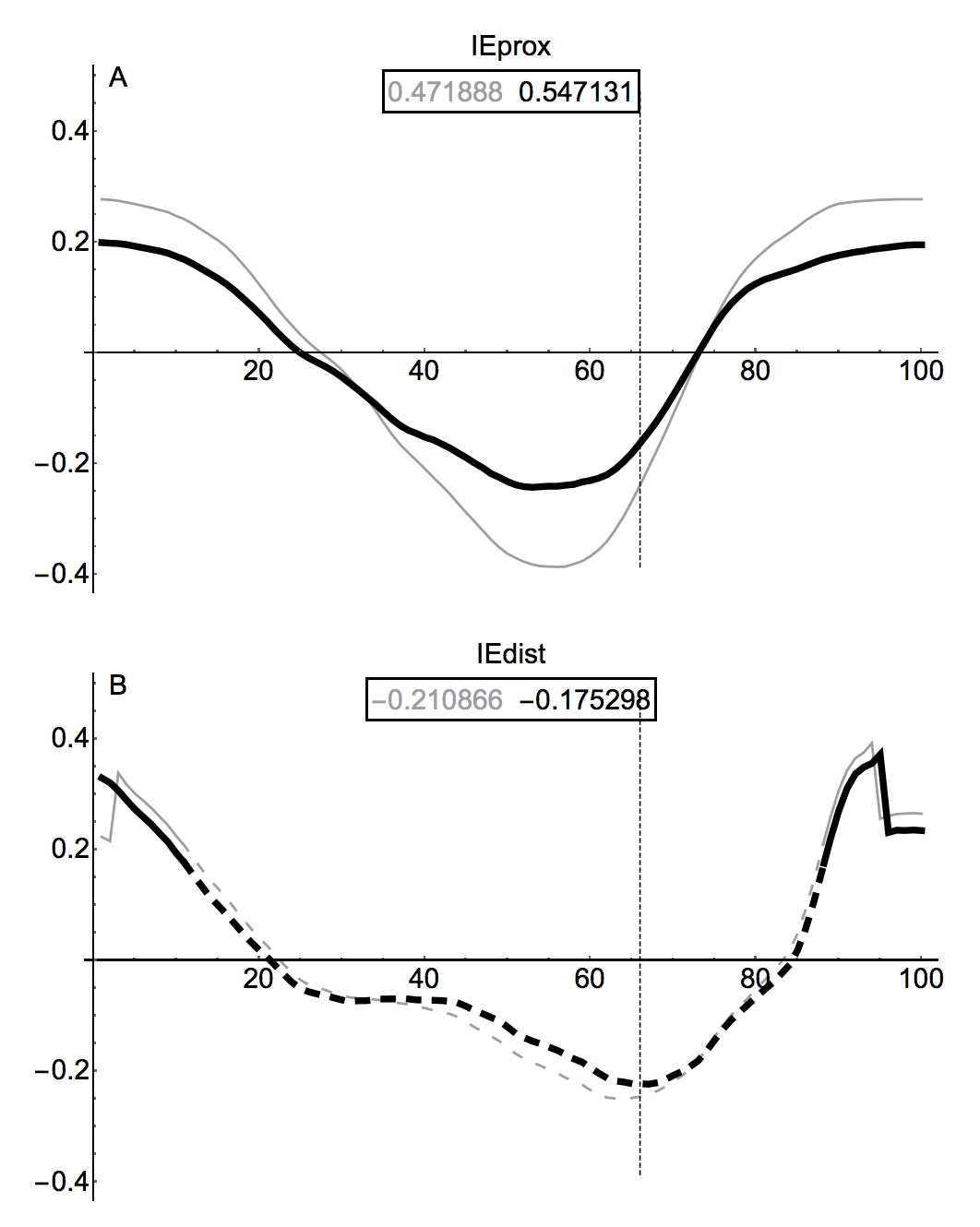

Supplement: Supplementary file 3 [file DataSheet2.ZIP › FigureSI_RUN_LIE_FE_relativeScaling.png]

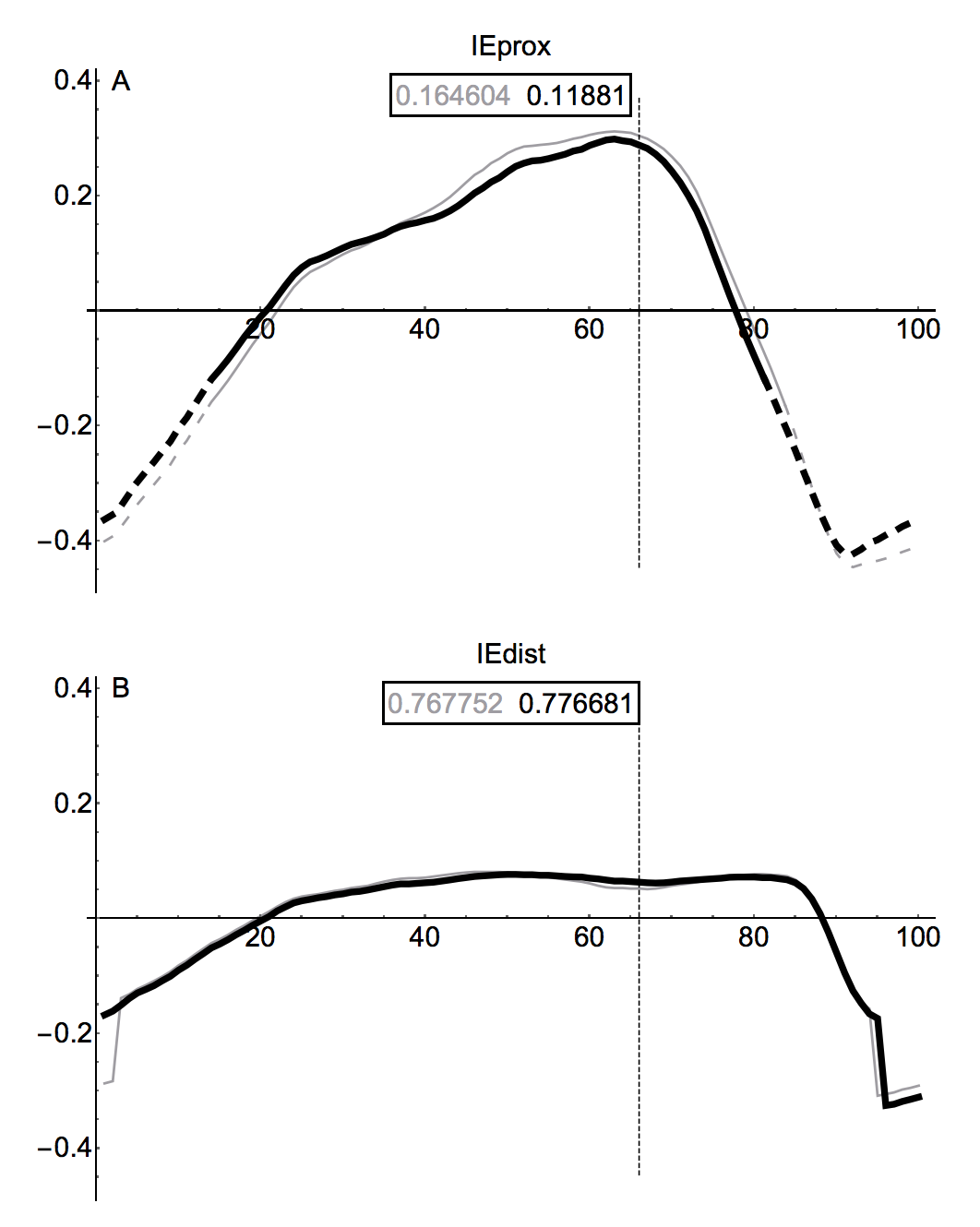

Supplement: Supplementary file 3 [file DataSheet2.ZIP › FigureSI_RUN_LIE_LAR_relativeScaling.png]

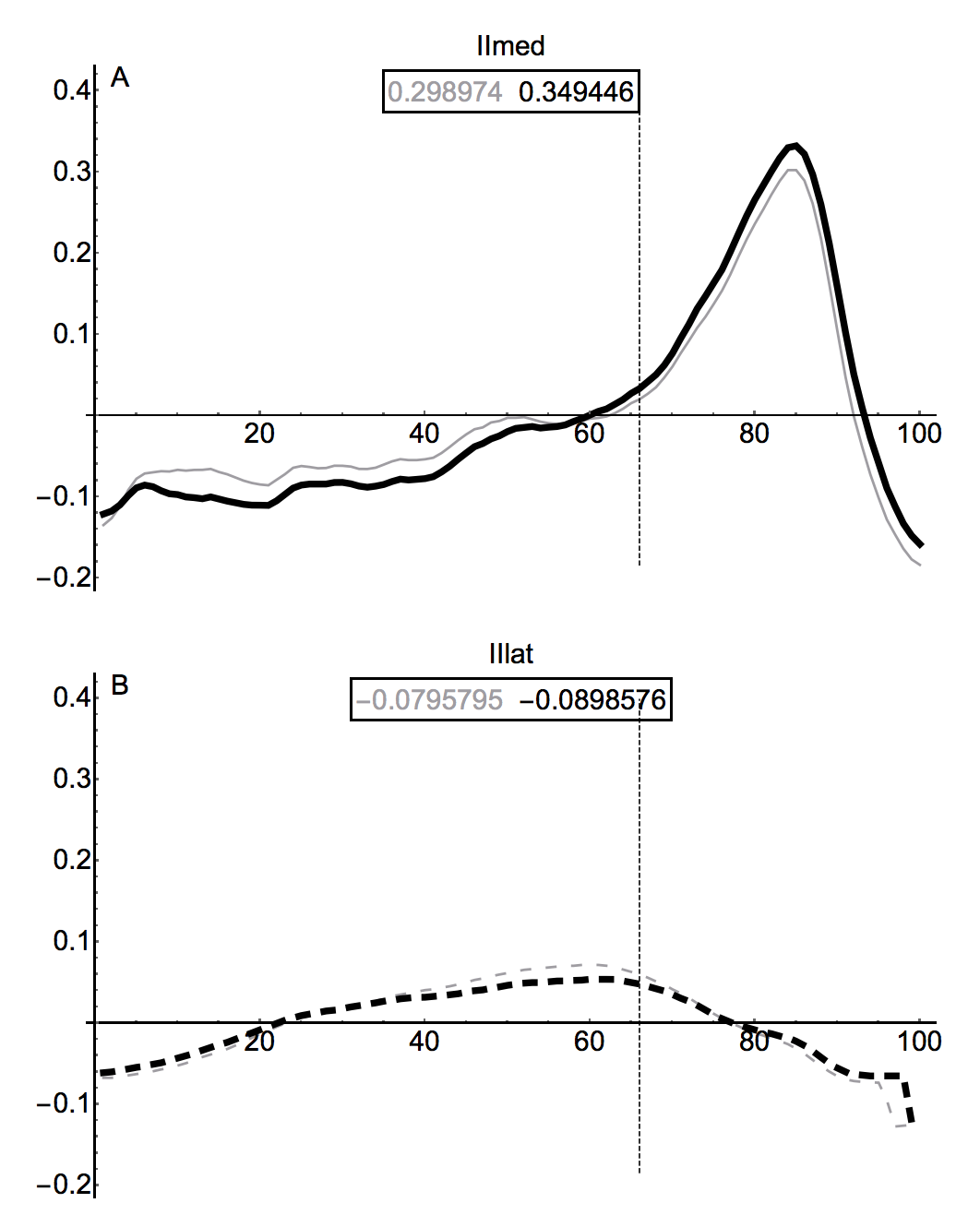

Supplement: Supplementary file 3 [file DataSheet2.ZIP › FigureSI_RUN_LII_AA_relativeScaling.png]

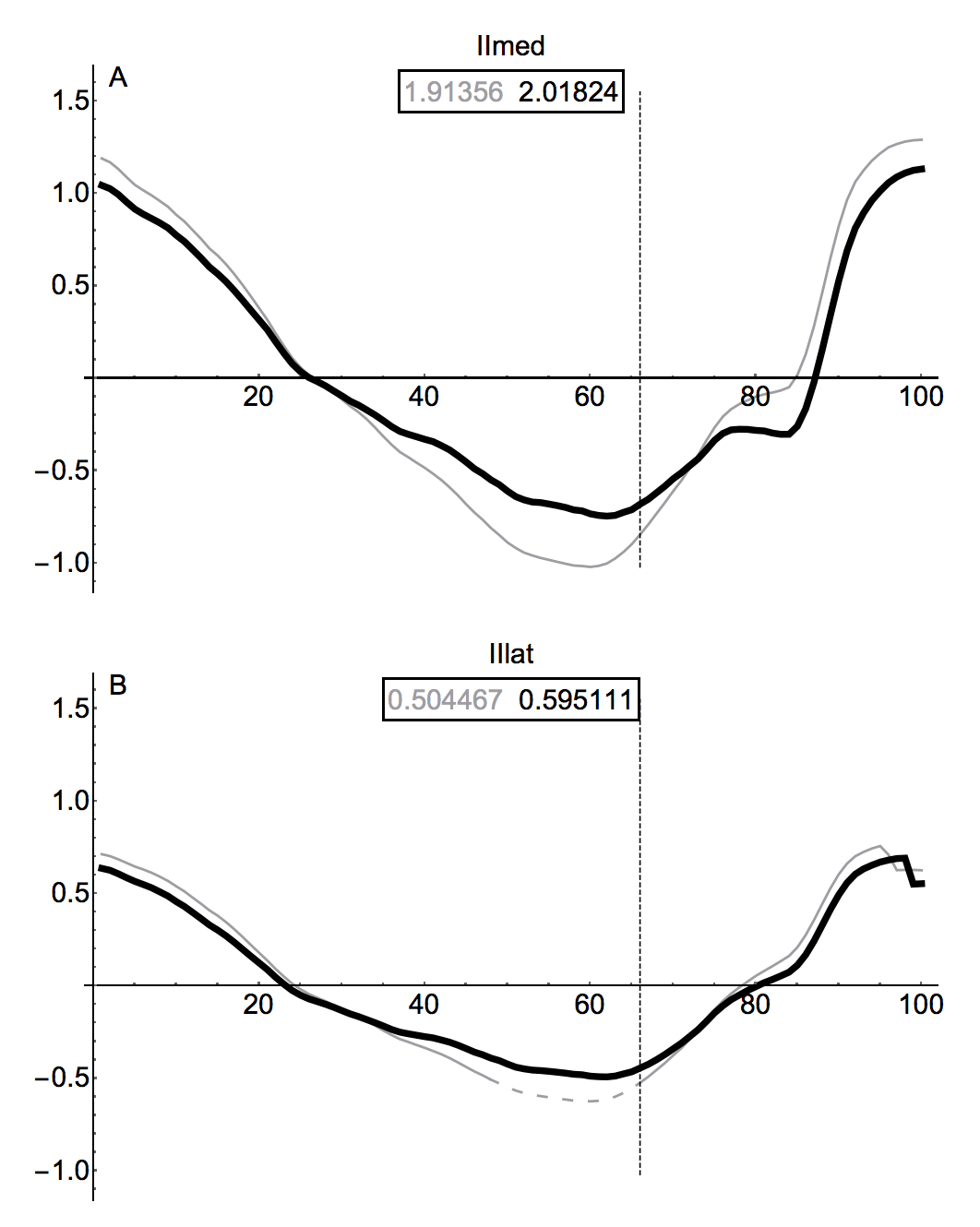

Supplement: Supplementary file 3 [file DataSheet2.ZIP › FigureSI_RUN_LII_FE_relativeScaling.png]

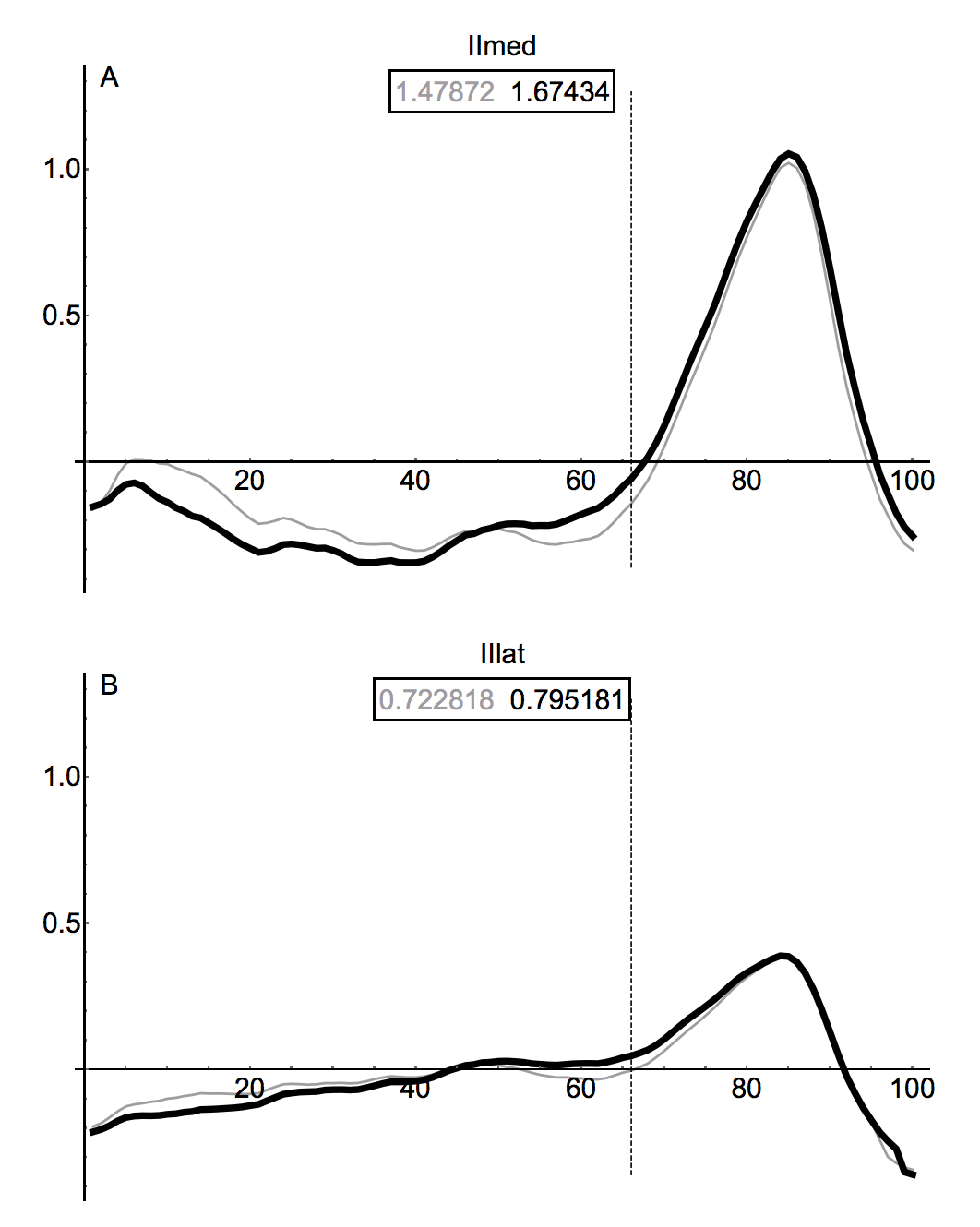

Supplement: Supplementary file 3 [file DataSheet2.ZIP › FigureSI_RUN_LII_LAR_relativeScaling.png]

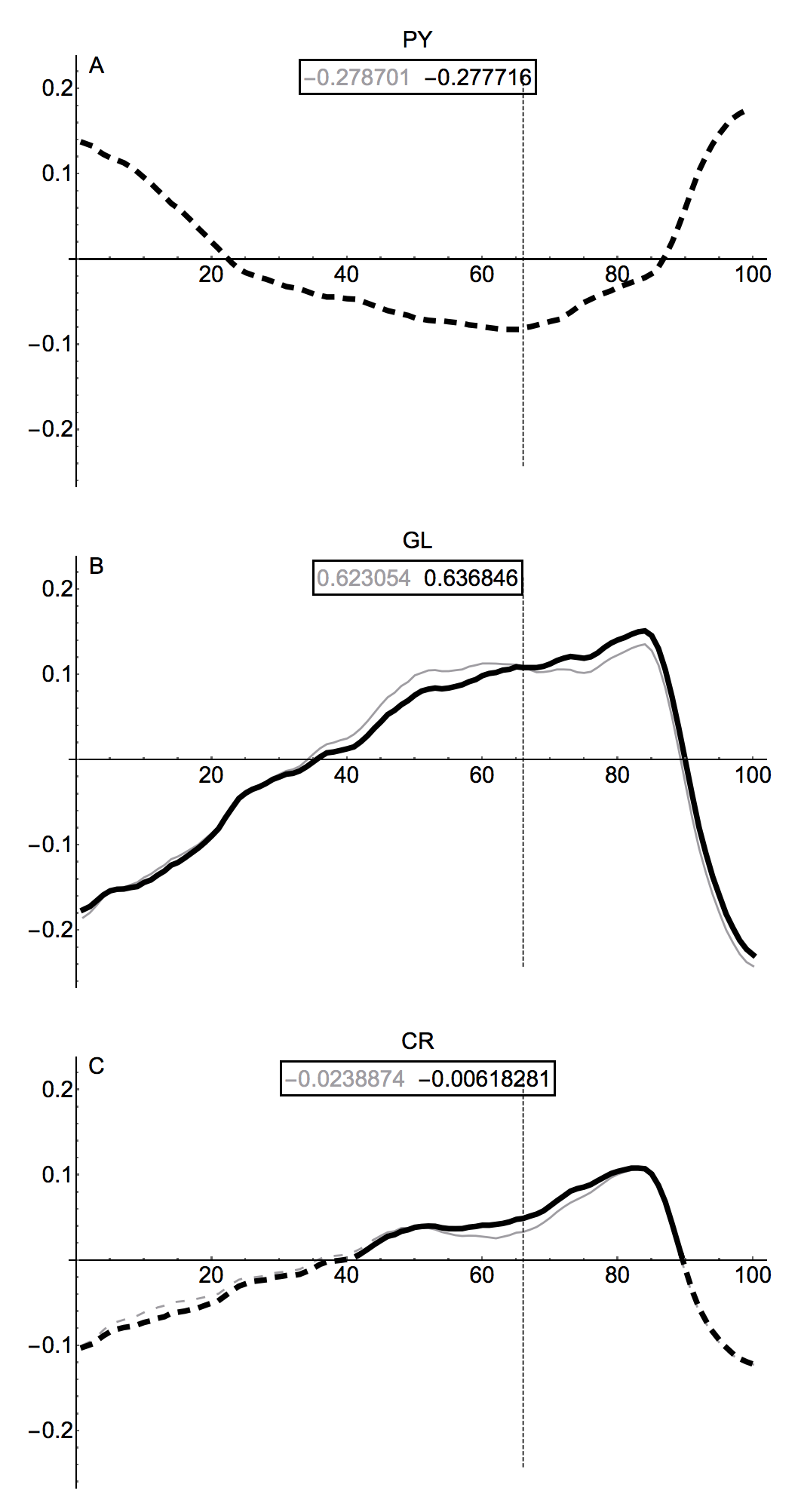

Supplement: Supplementary file 3 [file DataSheet2.ZIP › FigureSI_RUN_misc_AA_relativeScaling.png]

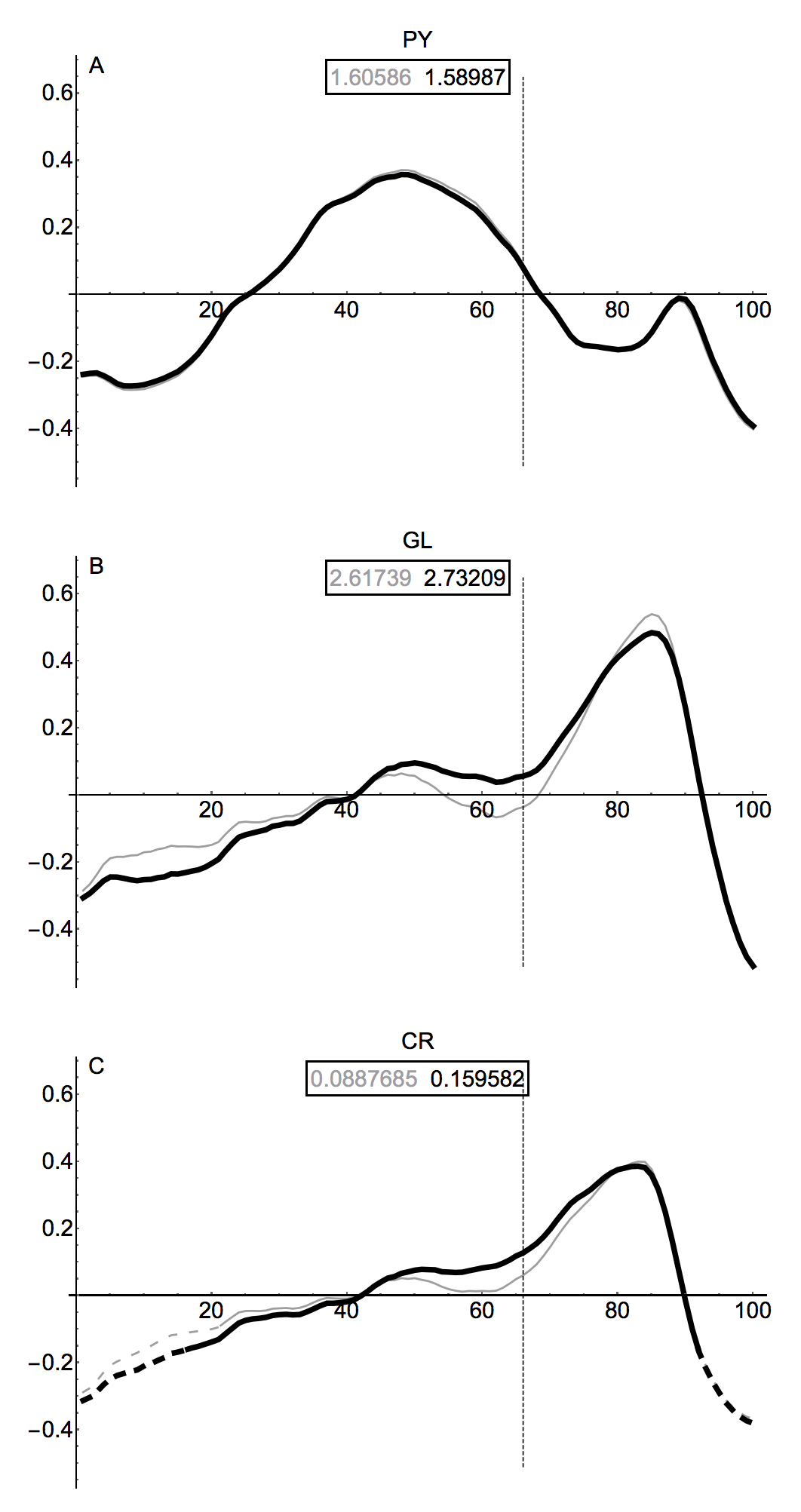

Supplement: Supplementary file 3 [file DataSheet2.ZIP › FigureSI_RUN_misc_LAR_relativeScaling.png]

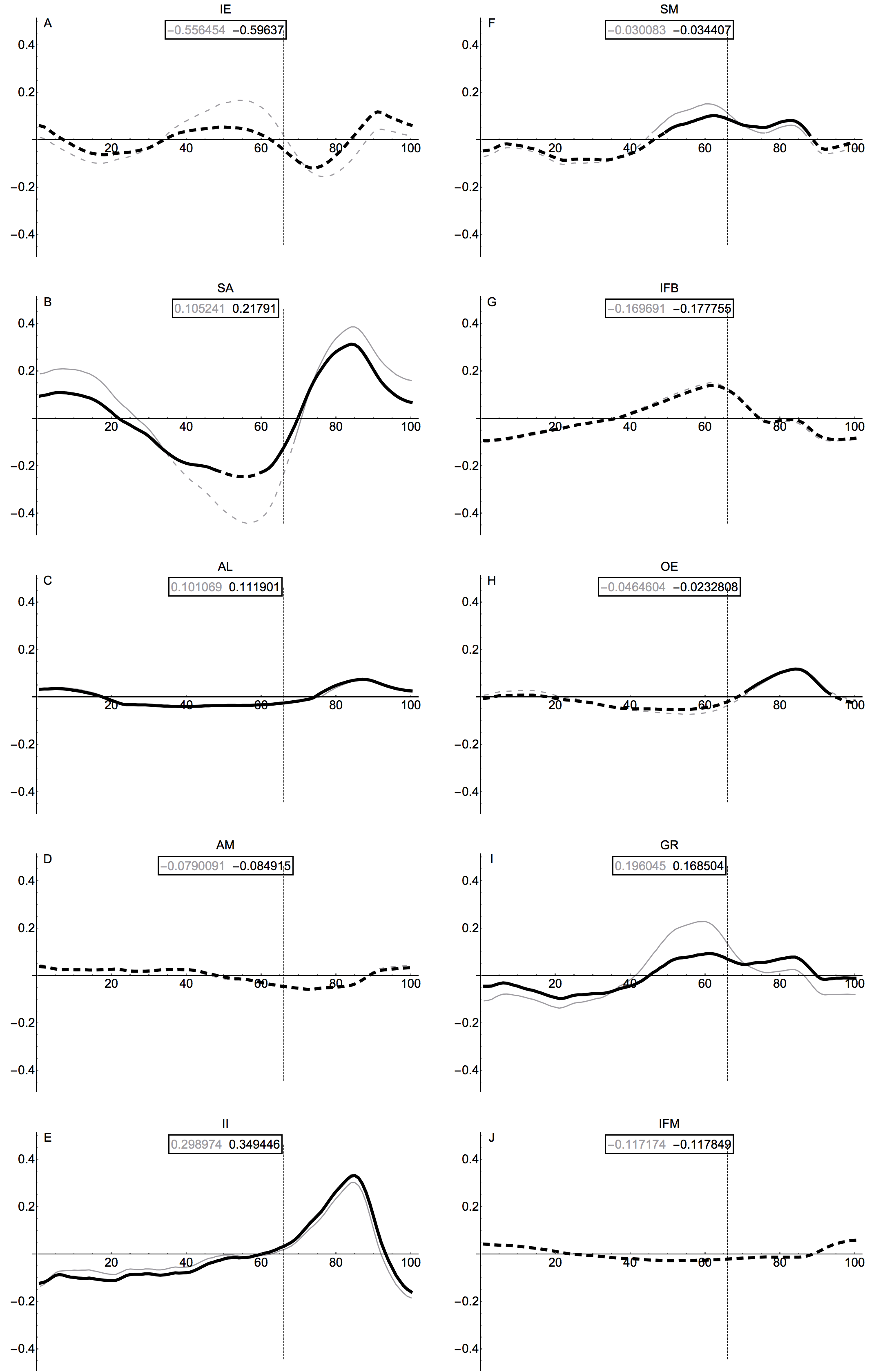

Supplement: Supplementary file 3 [file DataSheet2.ZIP › FigureSI_RUN_pro&ret_AA_relativeScaling.png]

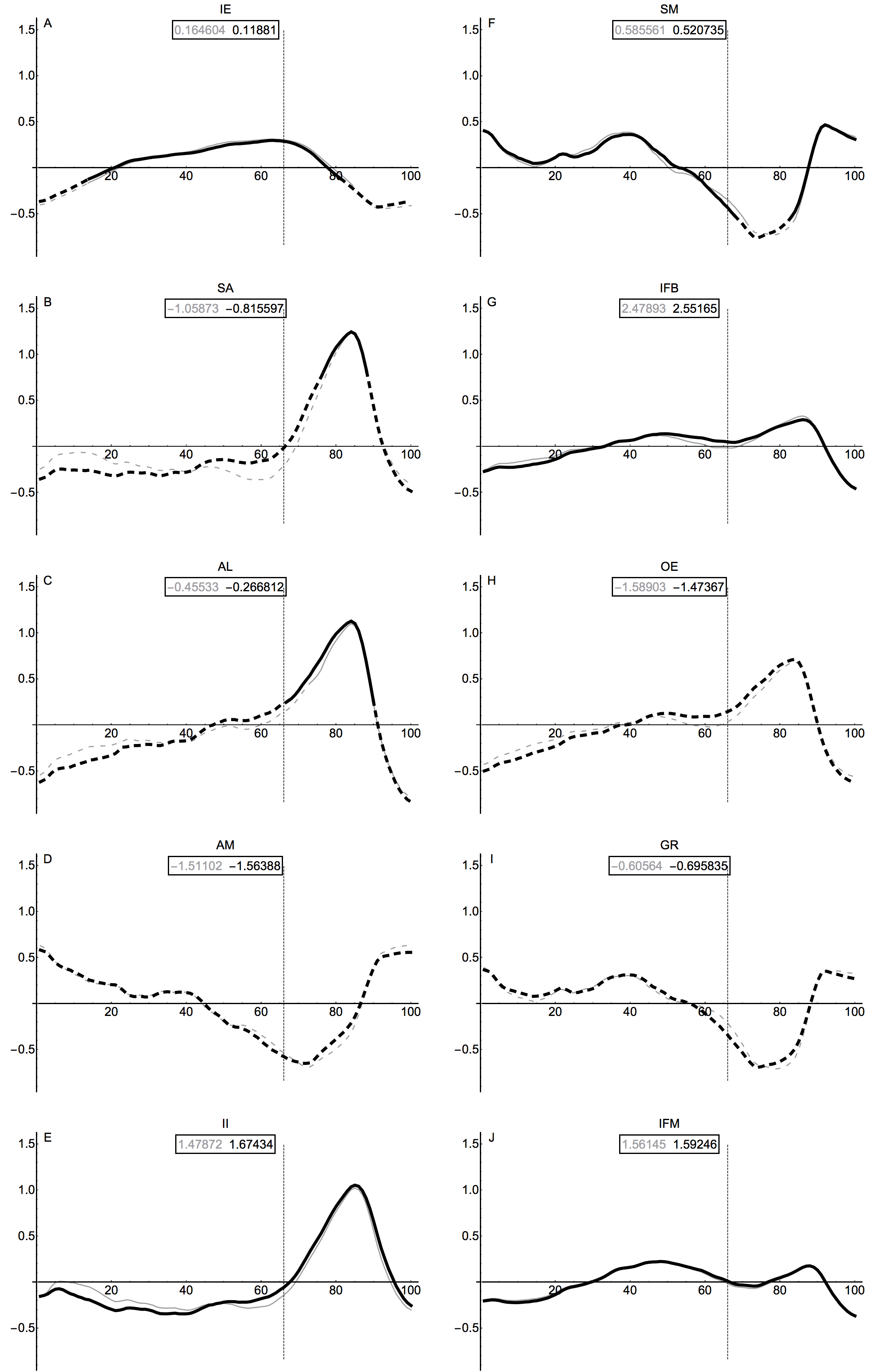

Supplement: Supplementary file 3 [file DataSheet2.ZIP › FigureSI_RUN_pro&ret_LAR_relativeScaling.png]
