# Supplementary material for: Functional Analysis of Anuran Pelvic and Thigh Anatomy Using Musculoskeletal Modelling of Phlyctimantis maculatus
Source: Front Bioeng Biotechnol. 2022 Apr 1;10:806174. doi: 10.3389/fbioe.2022.806174 (PMC9011185; doi:10.3389/fbioe.2022.806174)
Supplement: Supplementary file 4 [file DataSheet1.DOCX]

SI Movie captions

For all movies: Movies show animations captured from the simulation runs. Muscles highlighted are the left coccygeoiliacus (middle region; green), left semimembranosus (yellow) and left adductor longus (blue). For the animations, the frog skin is shown as a transparent layer around bones and muscles; all other muscles have been omitted for clarity.

SI Movie 1. Hypothetical condition HYP_01 (see Table 1 in the main text for further details). The ISX joint is held fully extended and the left leg is held at 10 degrees. Lateral view.

SI Movie 2. Hypothetical condition HYP_01 (see Table 1 in the main text for further details). The ISX joint is held fully extended and the left leg is held at 10 degrees. Top view.

SI Movie 3. Hypothetical condition HYP_02 (see Table 1 in the main text for further details). The ISX joint is held fully extended and the left leg is held at 45 degrees. Lateral view.

SI Movie 4. Hypothetical condition HYP_02 (see Table 1 in the main text for further details). The ISX joint is held fully extended and the left leg is held at 45 degrees. Top view.

SI Movie 5. Hypothetical condition HYP_03 (see Table 1 in the main text for further details). The ISX joint is held fully extended and the left leg is held at 90 degrees. Lateral view.

SI Movie 6. Hypothetical condition HYP_03 (see Table 1 in the main text for further details). The ISX joint is held fully extended and the left leg is held at 90 degrees. Top view.

SI Movie 7. Hypothetical condition HYP_04 (see Table 1 in the main text for further details). The ISX joint is held fully extended and the left leg is held at 135 degrees. Lateral view.

SI Movie 8. Hypothetical condition HYP_04 (see Table 1 in the main text for further details). The ISX joint is held fully extended and the left leg is held at 135 degrees. Top view.

SI Movie 9. Hypothetical condition HYP_05 (see Table 1 in the main text for further details). The ISX joint is held at 22 degrees and the left leg is held at 10 degrees. Lateral view.

SI Movie 10. Hypothetical condition HYP_05 (see Table 1 in the main text for further details). The ISX joint is held at 22 degrees and the left leg is held at 10 degrees. Top view.

SI Movie 11. Hypothetical condition HYP_06 (see Table 1 in the main text for further details). The ISX joint is held at 45 degrees and the left leg is held at 10 degrees. Lateral view.

SI Movie 12. Hypothetical condition HYP_06 (see Table 1 in the main text for further details). The ISX joint is held at 45 degrees and the left leg is held at 10 degrees. Top view.

SI Movie 13. Walking trial RUN_ROT (see Table 1 in the main text for further details). Kinematics input directly from video analysis with no modification. Lateral view.

SI Movie 14. Walking trial RUN_ROT (see Table 1 in the main text for further details). Kinematics input directly from video analysis with no modification. Top view.

SI Movie 15. Walking trial RUN_FIX (see Table 1 in the main text for further details). Kinematics input directly from video analysis, but with pelvic lateral rotation fixed. Lateral view.

SI Movie 16. Walking trial RUN_FIX (see Table 1 in the main text for further details). Kinematics input directly from video analysis, but with pelvic lateral rotation fixed. Top view.

SI figure summary

| Fig name | Raw filename in data repository* |
| --- | --- |
| SI Figure 1 | FigureSI_HYP_CI_LatRotation_relativeScaling |
| SI Figure 2 | FigureSI_HYP_IL_LatRotation_relativeScaling |
| SI Figure 3 | FigureSI_HYP_LAM_AA_relativeScaling |
| SI Figure 4 | FigureSI_HYP_LAM_FE_relativeScaling |
| SI Figure 5 | FigureSI_HYP_LAM_LAR_relativeScaling |
| SI Figure 6 | FigureSI_HYP_LIE_AA_relativeScaling |
| SI Figure 7 | FigureSI_HYP_LIE_FE_relativeScaling |
| SI Figure 8 | FigureSI_HYP_LIE_LAR_relativeScaling |
| SI Figure 9 | FigureSI_HYP_LII_AA_relativeScaling |
| SI Figure 10 | FigureSI_HYP_LII_FE_relativeScaling |
| SI Figure 11 | FigureSI_HYP_LII_LAR_relativeScaling |
| SI Figure 12 | FigureSI_HYP_pro&ret_AA_relativeScaling |
| SI Figure 13 | FigureSI_HYP_pro&ret_LAR_relativeScaling |
| SI Figure 14 | FigureSI_HYP_misc_AA_relativeScaling |
| SI Figure 15 | FigureSI_HYP_misc_LAR_relativeScaling |
| SI Figure 16 | FigureSI_RUN_CI_LatRotation_relativeScaling |
| SI Figure 17 | FigureSI_RUN_IL_LatRotation_relativeScaling |
| SI Figure 18 | FigureSI_RUN_LAM_AA_relativeScaling |
| SI Figure 19 | FigureSI_RUN_LAM_FE_relativeScaling |
| SI Figure 20 | FigureSI_RUN_LAM_LAR_relativeScaling |
| SI Figure 21 | FigureSI_RUN_LIE_AA_relativeScaling |
| SI Figure 22 | FigureSI_RUN_LIE_FE_relativeScaling |
| SI Figure 23 | FigureSI_RUN_LIE_LAR_relativeScaling |
| SI Figure 24 | FigureSI_RUN_LII_AA_relativeScaling |
| SI Figure 25 | FigureSI_RUN_LII_FE_relativeScaling |
| SI Figure 26 | FigureSI_RUN_LII_LAR_relativeScaling |
| SI Figure 27 | FigureSI_RUN_pro&ret_AA_relativeScaling |
| SI Figure 28 | FigureSI_RUN_pro&ret_LAR_relativeScaling |
| SI Figure 29 | FigureSI_RUN_misc_AA_relativeScaling |
| SI Figure 30 | FigureSI_RUN_misc_LAR_relativeScaling |
|  |  |

*HYP = hypothetical conditions; RUN = exemplar walking/running trial; L = Left (e.g. LIE is left iliacus externus); FE = flexion/extension; AA = abduction/adduction; LAR = long axis rotation. Axial muscles belong to set 1, pro&ret to set 2 and misc to set 3 (see below).

Muscle abbreviations and additional notes about plots

Muscle set 1:

Left Iliolumbaris, IL(L), Left coccygeoiliacus, CI(L), right Iliolumbaris, IL(R), right coccygeoiliacus, CI(R)

Muscle set 2:

Iliacus externus, IE, sartorius, SA adductor longus, AL, adductor magnus, AM, Iliacus internus, II semimembranosus, SM, iliofibularis, IFB, obturator externus, OE, gracilis minor and major, GR, iliofemoralis, IFM

Muscle set 3:

pyriformis, PY, gluteus maximus, GL, cruralis, CR

Moment arm signs:

flexion (+); extension (-)

abduction (+); adduction (-)

caudal long-axis rotation (+); cranial long-axis rotation (-)

Solid versus dashed lines represent positive versus negative moment arms, respectively, such that a change from solid to dashed indicates a change in muscle function.

SI Figure 1. Hypothetical conditions. Changes in moment arm (dMA) for the regions of the coccygeoiliacus, CI, muscle versus time are shown for the three hypothetical conditions (see figures in the main paper and text for further details): dorso-ventral iliosacral joint in the extended position (blue), half-flexed (light green) and fully flexed (red).

SI Figure 2. Hypothetical conditions. Changes in moment arm (dMA) for the regions of the iliolumbaris, IL, muscle versus time are shown for the three hypothetical conditions (see figures in the main paper and text for further details): dorso-ventral iliosacral joint in the extended position (blue), half-flexed (light green) and fully flexed (red).

SI Figure 3. Hypothetical conditions. Changes in abduction/adduction moment arm (dMA) for the regions of the adductor magnus, AM, muscle versus time are shown for the four hypothetical conditions (see figures in the main paper and text for further details): leg held at 10 degrees (flexed; blue), 45 degrees (light green), 90 degrees (red) and 135 degrees (grey).

SI Figure 4. Hypothetical conditions. Changes in flexion/extension moment arm (dMA) for the regions of the adductor magnus, AM, muscle versus time are shown for the four hypothetical conditions (see figures in the main paper and text for further details): leg held at 10 degrees (flexed; blue), 45 degrees (light green), 90 degrees (red) and 135 degrees (grey).

SI Figure 5. Hypothetical conditions. Changes in long-axis rotation moment arm (dMA) for the regions of the adductor magnus, AM, muscle versus time are shown for the four hypothetical conditions (see figures in the main paper and text for further details): leg held at 10 degrees (flexed; blue), 45 degrees (light green), 90 degrees (red) and 135 degrees (grey).

SI Figure 6. Hypothetical conditions. Changes in abduction/adduction moment arm (dMA) for the regions of the iliacus externus, IE, muscle versus time are shown for the four hypothetical conditions (see figures in the main paper and text for further details): leg held at 10 degrees (flexed; blue), 45 degrees (light green), 90 degrees (red) and 135 degrees (grey).

SI Figure 7. Hypothetical conditions. Changes in flexion/extension moment arm (dMA) for the regions of the iliacus externus, IE, muscle versus time are shown for the four hypothetical conditions (see figures in the main paper and text for further details): leg held at 10 degrees (flexed; blue), 45 degrees (light green), 90 degrees (red) and 135 degrees (grey).

SI Figure 8. Hypothetical conditions. Changes in long-axis rotation moment arm (dMA) for the regions of the iliacus externus, IE, muscle versus time are shown for the four hypothetical conditions (see figures in the main paper and text for further details): leg held at 10 degrees (flexed; blue), 45 degrees (light green), 90 degrees (red) and 135 degrees (grey).

SI Figure 9. Hypothetical conditions. Changes in abduction/adduction moment arm (dMA) for the regions of the iliacus internus, II, muscle versus time are shown for the four hypothetical conditions (see figures in the main paper and text for further details): leg held at 10 degrees (flexed; blue), 45 degrees (light green), 90 degrees (red) and 135 degrees (grey).

SI Figure 10. Hypothetical conditions. Changes in flexion/extension moment arm (dMA) for the regions of the iliacus internus, II, muscle versus time are shown for the four hypothetical conditions (see figures in the main paper and text for further details): leg held at 10 degrees (flexed; blue), 45 degrees (light green), 90 degrees (red) and 135 degrees (grey).

SI Figure 11. Hypothetical conditions. Changes in long-axis rotation moment arm (dMA) for the regions of the iliacus internus, II, muscle versus time are shown for the four hypothetical conditions (see figures in the main paper and text for further details): leg held at 10 degrees (flexed; blue), 45 degrees (light green), 90 degrees (red) and 135 degrees (grey).

SI Figure 12. Hypothetical conditions. Changes in abduction/adduction moment arm (dMA) for set 2 muscles (see above) versus time are shown for the four hypothetical conditions (see figures in the main paper and text for further details): leg held at 10 degrees (flexed; blue), 45 degrees (light green), 90 degrees (red) and 135 degrees (grey).

SI Figure 13. Hypothetical conditions. Changes in long-axis rotation moment arm (dMA) for set 2 muscles (see above) versus time are shown for the four hypothetical conditions (see figures in the main paper and text for further details): leg held at 10 degrees (flexed; blue), 45 degrees (light green), 90 degrees (red) and 135 degrees (grey).

SI Figure 14. Hypothetical conditions. Changes in abduction/adduction moment arm (dMA) for set 3 muscles (see above) versus time are shown for the four hypothetical conditions (see figures in the main paper and text for further details): leg held at 10 degrees (flexed; blue), 45 degrees (light green), 90 degrees (red) and 135 degrees (grey).

SI Figure 15. Hypothetical conditions. Changes in long-axis rotation moment arm (dMA) for set 3 muscles (see above) versus time are shown for the four hypothetical conditions (see figures in the main paper and text for further details): leg held at 10 degrees (flexed; blue), 45 degrees (light green), 90 degrees (red) and 135 degrees (grey).

SI Figure 16. Walking trial. Changes in moment arm (dMA) versus time are shown for the regions of the coccygeoiliacus, CI, muscle for simulations with a mobile pelvis (natural condition; black) versus a fixed pelvis (simulated condition; grey). See figures in the main paper and text for further details.

SI Figure 17. Walking trial. Changes in moment arm (dMA) versus time are shown for the regions of the iliolumbaris, IL, muscle for simulations with a mobile pelvis (natural condition; black) versus a fixed pelvis (simulated condition; grey). See figures in the main paper and text for further details.

SI Figure 18. Walking trial. Changes in abduction/adduction moment arm (dMA) versus time are shown for the regions of the adductor magnus, AM, muscle for simulations with a mobile pelvis (natural condition; black) versus a fixed pelvis (simulated condition; grey). See figures in the main paper and text for further details.

SI Figure 19. Walking trial. Changes in flexion/extension moment arm (dMA) versus time are shown for the regions of the adductor magnus, AM, muscle for simulations with a mobile pelvis (natural condition; black) versus a fixed pelvis (simulated condition; grey). See figures in the main paper and text for further details.

SI Figure 20. Walking trial. Changes in long-axis rotation moment arm (dMA) versus time are shown for the regions of the adductor magnus, AM, muscle for simulations with a mobile pelvis (natural condition; black) versus a fixed pelvis (simulated condition; grey). See figures in the main paper and text for further details.

SI Figure 21. Walking trial. Changes in abduction/adduction moment arm (dMA) versus time are shown for the regions of the iliacus externus, IE, muscle for simulations with a mobile pelvis (natural condition; black) versus a fixed pelvis (simulated condition; grey). See figures in the main paper and text for further details.

SI Figure 22. Walking trial. Changes in flexion/extension moment arm (dMA) versus time are shown for the regions of the iliacus externus, IE, muscle for simulations with a mobile pelvis (natural condition; black) versus a fixed pelvis (simulated condition; grey). See figures in the main paper and text for further details.

SI Figure 23. Walking trial. Changes in long-axis rotation moment arm (dMA) versus time are shown for the regions of the iliacus externus, IE, muscle for simulations with a mobile pelvis (natural condition; black) versus a fixed pelvis (simulated condition; grey). See figures in the main paper and text for further details.

SI Figure 24. Walking trial. Changes in abduction/adduction moment arm (dMA) versus time are shown for the regions of the iliacus internus, II, muscle for simulations with a mobile pelvis (natural condition; black) versus a fixed pelvis (simulated condition; grey). See figures in the main paper and text for further details.

SI Figure 25. Walking trial. Changes in flexion/extension moment arm (dMA) versus time are shown for the regions of the iliacus internus, II, muscle for simulations with a mobile pelvis (natural condition; black) versus a fixed pelvis (simulated condition; grey). See figures in the main paper and text for further details.

SI Figure 26. Walking trial. Changes in long-axis rotation moment arm (dMA) versus time are shown for the regions of the iliacus internus, II, muscle for simulations with a mobile pelvis (natural condition; black) versus a fixed pelvis (simulated condition; grey). See figures in the main paper and text for further details.

SI Figure 27. Walking trial. Changes in abduction/adduction moment arm (dMA) versus time are shown for the set 2 muscles (see above) for simulations with a mobile pelvis (natural condition; black) versus a fixed pelvis (simulated condition; grey). See figures in the main paper and text for further details.

SI Figure 28. Walking trial. Changes in long-axis rotation moment arm (dMA) versus time are shown for the set 2 muscles (see above) for simulations with a mobile pelvis (natural condition; black) versus a fixed pelvis (simulated condition; grey). See figures in the main paper and text for further details.

SI Figure 29. Walking trial. Changes in abduction/adduction moment arm (dMA) versus time are shown for the set 3 muscles (see above) for simulations with a mobile pelvis (natural condition; black) versus a fixed pelvis (simulated condition; grey). See figures in the main paper and text for further details.

SI Figure 30. Walking trial. Changes in long-axis rotation moment arm (dMA) versus time are shown for the set 3 muscles (see above) for simulations with a mobile pelvis (natural condition; black) versus a fixed pelvis (simulated condition; grey). See figures in the main paper and text for further details.
